# Folic Acid‐Modified Ginger‐Derived Exosome‐Like Nanoparticles Co‐Delivering Sunitinib Suppress Renal Cell Carcinoma via PI3K‐Akt Pathway Inhibition, P‐gp Downregulation, and Macrophage Reprogramming

**DOI:** 10.1002/advs.202512563

**Published:** 2025-11-17

**Authors:** Haoyu Xu, Daixing Hu, Shixue Liu, Lei Yang, Junwu Li, Yuanyuan Bai, Guozhi Zhao, Wei Tang, Li Jiang

**Affiliations:** ^1^ Department of Urology The First Affiliated Hospital of Chongqing Medical University Chongqing 400016 China; ^2^ Department of Urology The Affiliated Yongchuan Hospital of Chongqing Medical University Chongqing 402160 China; ^3^ Department of Breast and Thyroid Surgery The Second Affiliated Hospital of Chongqing Medical University Chongqing 400010 China; ^4^ Department of Urology The Shapingba Hospital Chongqing University (People's Hospital of Shapingba District) Chongqing 400030 China

**Keywords:** ABCB1/P‐gp, ginger‐derived exosome‐like nanoparticles, PI3K‐Akt signaling pathway, renal cell carcinoma, sunitinib, tumor‐associated macrophages

## Abstract

Renal cell carcinoma (RCC) is a malignant tumor with highly recurrent and metastatic capability. The current therapies for RCC are limited by drug resistance and toxic side effects. This study introduces an innovative approach that combines ginger‐derived exosome‐like nanoparticles (GELNs) with sunitinib (Su) and folic acid‐polyethylene glycol (FA‐PEG, FPD) in an active‐passive targeting strategy to explore its multi‐mechanism and synergistic therapeutic effects on RCC. GELNs are extracted via differential centrifugation combined with sucrose gradient ultracentrifugation. Metabolomics and network pharmacology predicted that GELNs may exert their anticancer efficacy via the PI3K‐Akt signaling pathway, which is subsequently validated through in vitro experiments. By loading Su and modifying it with FPD, FPD‐GELNs/Su is constructed. The FPD modification significantly enhanced tumor targeting and amplified the Su sensitivity by reducing ABCB1/P‐gp expression induced by GELNs. In vivo experiments revealed that FPD‐GELNs/Su promoted M1 macrophage polarization and increased immune T‐cell infiltration by remodeling the tumor microenvironment, leading to significant inhibition of tumor growth and lung metastasis without causing notable liver or kidney toxicity. This study integrates network pharmacology with targeted delivery strategies, elucidating the mechanisms by which FPD‐GELNs/Su inhibits RCC progression through multiple pathways, providing new insights for the development of precise and low‐toxicity nano‐therapies.

## Graphical abstract

FPD‐GELNs co‐deliver Su and bioactive components. This active‐passive dual‐targeting strategy suppresses the progression of RCC through the following mechanisms: 1) inhibition of the PI3K‐Akt signaling pathway; 2) downregulation of ABCB1/P‐gp to enhance the chemosensitivity of RCC to Su; and 3) reprogramming of macrophages toward M1 polarization and promotion of CD8^+^ T cell infiltration, thereby exerting potent antitumor activity.

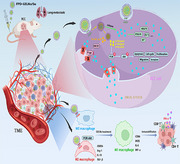

## 1 Introduction

Renal cell carcinoma (RCC) comprises ≈3% of all adult malignancies, and includes subtypes such as clear cell RCC (ccRCC), papillary RCC (pRCC), and chromophobe RCC (chRCC).^[^ ^1^ ^]^ From an epidemiological perspective, a distinct subset of cancers of unknown primary (CUP) exhibiting RCC‐like features has recently emerged. This newly recognized clinical entity is increasingly managed using RCC‐directed therapeutic approaches, thereby contributing to the observed rise in RCC incidence.^[^ ^2^ ^]^ Notably, ccRCC represents the most prevalent subtype, accounting for ≈75% of RCC cases in adults.^[^ ^3^ ^]^ Presently, partial nephrectomy (PN) and radical nephrectomy (RN) are the primary surgical interventions for RCC management.^[^ ^1^ ^]^ However, several critical factors must be considered for surgical options. These include the potential for positive surgical margins and recurrence rate following PN, metabolic and renal function impairment after RN, and the risk of metachronous distant metastasis and associated mortality.^[^ ^4^ ^]^ Consequently, adjuvant therapies such as radiotherapy, chemotherapy, targeted therapy—particularly tyrosine kinase inhibitors (TKIs) such as sunitinib (Su)—and immunotherapy, including cytokines and immune checkpoint inhibitors (ICIs), play essential roles in improving outcomes for RCC patients.^[^ ^4^, ^5^ ^]^

For a substantial subset of RCC patients who are not candidates for immunotherapy, TKI monotherapy remains the standard first‐line treatment. In patients who do not receive TKIs initially, these agents are frequently employed as single‐agent regimens in the second‐line setting. While treatment interruption may be considered in select cases, it should be approached with caution, as such patients typically exhibit shorter progression‐free survival (PFS) compared to those receiving first‐line TKI therapy.^[^ ^6^ ^]^ Despite the clinical benefits of TKIs, their multi‐target inhibition is associated with a range of adverse events, among which hematologic and hepatic toxicities are particularly prominent and necessitate close monitoring. To date, no definitive evidence has established a direct correlation between the severity of these toxicities and therapeutic efficacy. Given that TKIs inhibit vascular endothelial growth factor (VEGF) receptors, they are commonly linked to hypertension and other cardiovascular complications. Beyond these effects, pazopanib has been reported to induce hair depigmentation, gastrointestinal symptoms—including diarrhea, nausea, anorexia, and vomiting—and, in rare instances, posterior reversible encephalopathy syndrome (PRES).^[^ ^7^ ^]^ Furthermore, the development of drug resistance remains inevitable and contributes significantly to suboptimal clinical outcomes.^[^ ^5^, ^8^, ^9^ ^]^

In recent years, the utilization of nanoparticles (NPs) as a novel therapeutic modality has experienced exponential growth. This includes various types such as lipid NPs, polymer NPs, inorganic NPs, and advanced smart NPs, which are employed to target and deliver therapeutic nucleic acids, chemotherapeutic agents, and immunotherapeutic agents to the tumor microenvironment (TME).^[^ ^10^, ^11^ ^]^ These nanocarriers not only enhance therapeutic efficacy but also reduce off‐target toxicity by specifically targeting tumor tissues, cells, and organelles, thereby underscoring the principles of precision medicine.^[^ ^12^, ^13^ ^]^ Despite these advancements, the development of novel nanotherapeutic approaches frequently faces significant challenges due to concerns over immunogenicity, cytotoxicity, and complex manufacturing processes.^[^ ^14^ ^]^ Additionally, stringent regulatory review models have further complicated the clinical translation of most candidate NPs, presenting substantial hurdles for their approval.^[^ ^15^ ^]^

Therefore, plant‐derived exosome‐like NPs (PELNs), which are harmless, environmentally and ecologically friendly, and economically viable, have garnered significant attention in recent studies.^[^ ^16^ ^]^ These NPs efficiently encapsulate bioactive lipids, proteins, RNAs, and other pharmacological molecules from their parental cells while circumventing the quality control challenges associated with whole plants. Consequently, PELNs exhibit considerable clinical potential for treating a wide range of diseases.^[^ ^17^ ^]^ Notably, ginger, an herbaceous plant, has emerged as a prime candidate for PELN production because of its cost‐effectiveness, accessibility, and potentially superior pharmacological activity with PELNs derived from common fruits and vegetables.^[^ ^16^, ^17^ ^]^ Research on ginger‐derived exosome‐like NPs (GELNs) has focused predominantly on their lipid, protein, and RNA contents. Although some studies have examined pharmacological molecules, these investigations have been limited to phenolic compounds such as Gingerols and Shogaols.^[^ ^18^, ^19^ ^]^ To address the limitations and one‐sidedness in the pharmacological investigation of GELNs, we employed network pharmacology to systematically analyze the pharmacological molecules within GELNs as analogous to traditional Chinese medicine formulations. This approach shifts from the “single drug‐single target” paradigm to a “multicomponent, multitarget, multipathway” model, thereby elucidating the complex interactions among drugs, genes, and targets.^[^ ^20^ ^]^

**Scheme 1:**
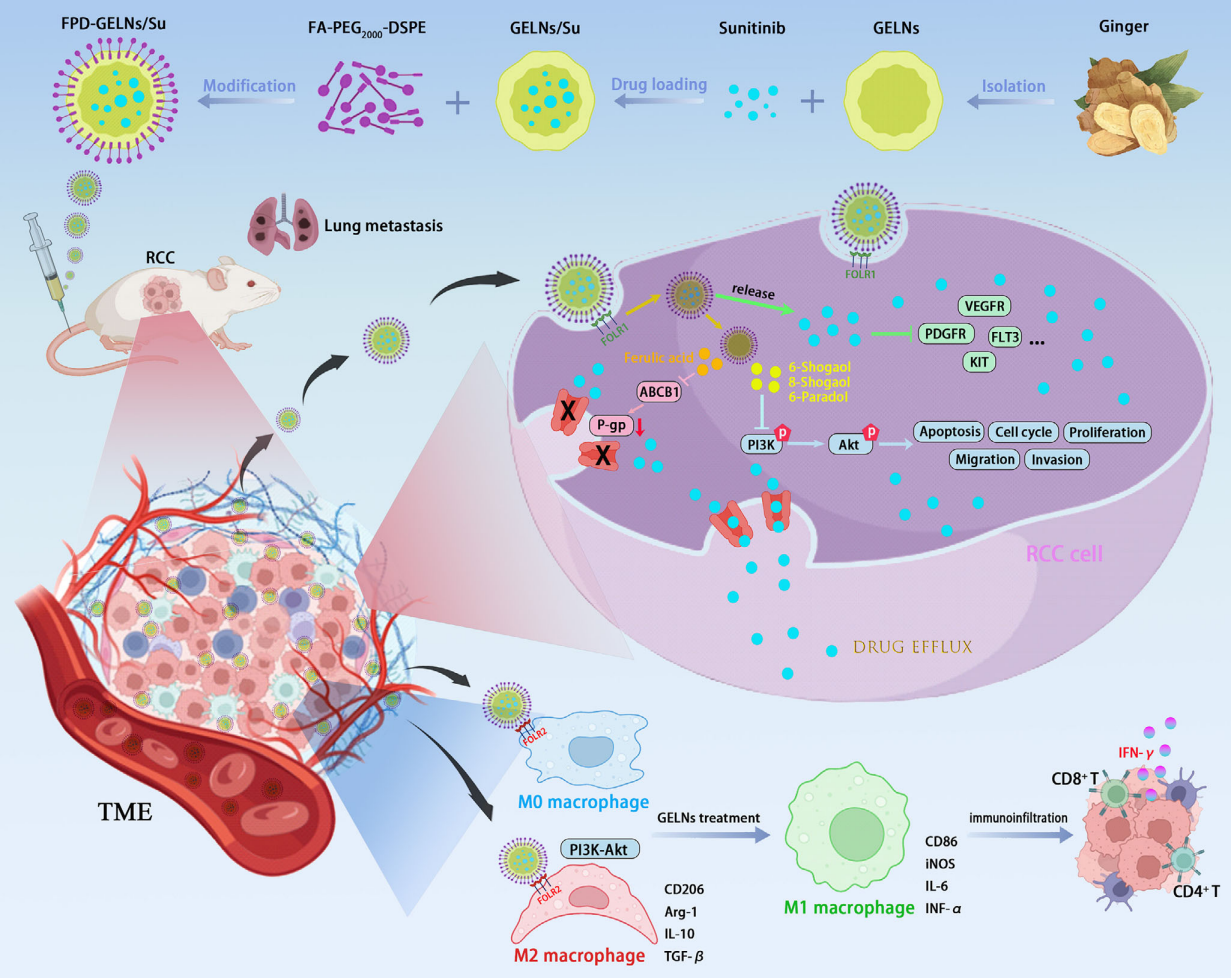
GELNs inhibit RCC through multiple mechanisms: the intrinsic cytotoxicity induced by their carried pharmacological molecules, the sensitization to Su mediated by GELNs, and the immunomodulation of TME facilitated by GELNs.

On the other hand, passive tumor targeting primarily leverages the high permeability of the tumor vasculature and impaired lymphatic drainage to facilitate NP accumulation in solid tumors via the enhanced permeability and retention (EPR) effect, thereby minimizing off‐target effects.^[^ ^21^ ^]^ This approach involves optimizing physical and chemical properties such as particle size, zeta potential, morphology, and drug release patterns, along with the adoption of functionalization strategies to increase pharmacokinetic parameters, increase tumor penetration, and improve stability in biological fluids.^[^ ^22^ ^]^ A well‐established and reliable method is PEGylation and CD47 grafting, which prolongs the NP circulation time by reducing interactions with the mononuclear phagocyte system (MPS).^[^ ^23^ ^]^ Among various targeting strategies, active targeting has emerged as particularly effective. Specific ligands such as folate (FA), hyaluronic acid, transferrin, antibodies, peptides, and aptamers enable precise delivery to target cells. The folate receptor (FOLR) is a well‐known marker for active targeting; FOLR1 is overexpressed in most tumor cells, whereas FOLR2 is predominantly found in activated tumor‐associated macrophages (TAMs) and myeloid‐derived suppressor cells (MDSCs).^[^ ^21^, ^24^ ^]^ The polarization of M1 and M2 macrophages within TAMs plays a crucial role in tumor progression, angiogenesis, and immunosuppression, and is influenced by various pharmacological molecules and PLENs containing pharmacological molecules.^[^ ^25^, ^26^, ^27^, ^28^ ^]^ Studies have shown that exosome‐like NPs (ELNs) derived from Artemisia annua can induce cGAS‐STING pathway‐mediated remodeling of TAMs, promoting colon cancer regression.^[^ ^27^ ^]^ Similarly, ginseng‐derived ELNs reportedly enhance glioma treatment by recruiting M1 macrophages to the TME.^[^ ^28^ ^]^ In addition to their endogenous components, PELNs can also carry exogenous therapeutic agents through encapsulation driven by diffusion and lipophilic interactions between drug molecules and the lipid bilayer, enabling synergistic endogenous‐exogenous treatments.^[^ ^14^ ^]^ In this study, we introduce the concept of network pharmacology for PELNs and integrate it with FA‐PEG‐based active‐passive targeting strategies. By loading Su onto GELNs for dual targeting of tumor cells and TAMs, we aim to investigate its therapeutic efficacy in RCC and provide a direction for future precision treatment of RCC. The specific mechanism diagram of this study is illustrated in **Scheme** **1**.

## 2 Results

### 2.1 Identification and Characterization of GELNs

**Figure 1:**
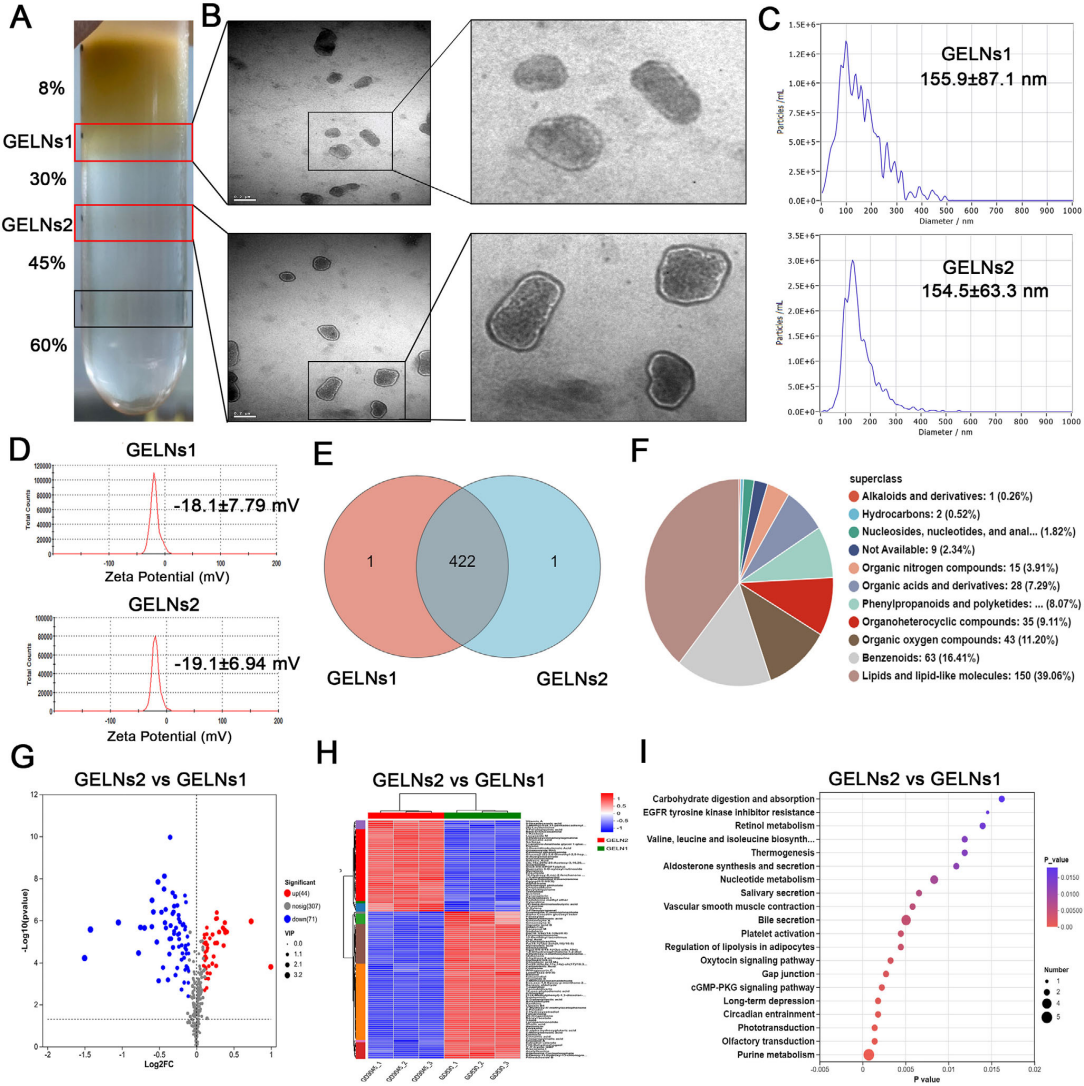
Extraction, characterization, and metabolomic analysis of GELNs. A) Purification of GELNs1 and GELNs2 via sucrose gradient centrifugation (n = 3 independent experiments). B) TEM, C) NTA, and D) zeta potential were used to characterize GELNs1 and GELNs2 (n = 3 independent experiments). E) Venn diagram illustrating the intersection of metabolites between GELNs1 and GELNs2. F) Pie chart depicting the HMDB classification of GELN metabolites. G) Volcano plot, H) heatmap, and I) KEGG pathway enrichment analysis of differentially expressed metabolites between GELNs2 and GELNs1.

Following sucrose gradient centrifugation purification, the 8/30% layer was designated as GELNs1, and the 30/45% layer was designated GELNs2 (**Figure** **1**A). Transmission electron microscope (TEM) revealed that both GELNs1 and GELNs2 exhibited spherical vesicles with bilayer membranous structures, ranging in size from 100 to 200 nm (Figure 1B). Nanoparticle tracking analysis (NTA) revealed that the mean particle size of GELNs1 was 155.9 ± 87.1 nm, whereas that of GELNs2 was 154.5 ± 63.3 nm (Figure 1C). Additionally, the zeta potential measurements revealed values of −18.1 ± 7.79 mV for GELNs1 and −19.1 ± 6.94 mV for GELNs2 (Figure 1D), which are consistent with previously reported literature.^[^ ^17^ ^]^ No significant differences were observed in either particle size or zeta potential between GELNs1 and GELNs2.

Metabolomic analysis revealed that GELNs1 and GELNs2 presented highly similar metabolite profiles (Figure 1E), including compounds such as 6‐Gingerol, 8‐Gingerol, 10‐Gingerol, and 6‐Shogaol, which have been previously reported in the literature.^[^ ^17^, ^18^, ^29^ ^]^ Given their compositional similarity, GELNs1 and GELNs2 may exert comparable pharmacological effects in disease contexts. Therefore, a total of 422 metabolites commonly detected in both GELNs1 and GELNs2 were integrated as the representative metabolome of GELNs for downstream analyses. According to the Human Metabolome Database (HMDB) compound classification, the primary constituents of GELNs were lipids and lipid‐like molecules (39.06%), benzenoids (16.41%), and organic oxygen compounds (11.2%) (Figure 1F). Detailed information regarding all identified metabolites is provided (Table S1, Supporting Information). The Kyoto Encyclopedia of Genes and Genomes (KEGG) pathway analysis revealed that the major metabolic pathways involved in GELNs were lipid metabolism, amino acid metabolism, carbohydrate metabolism, and nucleotide metabolism (Figure S1A, Supporting Information). These findings suggest that the overall progression and development of cancer are closely associated with the metabolites present in GELNs.

Compared with GELNs1, GELNs2 upregulated 44 metabolites and downregulated 71 metabolites (Figure 1G, H). The differentially abundant metabolites were enriched primarily in pathways such as EGFR tyrosine kinase inhibitor resistance, retinol metabolism, and thermogenesis (*p* < 0.05; Figure 1I). Notably, the HMDB compound classification of the differentially abundant metabolites was similar to that of the overall GELN metabolome (Figure 1F; Figure S1B, Supporting Information). The results of the correlation analysis of the differentially abundant metabolites are presented (Figure S1C, Supporting Information).

### 2.2 Prediction of the Targets Influencing the Progression of RCC by GELNs and the Regulatory Network Involved

**Figure 2:**
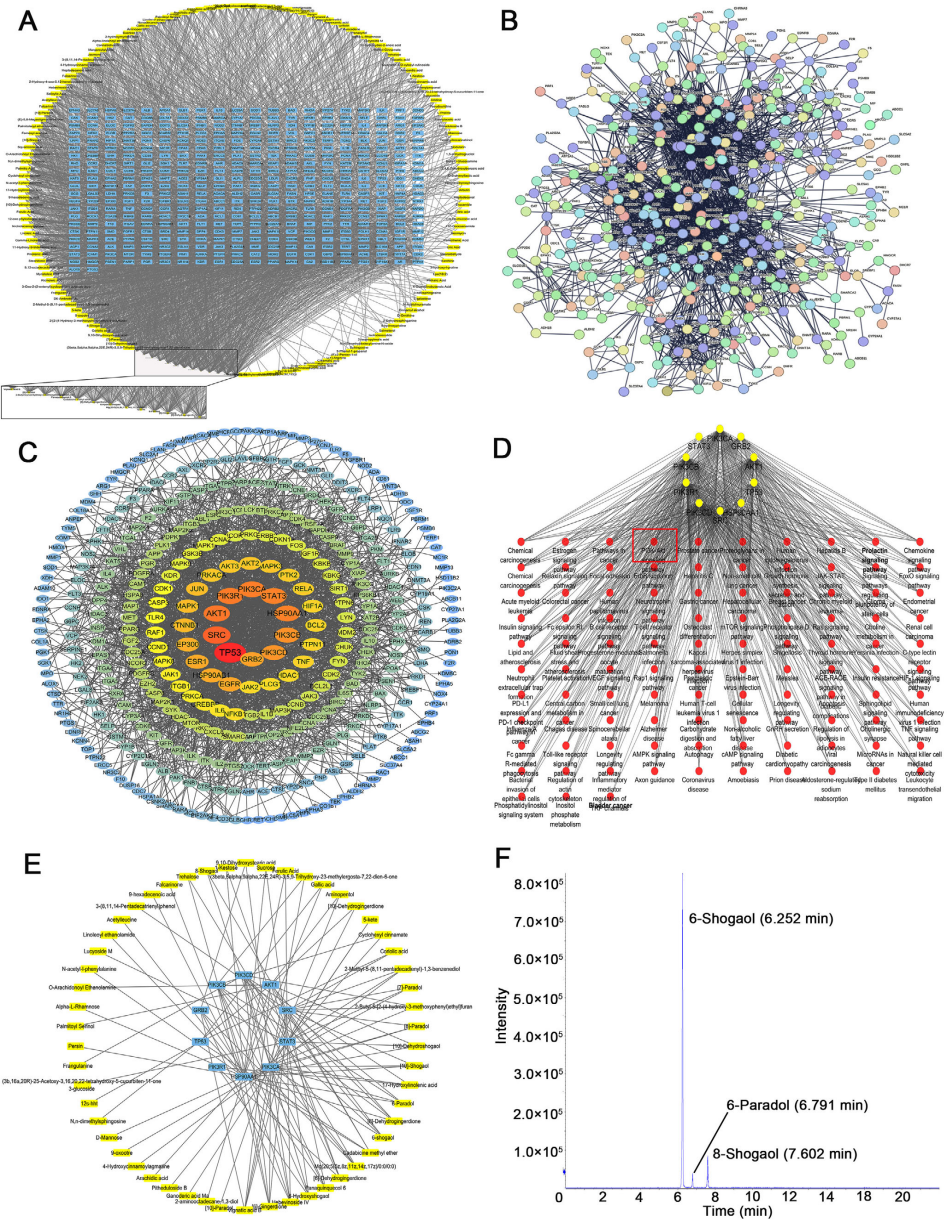
Exploration of the mechanism by which GELNs affect human RCC. A) The drug regulatory network of GELNs on human RCC. B) PPI network of GELN‐related target genes in human RCC constructed via the STRING database. C) Gene‐gene interaction network ranked by degree centrality generated by Cytoscape. D) Gene‐pathway‐related connection diagram ranked by degree centrality created via the ClueGO and CluePedia plugins in Cytoscape. E) Drug regulatory network of compounds targeting human CHGs. F) Positive ion mode HPLC employed to detect 6‐Shogaol, 8‐Shogaol, and 6‐Paradol (n = 3 independent experiments).

Based on the “carcinogenicity < 0.3” scoring criterion from the ADMETlab 3.0 database, we identified 170 compounds from GELNs that potentially possess biological anticancer activity. After target prediction and removal of missing and duplicate data via the TCMSP and SwissTargetPrediction databases, a total of 164 compounds with 1146 associated targets were obtained. Additionally, the DrugBank 6.0, GeneCards, OMIM, and TTD databases provided 2143 human RCC‐related targets. The intersection of these datasets revealed 422 potential targets that may be regulated by GELNs in the context of human RCC development (Figure S2A, Supporting Information). The Gene Ontology (GO) and KEGG pathway enrichment analysis of these 422 targets (Figure S2B,C, Supporting Information) revealed significant enrichment in RCC‐associated signaling pathways such as the PI3K‐Akt, MAPK, and EGFR pathways (*p* < 0.05).^[^ ^30^, ^31^, ^32^ ^]^ Similarly, 326 potential targets that might influence mouse RCC via GELNs were identified (Figure S2D, Supporting Information), and corresponding mouse GO and KEGG enrichment analyses were performed (*p* < 0.05; Figure S2E,F, Supporting Information). Using these data, we constructed and visualized a compound‐human RCC target regulatory network with Cytoscape (**Figure** **2**A). Among the compounds, 6‐Shogaol, 6‐Dehydrogingerdione, 6‐Dingerdione, and 6‐Paradol presented the greatest number of connected targets, suggesting that they may play critical roles in the biological behavior of RCC (Table S2, Supporting Information). Furthermore, a compound‐mouse RCC target regulatory network was constructed (Figure S3A and Table S3, Supporting Information).

### 2.3 Screening and Mechanistic Exploration of Core Hub Genes (CHGs)

To further identify the CHGs regulated by GELNs in RCC, we imported 422 targets into the STRING database to construct a protein‐protein interaction (PPI) network (Figure 2B). After removing isolated nodes, we utilized Cytoscape to construct a gene‐gene interaction network based on the PPI data (Figure 2C; 384 nodes and 2105 edges). The top 10 genes ranked by degree centrality were selected as human CHGs, including TP53 (degree = 75), SRC (degree = 59), AKT1 (degree = 53), PIK3R1 (degree = 52), PIK3CA (degree = 51), STAT3 (degree = 50), HSP90AA1 (degree = 50), PIK3CB (degree = 46), PIK3CD (degree = 45), and GRB2 (degree = 42) (Table S4, Supporting Information). Similarly, via the same methodology, we constructed a mouse PPI network (Figure S3B, Supporting Information) and a corresponding gene‐gene interaction network (Figure S3C, Supporting Information; 274 nodes and 780 edges), from which mouse CHGs were also identified (Table S5, Supporting Information).

To elucidate the mechanisms by which CHGs exert their biological effects through specific pathways, we utilized the ClueGO + CluePedia plugin in Cytoscape to construct a gene‐pathway interaction map. The PI3K‐Akt signaling pathway ranked prominently in both the human and mouse pathway enrichment analyses (*p* < 0.05; Figure 2D; Figure S3D, Supporting Information; Degree of gene‐pathway connectivity: human = 8, mouse = 6). Ultimately, the regulatory network of RCC‐related targets by GELNs was extended to generate the compound‐human CHGs (Figure 2E) and compound‐mouse CHGs (Figure S4E, Supporting Information) relationship network.

Among the 10 human CHGs, 8 were associated with the PI3K‐Akt signaling pathway. In the mouse counterparts, 6 out of 10 CHGs were linked to this pathway. From the compounds targeting multiple PI3K‐Akt‐related CHGs, we selected three bioactive compounds that exhibit multi‐target interactions with PI3K‐Akt‐related CHGs and are commonly present in ginger tissue for quantitative analysis: 6‐Shogaol (targeting human PI3KCA, PI3KCB, and PI3KCD; and mouse Hsp90aa1 and Pi3kca), 8‐Shogaol (targeting human PI3KCA; and mouse Hsp90aa1 and Pi3kca), and 6‐Paradol (targeting human HSP90AA1, PI3KCA, and PI3KCB; and mouse Hsp90aa1, Pi3kca, and Mapk3). Based on the HPLC‐derived standard curves (Figure S4A–C, Supporting Information), the concentrations of 6‐Shogaol, 8‐Shogaol, and 6‐Paradol in ginger juice were determined to be 4.07, 0.11, and 0.67 µg mg^−1^, respectively. In GELNs, the corresponding levels were 1.07, 0.38, and 1.05 µg mg^−1^, respectively. These results suggest that the levels of 8‐Shogaol and 6‐Paradol are relatively higher in GELNs compared to ginger juice (Figure 2F; Figure S3F, Supporting Information).

### 2.4 Bioinformatics, Molecular Docking, and Molecular Dynamics (MD) of CHGs

The differential expression analysis and survival curves of CHGs in tumor tissues versus adjacent nontumor tissues across the main subtypes of RCC were presented: ccRCC (Figure S5A, Supporting Information), pRCC (Figure S5B, Supporting Information), and chRCC (Figure S5C, Supporting Information). These findings indicate that specific genes are significantly downregulated or upregulated in tumor tissues, and may play a crucial role in the pathogenesis of RCC.

**Figure 3:**
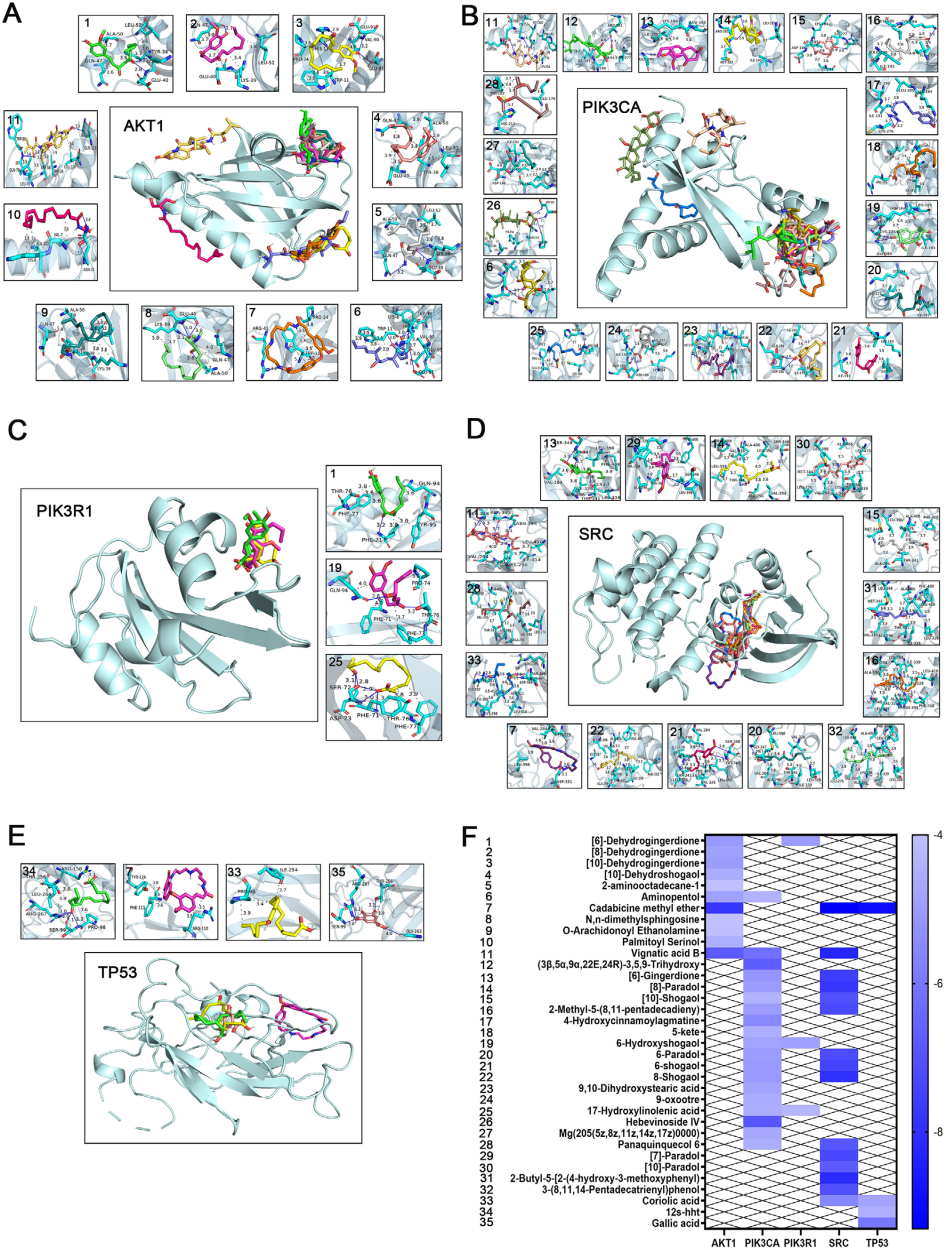
Molecular docking analysis of the top 5 CHGs, ranked by degree, with associated compounds. Molecular docking of compounds with A) AKT1, B) PI3KCA, C) PI3KR1, D) SRC, E) TP53, and F) corresponding binding energy heatmaps. The blue lines represent hydrogen bonds, the gray dashed lines represent hydrophobic interactions, the light green dashed lines represent *π*–*π* stacking (parallel), the dark green dashed lines represent *π*–*π* stacking (perpendicular), and the yellow dashed lines represent salt bridges.

**Figure 4:**
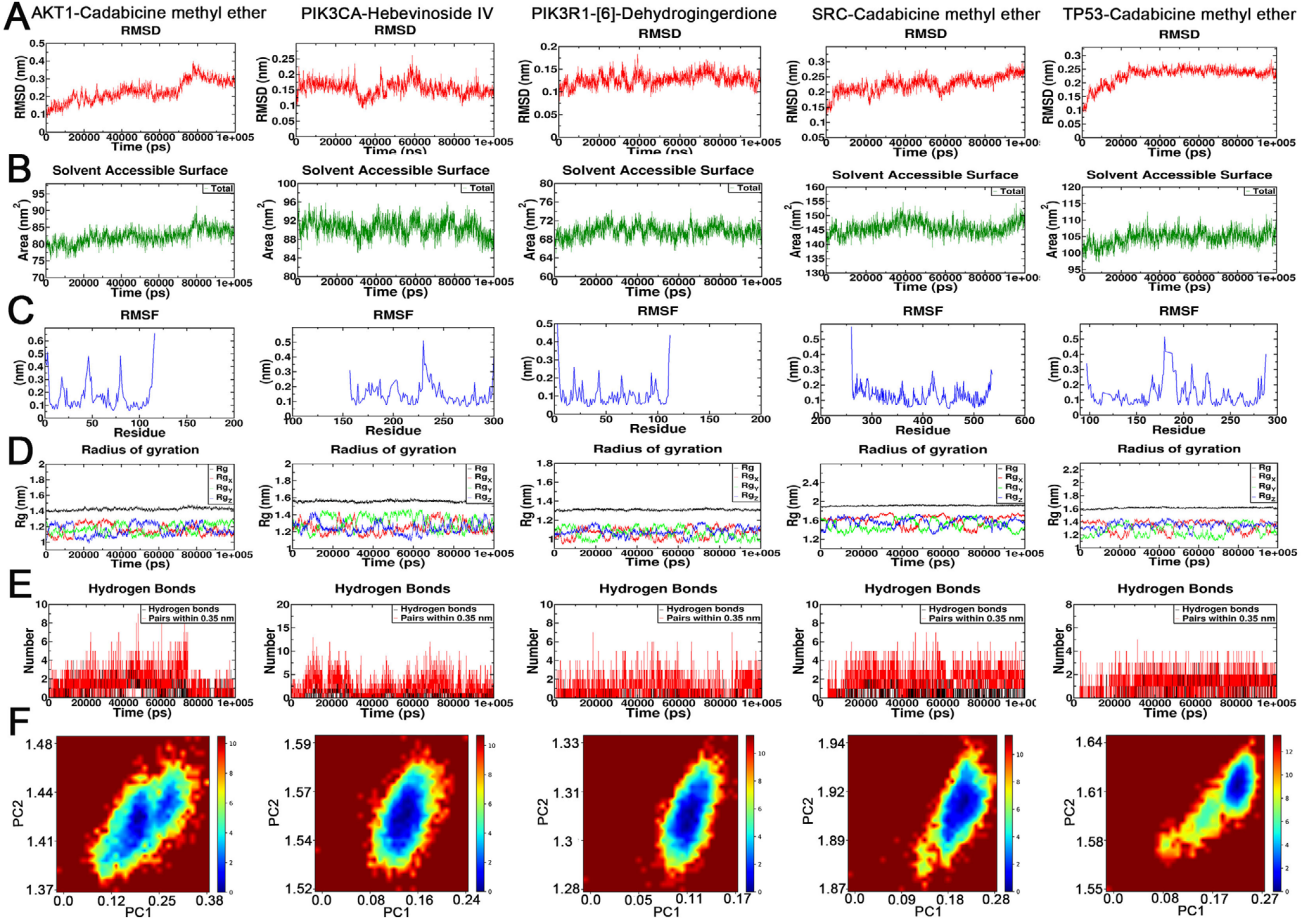
Molecular dynamics simulations were performed to analyze the A) RMSD, B) SASA, C) RMSF, D) Rg, E) the number of hydrogen bonds, and F) free energy landscape for five protein complexes: AKT1‐Cadabicine methyl ether, PIK3CA‐Hebevinoside IV, PIK3R1‐^[^ ^6^ ^]^‐Dehydrogingerdione, SRC‐Cadabicine methyl ether, and TP53‐Cadabicine methyl ether.

Molecular docking was conducted via multiple software tools and databases. The top five genes ranked by degree centrality were identified as the primary receptors for molecular docking: TP53, SRC, AKT1, PIK3R1, and PIK3CA. The PDB IDs of these receptors were retrieved from the PDB database. AutoDock Tools were employed to identify the positions of the active sites and define the grid dimensions (Table S6, Supporting Information). Molecular docking simulations for 52 compound‐receptor pairs were performed via AutoDock Vina, with the resulting 3D docking diagrams presented (**Figure** **3**A–E). Additionally, the binding energy heatmap shows that the binding energies of most compound‐receptor pairs are lower than −5 kcal mol^−1^ (**Figure** **4**F; Table S7, Supporting Information). Notably, 20 compound‐receptor pairs exhibit binding energies below −6 kcal mol^−1^. The average binding energies for TP53, SRC, AKT1, PIK3R1, and PIK3CA with their respective compounds are −6.08, −7.45, −5.05, −4.73, and −5.1 kcal mol^−1^, respectively. These results indicate strong binding stability between the compounds and their target proteins, suggesting potential activation of downstream signaling pathways.

Based on the molecular docking results, compounds exhibiting the lowest docking free energy for each CHG were selected for MD simulation. The five MD combinations included AKT1‐Cadabicine methyl ether, PIK3CA‐Hebevinoside IV, PIK3R1‐^[^ ^6^ ^]^‐Dehydrogingerdione, SRC‐Cadabicine methyl ether, and TP53‐Cadabicine methyl ether. The results revealed that the root mean square deviation (RMSD) values of the five ligand‐receptor pairs fluctuated within a narrow range, indicating stable protein‐small molecule structures (Figure 4A). The solvent‐accessible surface area (SASA) values remained stable throughout the simulation with no significant changes in overall trend, suggesting minimal impact of binding on the surface properties and stability of the protein (Figure 4B). Root mean square fluctuation (RMSF) quantifies the flexibility of the protein by calculating the root mean square fluctuation of each amino acid residue and serves as a critical indicator of local dynamics within the protein structure. Residues at peak positions exhibited higher fluctuations and flexibility, potentially corresponding to flexible regions or functional sites of the protein. In contrast, residues with lower fluctuations demonstrated greater stability (Figure 4C). Radius of gyration (Rg) analysis indicated that the protein maintained overall structural balance during the MD simulation, confirming the stability of its global conformation without signs of loosening or unfolding (Figure 4D). Hydrogen bonds represent one of the key forces in protein‐ligand interactions and are closely associated with electrostatic interactions, reflecting their strength. The number of hydrogen bonds between the five ligand‐receptor pairs was relatively low yet stable, generally ranging from 2 to 5, suggesting moderate binding specificity (Figure 4E). Finally, the free energy landscape was constructed based on RMSD and Rg indicators to reflect the interactions and energy distribution within the binding system. The results demonstrated that all five ligand‐receptor binding systems exhibited stable and singular conformations, rather than being composed of multiple scattered conformations (Figure 4F).

### 2.5 Investigation of the In Vitro Anticancer Efficacy and Mechanism of GELNs

**Figure 5:**
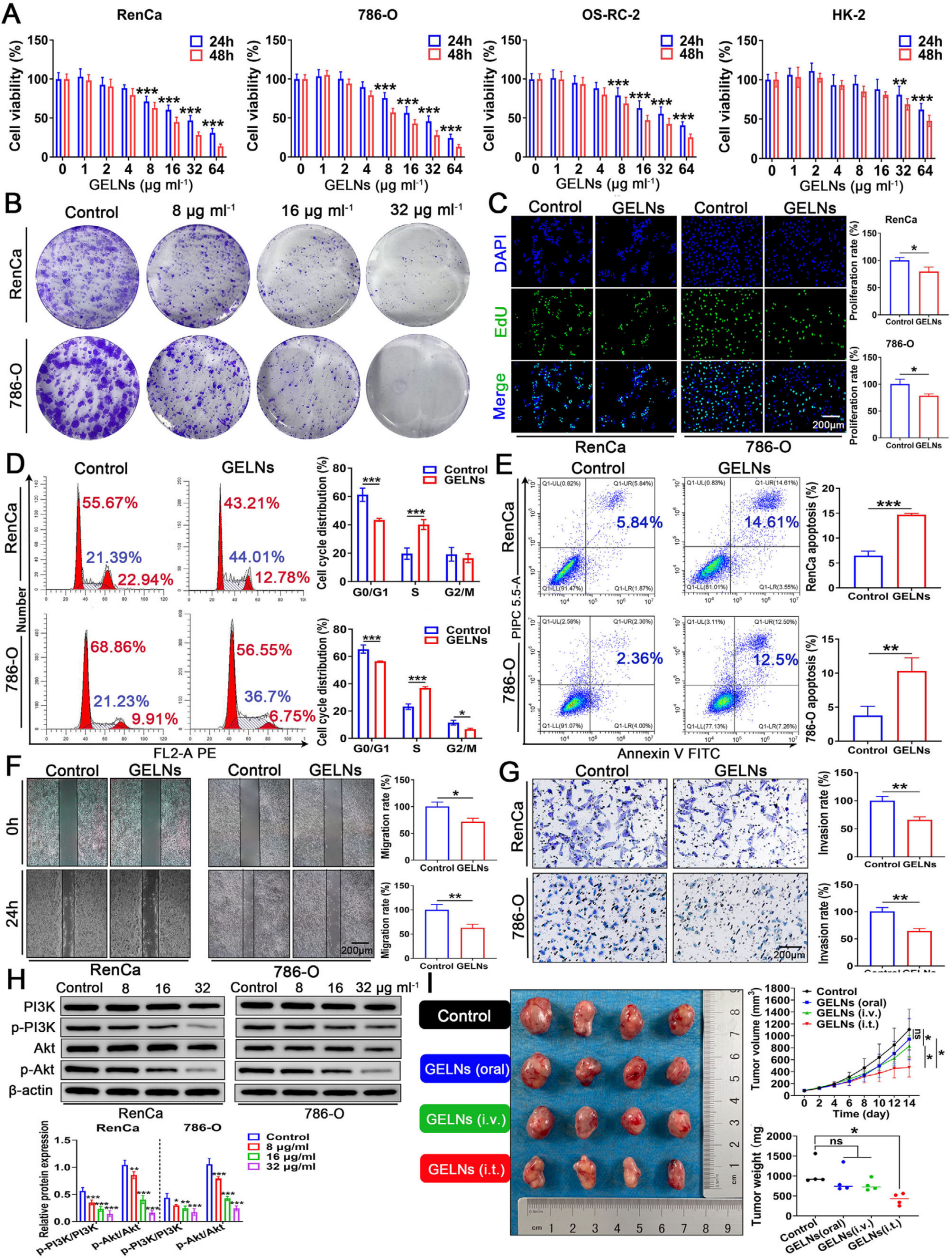
Antitumor efficacy of GELNs against renal cancer. A) Cytotoxicity assessment of RenCa, 786‐O, OS‐RC‐2, and HK2 cells after coincubation with GELNs at concentrations ranging from 1 to 64 µg mL^−1^ for 24 and 48 h. Each data point represents mean ± SD (n = 6 independent experiments). B) Effects of varying concentrations of GELNs on colony formation in RenCa and 786‐O cell lines. Impact of 8 µg mL^−1^ GELNs on the C) proliferation, D) cell cycle distribution, E) apoptosis, F) migration, and G) invasion of RenCa and 786‐O cells (n = 3 independent experiments). H) Western blot analysis of PI3K, p‐PI3K, Akt, and p‐Akt protein expression in RenCa and 786‐O cells treated with different concentrations of GELNs (n = 3 independent experiments). I) End‐point tumor images, tumor weights, and tumor growth curves of RenCa tumors following treatment with GELNs administered orally, i.v., or i.t (n = 4 mice each group). Data are presented as mean ± SD. ^*^ *p* < 0.05, ^**^ *p* < 0.01, ^***^ *p* < 0.001. ns, not significant (one‐way or two‐way ANOVA with Tukey's multiple comparison test or unpaired Student's *t* test). Scale bar = 200 µm.

Given that GELNs contain multiple pharmacological compounds with potential anticancer activities, their impact on the viability of RCC cells was assessed via an MTT assay. The GELNs demonstrated significant dose‐dependent and time‐dependent anticancer effects on RenCa, 786‐O, and OS‐RC‐2 cells (**Figure** **5**A). Notably, a concentration of 8 µg mL^−1^ was required to induce significant differences in these three cell lines, whereas the effect on HK‐2 cells remained minimal until a concentration of 32 µg mL^−1^ was reached, suggesting favorable biocompatibility. The 24 h IC50 values for RenCa, 786‐O, OS‐RC‐2, and HK‐2 cells were 26.29, 23.7, 37.47, and 95.25 µg mL^−1^, respectively (Table S8, Supporting Information). Interestingly, a low dose of GELNs (1 µg mL^−1^) slightly promoted proliferation, likely because the intrinsic proliferative capacity of the cells surpassed the inhibitory effect of the low GELN concentration. Given that the IC50 value for OS‐RC‐2 cells at 24 h was greater than that for RenCa and 786‐O cells, we selected RenCa and 786‐O cells for subsequent experiments. Clonogenic assays confirmed that GELNs inhibited tumor cell colony formation in a dose‐dependent manner (Figure 5B). Additionally, the anti‐proliferative activity of the GELNs was further validated through EdU incorporation assays (Figure 5C).

The effects of GELNs on the cell cycle and apoptosis in RenCa and 786‐O cells were evaluated via flow cytometry (FCM). GELNs arrested cells in the S phase by preventing their transition into the G2/M phase, leading to a significant accumulation of cells at this stage (Figure 5D). Consequently, these cells were unable to effectively replicate DNA and complete the processes for mitosis, including substance synthesis and energy preparation. This disruption ultimately interferes with normal cell cycle progression. In the apoptosis assay, the baseline apoptosis rates of untreated RenCa and 786‐O cells were 5.84% and 2.36%, respectively. After GELN treatment, these rates increased to 14.61% and 12.5%, respectively (Figure 5E), demonstrating the proapoptotic efficacy of the GELNs. Subsequently, scratch assays and Transwell assays were conducted to assess the impact of GELNs on cell migration and invasion. Compared with those in the control group, GELNs significantly reduced the migration rates of RenCa and 786‐O cells (Figure 5F). Additionally, the number of cells that migrated through the Matrigel‐coated membrane was markedly lower in the GELN‐treated groups than in the control groups (Figure 5G), indicating the potent antimigratory and antiinvasive properties of GELNs against RCC cells. To further elucidate the underlying anticancer mechanisms on the basis of network pharmacology results, RenCa and 786‐O cells were treated with GELNs at concentrations of 8, 16, and 32 µg mL^−1^. Western blot analysis revealed that GELNs inhibited the phosphorylation of PI3K and Akt in a dose‐dependent manner (Figure 5H). These findings suggest that the anticancer effects of GELNs may be primarily mediated through the inhibition of the PI3K‐Akt signaling pathway.

Throughout the entire treatment window in the subcutaneous renal carcinoma mouse model, the GELNs (oral) group did not demonstrate significant anticancer efficacy. This lack of efficacy may be attributed to depletion of GELNs within the gastrointestinal tract. Although the GELNs (i.v.) group delayed RenCa tumor growth to some extent, their inhibitory effect was markedly weaker than that of the intratumoral injection (i.t.) group. These findings suggest that the depletion of GELNs in systemic circulation, particularly because of their phagocytic activity in major tissues and organs, limits their effective accumulation in the TME (Figure 5I).

### 2.6 Preparation and Characterization of Nanoformulations

**Figure 6:**
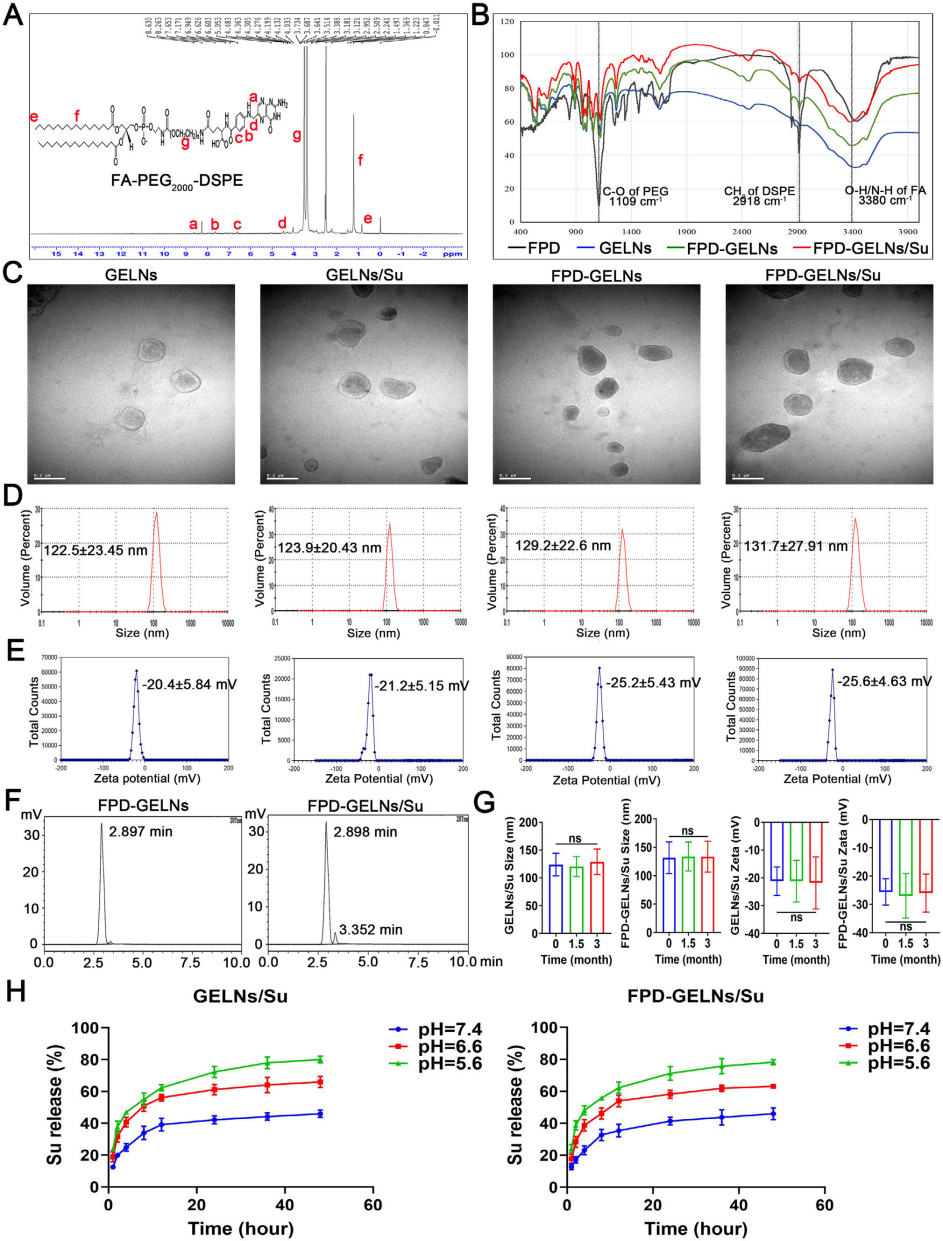
Preparation and characterization of Nanoformulations. A) ^1^H‐NMR analysis of the FA‐PEG_2000_‐DSPE structure (n = 3 independent experiments). B) FTIR spectra of different nanoformulations and FA‐PEG_2000_‐DSPE in the wavenumber range of 400–4000 cm^−1^ (n = 3 independent experiments). C) TEM images, D) DLS measurements, and E) zeta potential analysis of GELNs, GELNs/Su, FPD‐GELNs, and FPD‐GELNs/Su (n = 3 independent experiments). F) HPLC analysis of the free FPD fraction following ultrafiltration of FPD with GELNs or GELNs/Su (n = 3 independent experiments). G) Changes in particle size and zeta potential of GELNs/Su and FPD‐GELNs/Su over a three‐month period (n = 3 independent experiments). H) Su release profiles of GELNs/Su and FPD‐GELNs/Su within 48 h at various pH values (n = 3 independent experiments). Data are presented as mean ± SD. ns, not significant (one‐way or two‐way ANOVA with Tukey's multiple comparison test). Scale bar = 200 nm.

Using a UV spectrophotometer, the optimal absorption peak of Su was determined at 428 nm. On this wavelength, a concentration‐absorbance standard curve was constructed from the absorbance values measured for various concentrations of Su (Figure S6A,B, Supporting Information). This standard curve facilitated the determination of encapsulation efficiency (EE) and drug loading (DL) under different GELN‐to‐Su ratios. The DL of Su increased with increasing Su mass. When the ratio of GELNs to Su reached 20:3 (GELNs: 10 mg, Su: 1.5 mg), the DL reached a plateau phase (Table S9, Supporting Information). Therefore, this ratio was selected for subsequent experiments. The successful synthesis of the conjugate 1,2‐Distearoyl‐sn‐glycero‐3‐phosphoethanolamine‐N‐[folate (polyethylene glycol)‐2000] ‌(FA‐PEG_2000_‐DSPE, FPD) was confirmed by nuclear magnetic resonance spectroscopy of hydrogen‐1 (^1^H‐NMR) and fourier‐transform infrared (FTIR) spectroscopy. The ^1^H‐NMR spectrum of FPD exhibited characteristic peaks of FA at 8.63 ppm (a), 7.653 ppm (b), 6.603 ppm (c), and 4.483 ppm (d), PEG at 3.514 ppm (e), and DSPE at 1.223 ppm (f) and 0.847 ppm (g) (**Figure** **6**A). FTIR spectra revealed that FPD‐modified GELNs (FPD‐GELNs) and FPD‐modified Su‐loaded GELNs (FPD‐GELNs/Su) displayed characteristic vibrations of the FPD conjugate relative to those of GELNs, primarily the C‐O stretching of PEG at 1109 cm^−1^ and the CH_2_ stretching of DSPE at 2918 cm^−1^ (Figure 6B). Additionally, since the characteristic peaks of FA in the ^1^H‐NMR spectrum were less prominent than those of PEG or DSPE, it indicated that FA constituted a relatively small proportion of the functional groups in FPD (Figure 6A). Consequently, no significant C═C characteristic peaks of FA were observed in the FTIR spectra of FPD‐GELNs and FPD‐GELNs/Su, and only a slight O─H/N─H stretching vibration was detected at 3380 cm^−1^. Furthermore, the overall difference in absorbance between FPD‐GELNs and FPD‐GELNs/Su indirectly suggested the successful loading of Su (Figure 6B).

TEM images revealed that the GELNs, GELNs/Su, FPD‐GELNs, and FPD‐GELNs/Su exhibited typical spherical morphologies (Figure 6C). Following FPD modification, the particle size of GELNs was observed to be marginally larger than that of the unmodified GELNs. This increase in particle size can be attributed to the ligand coating, which slightly enlarged the particles. In contrast, the encapsulation of Su had a negligible effect on the particle size of the GELNs (Figure 6D). The inherent negative charges of FA and DSPE within FPD caused the nanoparticles to exhibit a more negative zeta potential, whereas the encapsulation of Su did not significantly affect the zeta potential of the GELNs (Figure 6E). The polydispersity index (PDI) values for all four nanoformulations were less than 0.3, indicating their uniform particle size distribution. The detailed characterization data are provided (Table S10, Supporting Information). To evaluate the conjugation efficiency of FPD, the maximum absorption wavelength of FPD was determined as 287 nm using a UV spectrophotometer (Figure S6C, Supporting Information). A calibration curve correlating FPD concentration with peak area was established using HPLC at a UV detection wavelength of 287 nm (Figure S6D, Supporting Information). FPD was conjugated with GELNs or GELNs/Su at a 1:1 mass ratio, and the conjugation efficiencies were calculated based on peak area integration, yielding values of 58.19% and 58.93%, respectively (Figure 6F). Over a period of 3 months, the particle size, zeta potential, PDI, and DL of GELNs/Su and FPD‐GELNs/Su remained stable with no significant changes (Figure 6G; Table S11, Supporting Information), demonstrating their long‐term storage stability.

### 2.7 A Mildly Acidic Environment Facilitated the Release of Su

At a pH of 7.4, the sustained and gradual release of Su from GELNs/Su and FPD‐GELNs/Su was observed, reaching a plateau at 24 h (Figure 6H). Within the initial 24 h period, only 42.11 ± 2.51% and 41.36 ± 2.32% of the Su was released from the GELNs/Su and FPD‐GELNs/Su, respectively, with the cumulative release not exceeding 45% by 48 h. Compared with that at pH 6.6, the release of Su was significantly greater at pH 5.6. The total percentages of Su released from GELNs/Su and FPD‐GELNs/Su at 48 h were 80.08 ± 2.12% and 78.25 ± 1.62%, respectively (Figure 6H). These findings indicate that an acidic environment accelerates the drug release rate from the nano‐delivery systems, indicating that the slightly acidic TME is beneficial for drug delivery.

### 2.8 The FOLR Expression Profile in Tumor Cells and Macrophages

**Figure 7:**
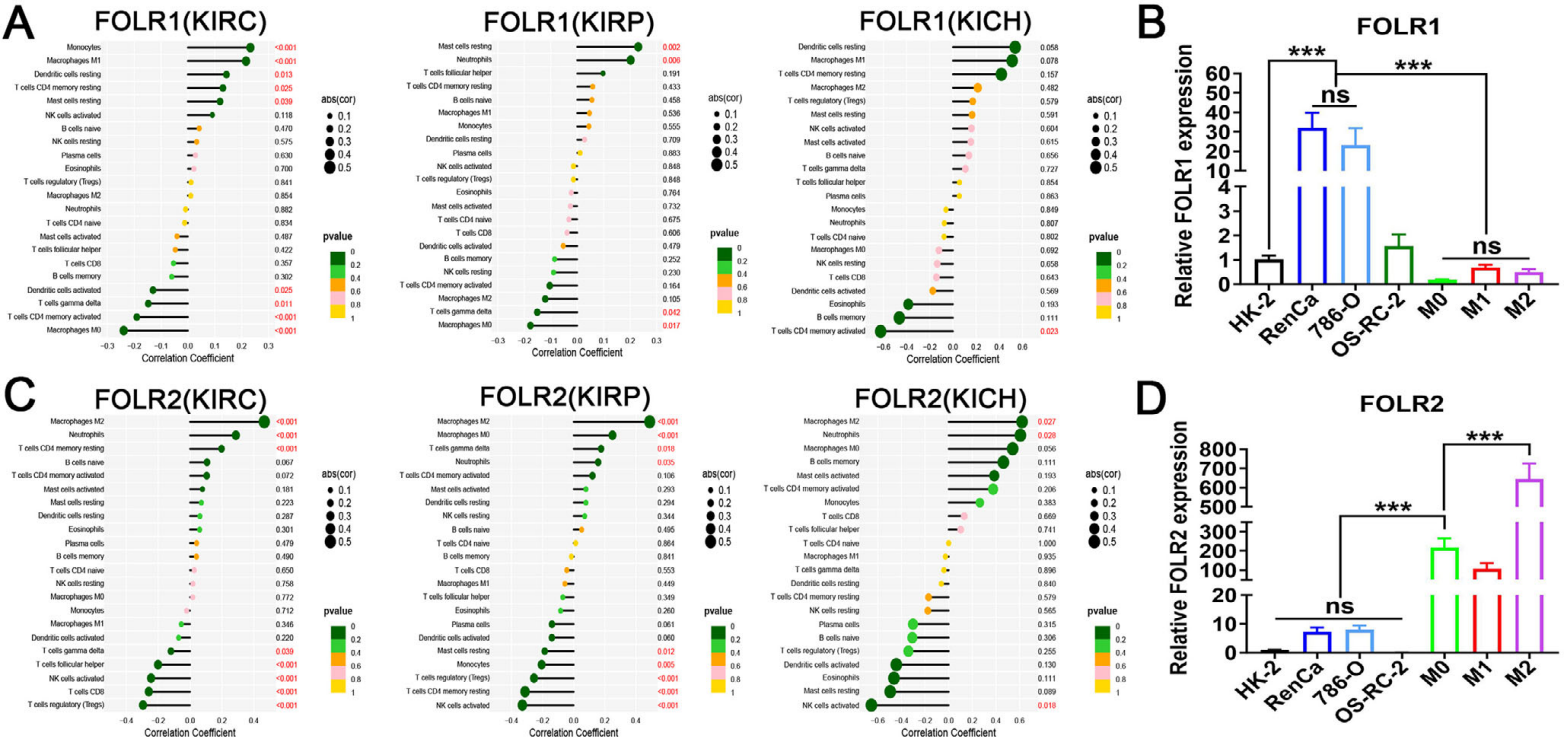
Bioinformatics and RT‐qPCR analysis of FOLR1 and FOLR2. Correlation analysis between A) FOLR1, C) FOLR2 expression, and immune cell infiltration in the TCGA‐KIRC, TCGA‐KIRP, and TCGA‐KICH cohorts. Relative mRNA expression levels of B) FOLR1 and D) FOLR2 in various cell types (n = 3 independent experiments). Data are presented as mean ± SD. ^***^ *p* < 0.001. ns, not significant (one‐way ANOVA with Tukey's multiple comparison test).

**Figure 8:**
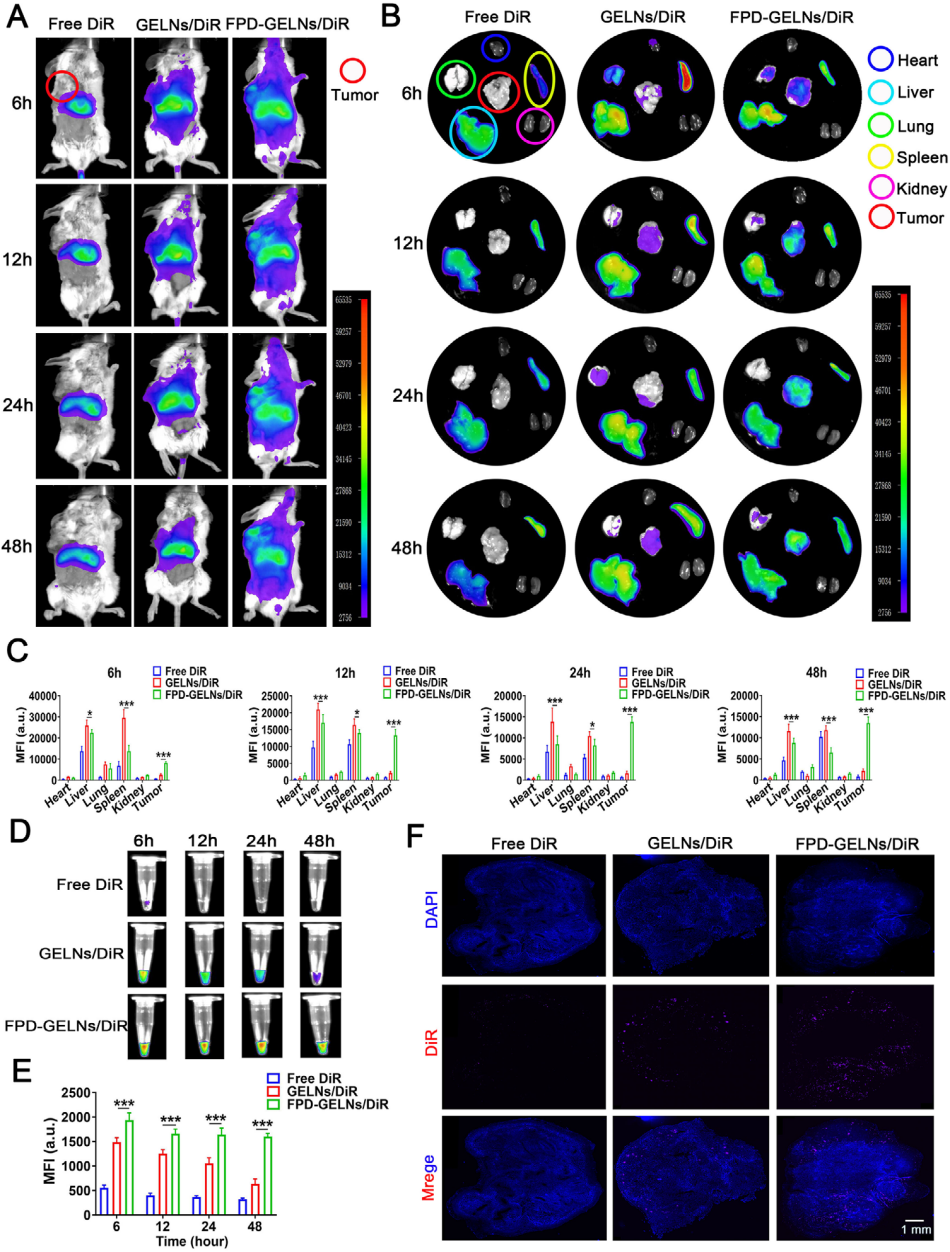
In vivo tumor‐targeting evaluation of FPD. Fluorescence images of A) whole‐body B, C) major organs (heart, liver, lung, spleen, kidney, and tumor), and D, E) serum of RenCa subcutaneous tumor‐bearing mice at different time points after a single administration of free DiR, GELNs/DiR, and FPD‐GELNs/DiR (n = 3 mice each group). F) Fluorescence images showing DiR accumulation in RenCa tumor sections following a single administration of free DiR, GELNs/DiR, and FPD‐GELNs/DiR (n = 3 independent experiments). Data are presented as mean ± SD. ^*^ *p* < 0.05, ^***^ *p* < 0.001. ns, not significant (two‐way ANOVA with Tukey's multiple comparison test). Scale bar = 1 mm.

The TCGA‐KIRC, TCGA‐KIRP, and TCGA‐KICH cohorts were utilized to assess the correlation between FOLR expression and immune cell subtypes. As shown in **Figure** **7**A, in ccRCC, chRCC, and pRCC, M0 and M2 macrophages did not correlate with FOLR1 expression. Furthermore, FOLR1 mRNA levels were markedly higher in RenCa and 786‐O cells than in HK‐2 cells and macrophages, suggesting that FOLR1 is predominantly expressed in tumor cells (Figure 7B and *p* < 0.001). Conversely, M2 macrophages presented the highest positive correlation with FOLR2 expression among all immune cells in ccRCC, pRCC, and chRCC, whereas M0 macrophages also presented positive correlations across all RCC subtypes, although some correlations lacked statistical significance (Figure 7C; Figure S7A–C, Supporting Information). Finally, FOLR2 mRNA expression was significantly elevated in M0 and M2 macrophages relative to that in tumor cells and normal tissue cells, with M2 macrophages showing higher expression than M0 macrophages, indicating that M2 macrophages may possess enhanced specificity for targeting FOLR2 relative to M0 macrophages (**Figure** **8**D and *p* < 0.001).

### 2.9 Bioavailability in the Body

Owing to the potential instability of PELNs under pH and temperature variations in the digestive system, as well as the rapid degradation of biomolecules by the gastrointestinal tract, intravenous administration, which is characterized by high bioavailability and rapid onset of action, has been the most effective delivery method.^[^ ^15^, ^33^ ^]^ Although oral administration offers convenience and safety, it is not the most efficacious option for optimal therapeutic outcomes.^[^ ^15^, ^33^ ^]^ Therefore, in this study, the primary administration method employed was intravenous injection. As shown in Figure 8A–C, GELNs began to accumulate in the tumor at 6 h postinjection and maintained a relatively low fluorescence level throughout the 48 h period. Given that the liver and spleen are the primary organs receiving drugs after intravenous administration, their nonspecific uptake of GELNs led to significant accumulation in these organs, thereby explaining the weaker anticancer efficacy of GELNs (i.v.) than that of GELNs (i.t.) within the therapeutic window (Figure 5I). In contrast, FPD‐GELNs accumulated rapidly in the tumor at 6 h postinjection, with the accumulation amount increasing in a time‐dependent manner. The fluorescence levels at each time point during the single injection window were significantly greater than those of the GELNs, without any notable fluorescence decay. Notably, FPD modification not only significantly diminished the GELN load in major organs, particularly the liver and spleen, but also markedly prolonged the fluorescence signal of GELNs in circulation (Figure 8D,E). This observation can be ascribed to the FA‐mediated EPR effect and the extended circulation time facilitated by PEG.

The tumor tissues collected 48 h after a single injection were processed into frozen sections. Fluorescence microscopy revealed that the FPD modification resulted in a marked increase in the near‐infrared fluorescent probes (DiR) fluorescence signal from GELNs (Figure 8F). These findings are consistent with the aforementioned results.

### 2.10 FPD Significantly Enhanced the Uptake of GELNs by Tumor Cells

**Figure 9:**
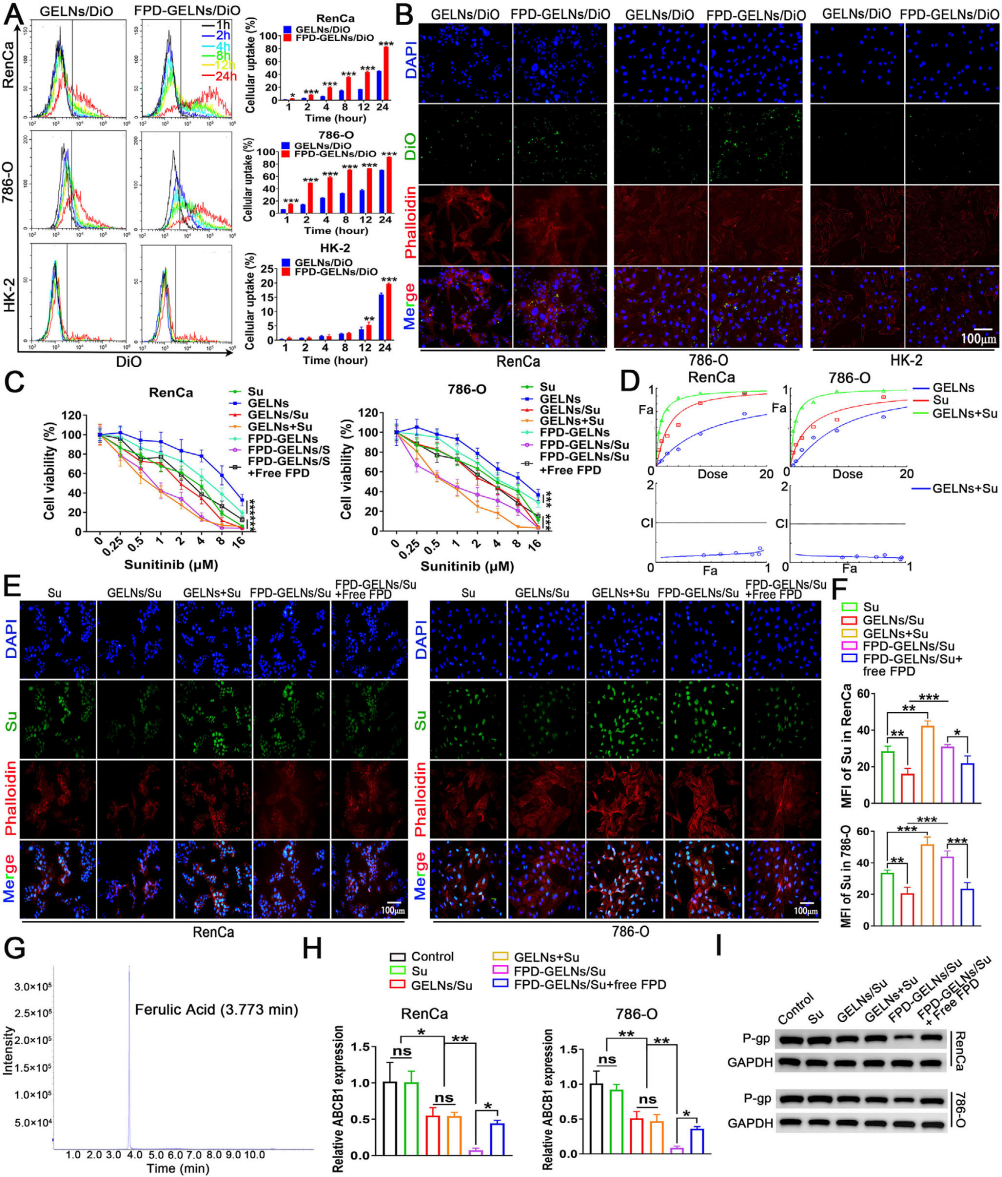
The enhancing effect of FPD on Su sensitivity mediated by GELNs targeting ABCB1/P‐gp. A) Evaluation of the uptake efficiency of GELNs/DiO and FPD‐GELNs/DiO by RenCa, 786‐O, and HK2 cells at different time points (n = 3 independent experiments). B) Fluorescence images of GELNs/DiO and FPD‐GELNs/DiO taken up by RenCa and 786‐O cells at 24 h (n = 3 independent experiments). C) Cytotoxicity assessment of different nanocarriers on RenCa and 786‐O cells treated with Su at concentrations ranging from 0.25 to 16 µм (n = 6 independent experiments). D) Combined effect curve of GELNs + Su at Su concentrations ranging from 0.5 to 16 µм. E) Intracellular Su accumulation fluorescence images and F) quantitative analysis (MFI) after 24 h treatment with various nanocarriers at 1 µм Su concentration (n = 3 independent experiments). G) Negative ion mode HPLC employed to detect Ferulic acid (n = 3 independent experiments). Relative expression analysis of H) ABCB1 mRNA and I) P‐gp protein levels in RenCa and 786‐O cells after treatment with various nanocarriers at 1 µм Su concentration (n = 3 independent experiments). Data are presented as mean ± SD. ^*^ *p* < 0.05, ^**^ *p* < 0.01, ^***^ *p* < 0.001. ns, not significant (one‐way or two‐way ANOVA with Tukey's multiple comparison test). Scale bar = 100 µm.

To investigate whether FPD enhances the uptake of GELNs by RCC cells, FCM was employed. As shown in **Figure** **9**A, the uptake of GELNs by RenCa, 786‐O, and HK‐2 cells increased in a time‐dependent manner. After 24 h, the uptake rates were 15.98 ± 0.51% for HK‐2 cells, 45.29 ± 0.25% for RenCa cells, and 70.19 ± 0.52% for 786‐O cells. Normal cell lines, such as HK‐2, may exhibit lower capture efficiency of GELNs due to specific mechanisms, leading to significantly higher IC50 values for cell viability than for tumor cells (Table S8, Supporting Information). For FPD‐GELNs, the uptake rates of RenCa and 786‐O cells at each time point were significantly greater than those of unmodified GELNs. At 24 h, the uptake rates reached 82.85 ± 0.65% for RenCa and 91.45 ± 0.61% for 786‐O cells, demonstrating that FPD modification markedly improved the uptake of GELNs by RCC cells. In contrast, owing to the relatively low expression of FOLR1 mRNA in HK‐2 cells, a significant increase in FPD‐GELNs uptake was observed only at 12 and 24 h (Figure 9A).

Based on the time‐dependent uptake curves, fluorescence microscopy was used to observe the intracellular fluorescence intensity of green fluorescent probes (DiO). FPD modification significantly increased the DiO fluorescence intensity of GELNs at 24 h (Figure 9B), which is consistent with the results presented in Figure 9A.

### 2.11 FPD Enhanced the Sensitization Effect of Su by GELNs‐targeted ABCB1/P‐gp Reduction

The in vitro cytotoxicity of various nanoformulations on RCC cells was evaluated via the MTT assay. Both GELNs and Su inhibited RCC cell viability in a concentration‐dependent manner. Compared with the GELNs alone, the GELNs/Su group demonstrated enhanced cytotoxicity, as the GELNs not only exerted cytotoxic effects through their inherent pharmacological molecules but also facilitated the intracellular drug release of Su, thereby inhibiting RCC cell progression (Figure 9C). Furthermore, the combination effect curves indicated that the GELNs/Su group enhanced the synergistic interaction between the GELNs group and the Su group (Figure S8A, Supporting Information; predominantly synergism, combination index (CI): 0.3–0.7). In contrast, the GELNs + Su group presented a significantly increased therapeutic effect (Figure 9D; predominantly strong synergism, CI: 0.1–0.3), likely because GELNs increased the intracellular accumulation of Su (Figure 9E,F). The significantly lower CI value for the GELNs + Su group than for the GELNs/Su group can be attributed to the fact that the intracellular uptake of Su in the GELNs/Su formulation is not dependent on the rapid diffusion of free Su, but rather on the amount of GELNs/Su complexes that enter the cells. Despite carrying only a portion of Su into cells, the GELNs/Su group still exhibited greater cytotoxicity than the Su group alone, indicating significant sensitization of Su by GELNs (Figure 9C–F).

Modification with FPD significantly increased the cytotoxicity of both the GELNs and the GELNs/Su (Figure 9C). Concurrently, the intracellular accumulation of Su in GELNs/Su was markedly increased due to the binding of FPD (Figure 9E,F). However, the enhanced cytotoxicity and intracellular Su accumulation observed in the FPD‐GELNs/Su group were inhibited by excess free FPD, indicating that the FA/FOLR1 ligand‐receptor interaction mediates the effective cytotoxicity and intracellular delivery of FPD‐GELNs/Su (Figure 9C,E,F). Furthermore, FPD‐GELNs/Su significantly amplified the therapeutic efficacy of both the FPD‐GELNs and Su groups (Figure S8B, Supporting Information; predominantly strong synergism, CI: 0.1–0.3), demonstrating a synergistic effect superior to that of the GELNs/Su group. There fundings further underscore the critical role of the FA/FOLR1 ligand‐receptor structure in this biological process. The CI values and corresponding drug synergy evaluation criteria are provided (Table S12, Supporting Information).

As a key member of the ATP‐binding cassette (ABC) transporter family, P‐gp (ABCB1) plays a critical role in multidrug resistance (MDR) in cancer cells.^[^ ^34^, ^35^ ^]^ Su is a substrate of P‐gp, and its efflux efficiency is closely associated with the inhibition or activation of P‐gp expression and function.^[^ ^36^, ^37^ ^]^ Notably, our analysis of the drug regulatory networks in human RCC (Figure 2A) and mouse RCC (Figure S3A, Supporting Information) revealed that ABCB1 was a target of GELNs. Eleven compounds, including 4‐hydroxycinnamoylagmatine, 9,12‐octadecadiynoic acid, and Ferulic acid, may exert synergistic regulatory effects on ABCB1. Molecular docking studies demonstrated that all 11 compounds successfully docked with ABCB1 through various intermolecular interactions (Figure S9A, Supporting Information). The positions and grid sizes of the active pocket are detailed (Table S6, Supporting Information), and the average binding energy of these compounds was 6.36 kcal mol^−1^ (Table S7, Supporting Information), indicating their potential to influence ABCB1 expression or function. Given that Ferulic acid has been previously reported to modulate ABCB1 expression,^[^ ^38^ ^]^ we quantified its content in GELNs using a standard calibration curve to validate our hypothesis (Figure S4, Supporting Information). The concentration of Ferulic acid in ginger juice was 79.65 ng mL^−1^, whereas in GELNs it was slightly higher at 89.72 ng mL^−1^ (Figure 9G; Figure S9B, Supporting Information). Next, RT‐qPCR and Western blot analyses were conducted to assess the impact of each treatment group on ABCB1/P‐gp expression. FPD‐GELNs had a more pronounced inhibitory effect on the mRNA and protein levels of ABCB1/P‐gp than did GELNs alone. This effect was abrogated by excess free FPD, suggesting that the FA/FOLR1 ligand‐receptor interaction mediates the reduction in ABCB1/P‐gp expression targeted by GELNs, thereby increasing the sensitivity to Su (Figure 9H,I).

### 2.12 Ex Vivo Macrophage‐Targeting Efficacy of FPD

**Figure 10:**
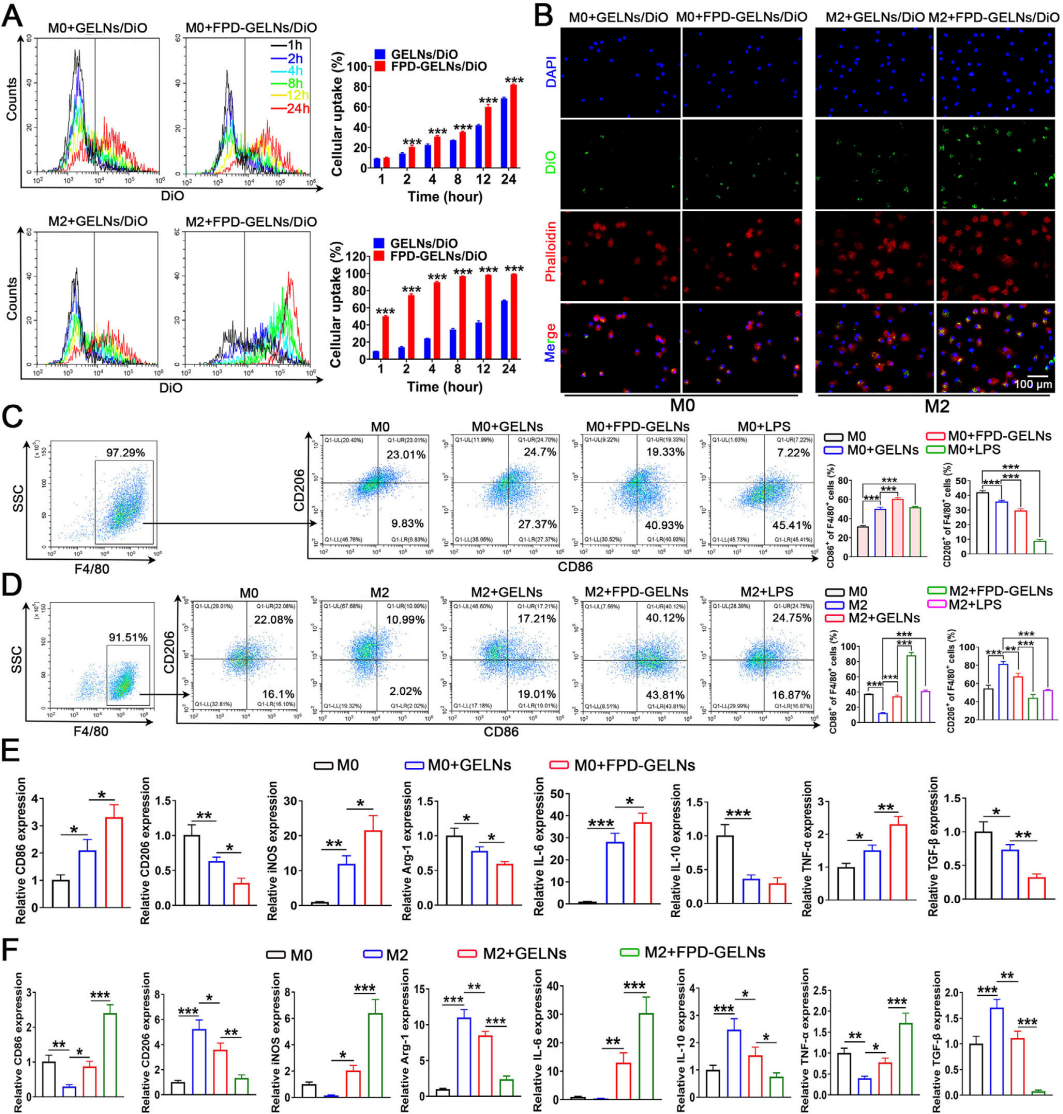
In vitro macrophage targeting by FPD and its ability to enhance GELN‐induced macrophage phenotype polarization. A) Evaluation of the uptake efficiency of GELNs/DiO and FPD‐GELNs/DiO by M0 and M2 macrophages at different time points (n = 3 independent experiments). B) Fluorescence images of GELNs/DiO and FPD‐GELNs/DiO taken up by M0 and M2 macrophages at 24 h (n = 3 independent experiments). FCM analysis of polarization response from C) M0 to M1 and D) M2 to M1 induced by GELNs, FPD‐GELNs, and LPS (n = 3 independent experiments). Phenotypic changes in polarization markers of E) M0 and F) M2 macrophages (M1: CD86, iNOS, IL‐6, TNF‐α; M2: CD206, Arg‐1, IL‐10, TGF‐β) following intervention with GELNs and FPD‐GELNs (n = 3 independent experiments). Data are presented as mean ± SD. ^*^ *p* < 0.05, ^**^ *p* < 0.01, ^***^ *p* < 0.001. ns, not significant (one‐way or two‐way ANOVA with Tukey's multiple comparison test). Scale bar = 100 µm.

To investigate the effect of FPD on the in vitro uptake of GELNs by M0 and M2 macrophages, FCM was employed. Both M0 and M2 macrophages internalized GELNs in a time‐dependent manner, achieving 24 h capture efficiencies of 68.45 ± 1.04% and 68.24 ± 0.58%, respectively. Notably, FPD modification significantly enhanced the uptake efficiency of GELNs, with 24 h capture efficiencies increasing to 82.06 ± 0.68% for M0 and 99.33 ± 0.17% for M2 (**Figure** **10**A). Given that FOLR2 mRNA expression was markedly greater in M2 than in M0 (Figure 7D), the superior enhancement of GELN uptake by FPD in M2 can be attributed to this differential expression. The fluorescence images presented confirmed these findings, showing the uptake of GELNs/DiO and FPD‐GELNs/DiO by M0 and M2 at 24 h (Figure 10B), which aligned with the quantitative data.

The transformation of macrophages from the tumor‐promoting M2 phenotype to the immunostimulatory M1 phenotype, which releases antitumor factors, is considered a promising strategy for cancer immunotherapy.^[^ ^39^ ^]^ Therefore, we evaluated the effects of GELNs on macrophage polarization in vitro. The FCM results demonstrated that GELNs significantly induced the polarization of M0 macrophages toward the M1 phenotype (F4/80^+^CD86^+^), and FPD modification markedly accelerated this process (Figure 10C). The M2 phenotype (F4/80^+^CD206^+^) typically promotes tumor growth and treatment resistance in the TME by creating an immunosuppressive state.^[^ ^40^ ^]^ To investigate whether GELNs can reverse the M2 phenotype to the M1 phenotype, we induced M2 polarization via interleukin‐4 (IL‐4). IL‐4 shifted the phenotypic landscape of M0 macrophages, increasing their polarization toward the F4/80^+^CD206^+^ M2 phenotype and confirming successful M2 induction (Figure 10D). The addition of GELNs subsequently significantly increased the proportion of CD86^+^ cells while decreasing the percentage of CD206^+^ cells, promoting the reprogramming of M2 macrophages toward the M1 phenotype. FPD loading further enhanced this polarization effect (Figure 10D). RT‐qPCR analysis revealed consistent transcriptional changes with the FCM data (Figure 10E,F), indicating that FPD significantly promoted the functional reprogramming of GELNs by upregulating the expression of M1‐related markers and downregulating the expression of M2‐related genes.

### 2.13 The Immune Mechanism Underlying FPD‐Enhanced GELN‐Mediated M1 Macrophage Polarization

**Figure 11:**
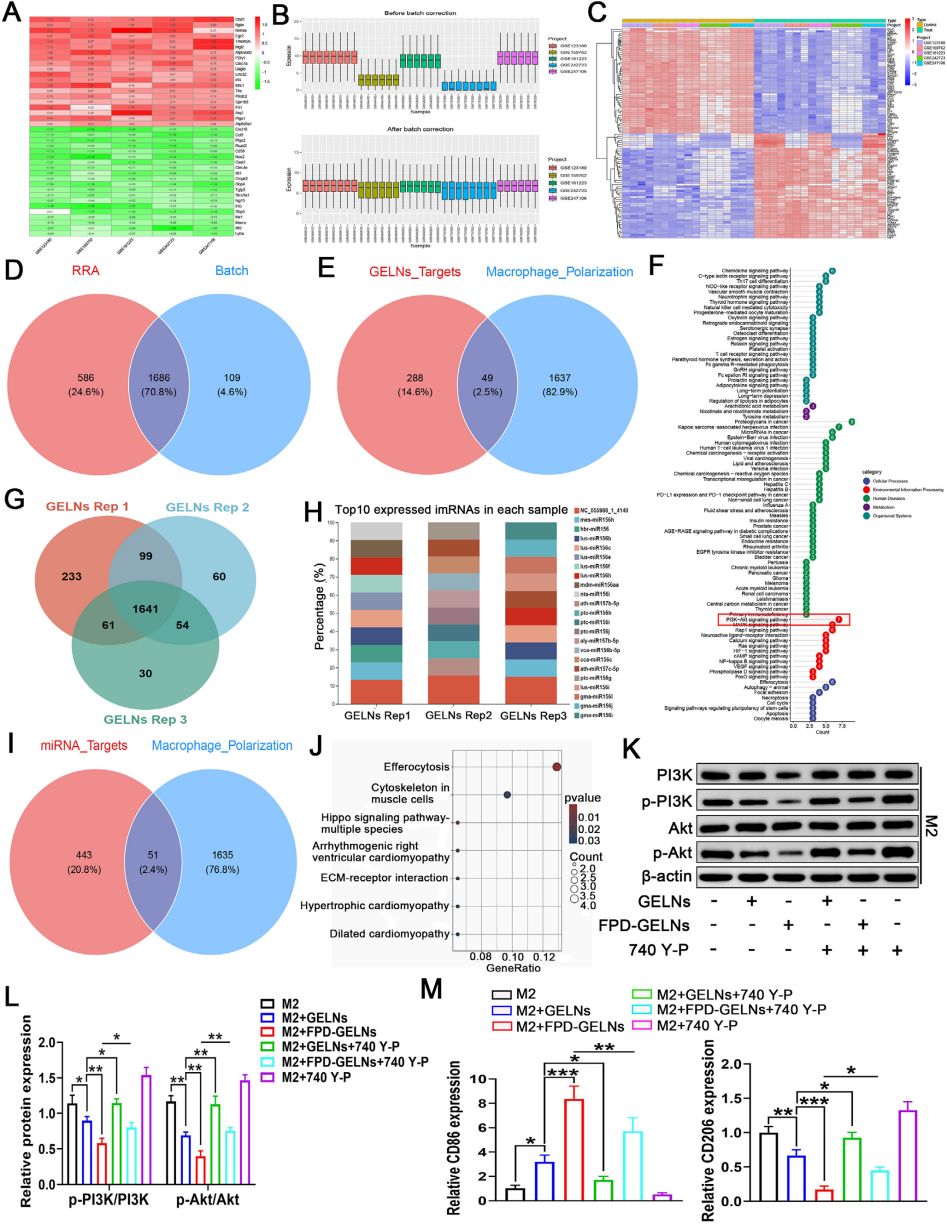
Investigation of the immune mechanisms underlying M1 macrophage polarization induced by GELNs and FPD‐GELNs. A) Heatmap of RRA analysis, B) SVA‐based batch correction, and C) heatmap of differential expression analysis after batch correction for five mRNA datasets. D) Venn diagram showing overlapping DEGs identified by RRA and batch‐corrected methods. E) Venn diagram of the intersection between GELN metabolite targets and macrophage polarization‐associated targets. F) KEGG analysis of characteristic genes involved in GELN metabolite‐mediated M2‐to‐M1 macrophage polarization. G) Venn diagram of miRNA sequencing results from three independent GELN samples and H) heatmap of the top 10 most highly expressed miRNAs (n = 3 independent experiments). I) Venn diagram of overlapping targets between GELN miRNAs and macrophage polarization‐related genes. J) KEGG analysis of characteristic genes associated with miRNA‐regulated M2‐to‐M1 polarization in GELNs. K) Western blot images and L) quantitative statistical analysis of PI3K‐Akt signaling pathway protein expression across different treatment groups (n = 3 independent experiments). M) Relative mRNA expression levels of CD86 and N) CD206 measured by RT‐qPCR under various treatment conditions (n = 3 independent experiments). Each data point represents mean ± SD. ^*^ *p* < 0.05, ^**^ *p* < 0.01, ^***^ *p* < 0.001. ns, not significant (one‐way or two‐way ANOVA with Tukey's multiple comparison test).

To investigate the specific components mediating immune regulation in GELNs, we first retrieved multiple mRNA microarray datasets of M1 and M2 macrophages from the GEO database. Using the robust rank aggregation (RRA) combined with batch effect correction (Batch method), we identified 1686 characteristic genes associated with the polarization of M2 toward the M1 phenotype (**Figure** **11**A–D). By intersecting these genes with 337 murine target genes of GELN metabolites (Figure S2D, Supporting Information), we obtained 49 key overlapping targets potentially involved in regulating M2‐to‐M1 polarization (Figure 11E). As detailed in Table S13 (Supporting Information), the top 10 compounds targeting these polarization‐related genes include Cadabicine methyl ether, Frangulanine, and 6‐Gingerdione, which may play pivotal roles in immune modulation. KEGG analysis revealed that the PI3K‐Akt signaling pathway was the most significantly enriched within the “Environmental Information Processing” category (Figure 11F). Next, small RNA sequencing was performed on GELNs, revealing 1641 commonly expressed miRNAs across three replicate samples (Figure 11G). The top 10 miRNAs with the highest relative expression levels are presented in Figure 11H. Target prediction using the psRobot database, followed by integration with macrophage polarization signature genes, identified 51 potential miRNA‐mRNA regulatory targets involved in polarization (Figure 11I). Table S14 (Supporting Information) lists the top 10 miRNAs based on their predicted targeting of polarization‐associated genes, Notably, multiple members of the mes‐miR477 and gma‐miR164 families exhibit the greatest target connectivity, suggesting their potential involvement in critical immune regulatory mechanisms. KEGG analysis further indicated that GELNs‐derived miRNAs may contribute to macrophage immune remodeling through the efferocytosis pathway (Figure 11J). This finding is consistent with previous reports highlighting the critical role of efferocytosis in orchestrating macrophage‐mediated immune reprogramming within TME.^[^ ^41^ ^]^ Furthermore, proteomic profiling of GELNs identified a total of 1290 proteins (Figure S10A, Supporting Information). Subcellular localization analysis indicated that the majority of these proteins were predominantly localized in the chloroplast and cytoplasm (Figure S10B, Supporting Information). Functional enrichment via GO and KEGG analyses demonstrated that these proteins are primarily involved in signal transduction and diverse metabolic processes (Figure S10C,D, Supporting Information). Collectively, these multi‐omics findings reinforce the potential role of GELNs in modulating immune responses within the TME.

Given that the pharmacological metabolites of GELNs likely mediate macrophage remodeling predominantly via the PI3K‐Akt signaling pathway (Figure 11F), we investigated this mechanism using Western blot analysis and found that FPD encapsulation significantly enhanced the inhibitory effect of GELNs on PI3K‐Akt phosphorylation in M2 macrophages. This effect was markedly reversed upon supplementation with the PI3K activator 740 Y‐P (Figure 11K,L). To further validate the critical role of the PI3K‐Akt pathway in macrophage remodeling, RT‐qPCR analysis revealed that 740 Y‐P attenuated the upregulation of CD86 and the downregulation of CD206 induced by both GELNs and FPD‐GELNs (Figure 11M). These findings demonstrate that GELNs promote M2‐to‐M1 macrophage polarization via modulation of the PI3K‐Akt signaling pathway, and the incorporation of FPD potentiates this effect.

### 2.14 In Vivo Macrophage‐Targeting Efficacy of FPD and the Nanoformulations‐Mediated Immuno‐Reprogramming Effects

To evaluate the in vivo targeting efficacy of FPD to TAMs, we analyzed single‐cell suspensions of solid tumors via FCM. After a single administration of FPD‐GELNs/DiR, the TAMs (F4/80^+^CD11b^+^) in the TME were significantly greater than those in the GELNs/DiR group (Figure S11A, Supporting Information), demonstrating that FPD loading enhanced the in vivo targeting efficiency of GELNs/DiR toward TAMs. Notably, a similar trend was observed for MDSCs in the TME (Figure S11B, Supporting Information), which may be attributed to the high expression of FOLR2 in certain tumor‐associated myeloid cells.^[^ ^24^ ^]^ These findings demonstrate how FPD‐GELNs modulate immune‐suppressive cell subsets within the TME.

**Figure 12:**
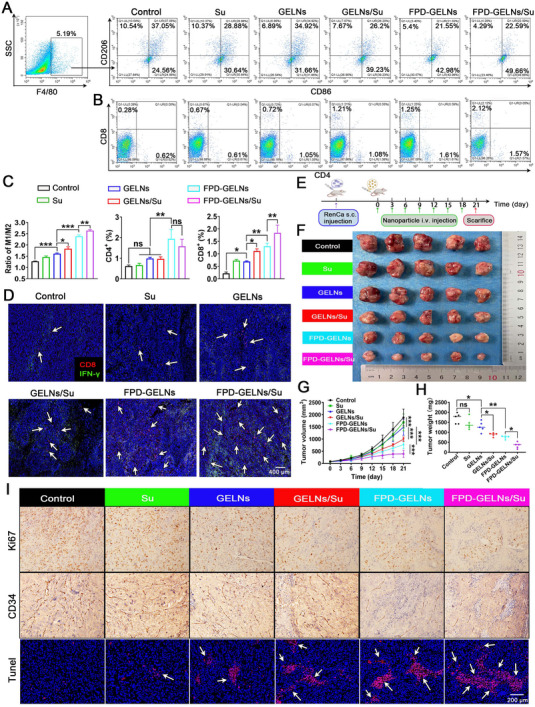
In vivo anticancer efficacy and immune reprogramming effects of nanoformulated therapeutics. FCM of tumor single‐cell suspensions from various nanoformulation treatment groups was performed to determine the proportions of A, C) M1 (F4/80^+^CD86^+^) and M2 (F4/80^+^CD206^+^) macrophages, B, C) CD4^+^ T and CD8^+^ T cells. (n = 3 independent experiments). D) IF images depicting CD8^+^ and IFN‐γ expression (n = 3 independent experiments, Scale bar = 400 µm). E) Schematic representation of the intervention time points for nanoformulations in subcutaneous RenCa tumors in mice. F) Tumor images and H) tumor weight statistics from different treatment groups at the conclusion of the experiment (n = 5 mice each group). G) Growth curves of RenCa tumors across various treatment groups (n = 5 mice each group). I) IHC for Ki67 and CD34, and TUNEL staining of RenCa tumor sections from different treatment groups (n = 3 independent experiments). Each data point represents mean ± SD. ^*^ *p* < 0.05, ^**^ *p* < 0.01, ^***^ *p* < 0.001. ns, not significant (one‐way or two‐way ANOVA with Tukey's multiple comparison test). Scale bar = 200 µm.

The relative abundances of tumor‐infiltrating immune cell populations, including TAMs and T lymphocyte subsets, were subsequently assessed via FCM. Consistent with our expectations, GELN or GELN/Su intervention significantly increased the M1/M2 ratio, indicating effective reprogramming of TAMs. This reprogramming efficacy was further enhanced by FPD‐GELNs or FPD‐GELNs/Su (**Figure** **12**A,C), which mirrored the in vitro induction results observed in BMDMs (Figure 10C–F). Additionally, the incorporation of FPD and encapsulation of Su resulted in increased infiltration of CD4^+^ and CD8^+^ T cells (Figure 12B,C). Immunofluorescence (IF) demonstrated that FPD‐GELNs and FPD‐GELNs/Su exhibited the highest levels of CD8+ T cell abundance and IFN‐γ expression relative to other treatment groups (Figure 12D). These findings indicate that the synergistic integration of FPD with GELNs or GELNs/Su enhances the adaptive immune response elicited by these nanocarriers.

The in vivo anti‐cancer efficacy of the nanoformulations was evaluated in a RenCa subcutaneous tumor model in BALB/c mice. When the subcutaneous tumors reached ≈50 mm^3^, treatment interventions were administered every 3 days for a total of 7 injections (Figure 12E). The results demonstrated that Su and GELNs alone exhibited limited anticancer efficacy. Although GELNs/Su enhanced the tumor suppression effect, the combination of FPD with GELNs/Su achieved the most significant inhibition of renal cancer progression. This superior efficacy can be attributed to the efficient targeting capability of FPD and the effective drug delivery properties of Su, resulting in satisfactory therapeutic outcomes (Figure 12F–H).

To investigate the mechanisms underlying the in vivo anticancer effects of the nanoformulations, histological analyses were conducted on subcutaneous tumors from euthanized mice. These analyses included Ki67 (a proliferation marker), CD34 (a vascularization marker), and TUNEL (an apoptosis marker) (Figure 12I). Microvessel density within the tumor showed slight changes in the Su and GELNs groups. However, after the GELN/Su and FPD‐GELN treatments, the microvessel density significantly decreased, with the most substantial reduction observed in the FPD‐GELNs/Su group. Similarly, FPD‐GELNs/Su exhibited the most pronounced anti‐proliferative and pro‐apoptotic effects compared to all other treatment groups (Figure 12I).

### 2.15 Pharmacokinetic Evaluation of Su

**Figure 13:**
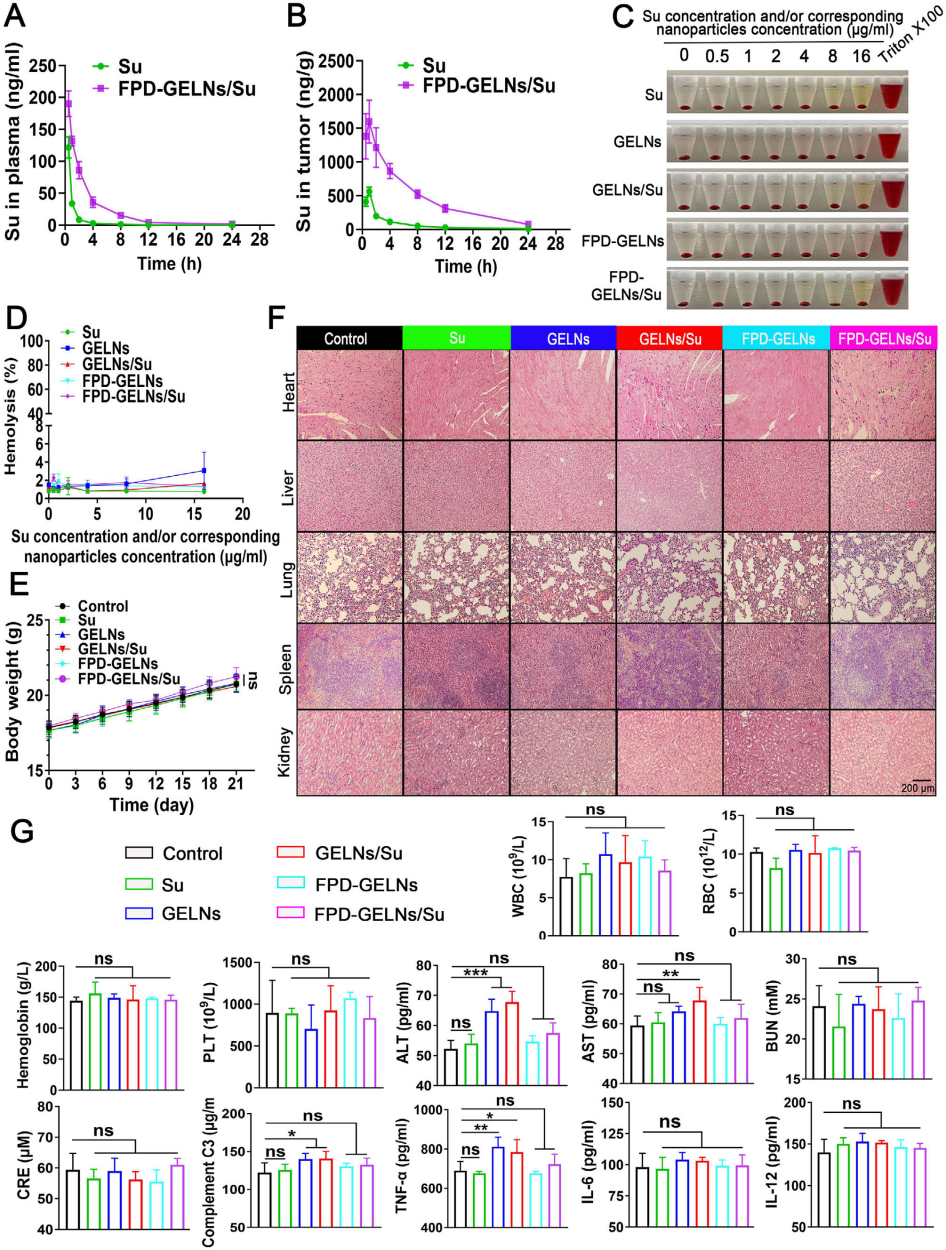
Comprehensive pharmacokinetic evaluation and biosafety assessment of the Su‐loaded nanoformulation. A) Plasma and B) tumor time‐concentration profiles of Su in RenCa tumor‐bearing mice (n = 3 mice each group). C) Schematic representation of hemolysis phenomena and D) statistical analysis of hemolysis rates for different nanoformulations at Su concentrations ranging from 0.5 to 16 µg mL^−1^ (n = 3 independent experiments). E) Weight change curves of the mice across various treatment groups throughout the therapeutic window (n = 5 mice each group). F) H&E staining of tissue sections from major organs (heart, liver, lung, spleen, and kidney) in different treatment groups (n = 3 independent experiments). G) Post‐treatment routine blood analysis of WBC, RBC, hemoglobin, and PLT levels in mice from different nanoformulation intervention groups, alongside ELISA analysis of blood biochemical indicators, including ALT, AST, BUN, CRE, and inflammatory factors, such as complement C3, TNF‐α, IL‐6, and IL‐12 (n = 5 mice each group). Data are presented as mean ± SD. ^*^ *p* < 0.05, ^**^ *p* < 0.01, ^***^ *p* < 0.001. ns, not significant (one‐way or two‐way ANOVA with Tukey's multiple comparison test). Scale bar = 200 µm.

To investigate whether FPD‐GELNs facilitate enhanced delivery of Su into tumor tissues, the pharmacokinetic profiles of FPD‐GELNs/Su and free Su were evaluated in both plasma and tumor tissue. As shown in **Figure** **13**A and Table S15 (Supporting Information), in plasma, the AUC_0_‐_24_ of FPD‐GELNs/Su was 4.66‐fold higher than that of free Su, with significantly prolonged elimination half‐life (T_1_/_2_, _β_), indicating greater systemic exposure and extended circulation time of the drug. In tumor tissue, the AUC_0_‐_24_ of FPD‐GELNs/Su was 5.36‐fold greater than that of free Su, and the C_max_ was 2.84‐fold higher (Figure 13B; Table S16, Supporting Information). These findings demonstrate that FPD‐GELNs not only improve the systemic pharmacokinetic behavior of Su, but, more importantly, substantially enhance its accumulation in tumor tissue.

### 2.16 In Vivo Biosafety Evaluation

Hemolysis is one of the most common issues induced by nanoformulations. Hemolysis assessment is widely employed in the evaluation of various nanoformulations.^[^ ^42^ ^]^ Therefore, we evaluated the blood compatibility of the nanoformulations through hemolysis assays. All the tested nanoformulations exhibited minimal hemolytic activity, with hemolysis ratios consistently less than 4% (Figure 13C,D). Additionally, throughout the entire treatment period, no significant weight loss was observed in the mice treated with any of the nanoformulations (Figure 13E). At the end of the treatment, H&E staining of major organs (heart, liver, lung, spleen, and kidney) revealed no evident signs of tissue damage (Figure 13F).

To conduct a more comprehensive evaluation of the biological safety of the nanocarriers, we examined potential adverse effects, such as anaemia, infection, coagulation dysfunction, and hepatorenal toxicity via routine blood and biochemical indicators (Figure 13G). Satisfyingly, several nanocarriers did not induce significant changes in red blood cells (RBC), white blood cells (WBC), hemoglobin, or platelet (PLT) in mice. However, GELNs caused a significant increase in alanine aminotransferase (ALT) levels (*p* < 0.001), whereas GELNs/Su led to a significant increase in both ALT and aspartate aminotransferase (AST) levels (*p* < 0.01). Notably, modification with FPD reversed these hepatotoxic effects. With respect to renal toxicity markers such as blood urea nitrogen (BUN) and serum creatinine (CRE), all the tested nanocarriers were found to be safe. Additionally, GELNs and GELNs/Su resulted in an increase in WBC counts (although not statistically significant), accompanied by elevated levels of complement C3 and tumor necrosis factor‐α (TNF‐α) (*p* < 0.05), which may trigger a series of potential cascading reactions, including immune system activation, pathogen clearance, and inflammatory responses. For all the nanocarriers, the levels of the other proinflammatory cytokines IL‐6 and IL‐12, did not differ significantly from those in the control group (Figure 13G). Overall, GELNs and GELNs/Su have the potential to induce hepatotoxicity and pose risks of systemic infection. In contrast, the successful incorporation of FPD markedly enhances the biocompatibility and safety profile of nanomedicines.

### 2.17 Efficacy of Nanoformulations in Inhibiting the Pulmonary Metastasis of RCC

The five most common sites of metastasis in RCC are the lung, lymph nodes, bone, liver, and adrenal glands.^[^ ^43^ ^]^ Therefore, we initially assessed the mRNA expression levels of the top 50 GELN targets (Figure 2C) via data from the BioGPS database. Among these 50 target genes, 48 presented elevated mRNA levels in organs associated with RCC metastasis, including the lung (degree: 38), liver (degree: 37), bone marrow (degree: 23), brain (degree: 15), adrenal gland (degree: 11), and lymph nodes (degree: 11) (Figure S12, Supporting Information). The majority of the GELN target genes were significantly upregulated in multiple tissues or organs involved in RCC metastasis, suggesting the potential clinical utility of GELNs for treating metastatic renal cancer. Additionally, several immune cell types, including CD56^+^ NK cells (degree: 45), CD34^+^ hematopoietic stem cells (degree: 43), CD33^+^ myeloid cells (degree: 41), CD4^+^ T cells (degree: 41), CD14^+^ monocytes (degree: 37), CD105^+^ endothelial cells (degree: 29), and CD8^+^ T cells (degree: 27), strongly correlated with GELN target genes, indicating the potential immunomodulatory effects of GELNs (Figure S12, Supporting Information).

**Figure 14:**
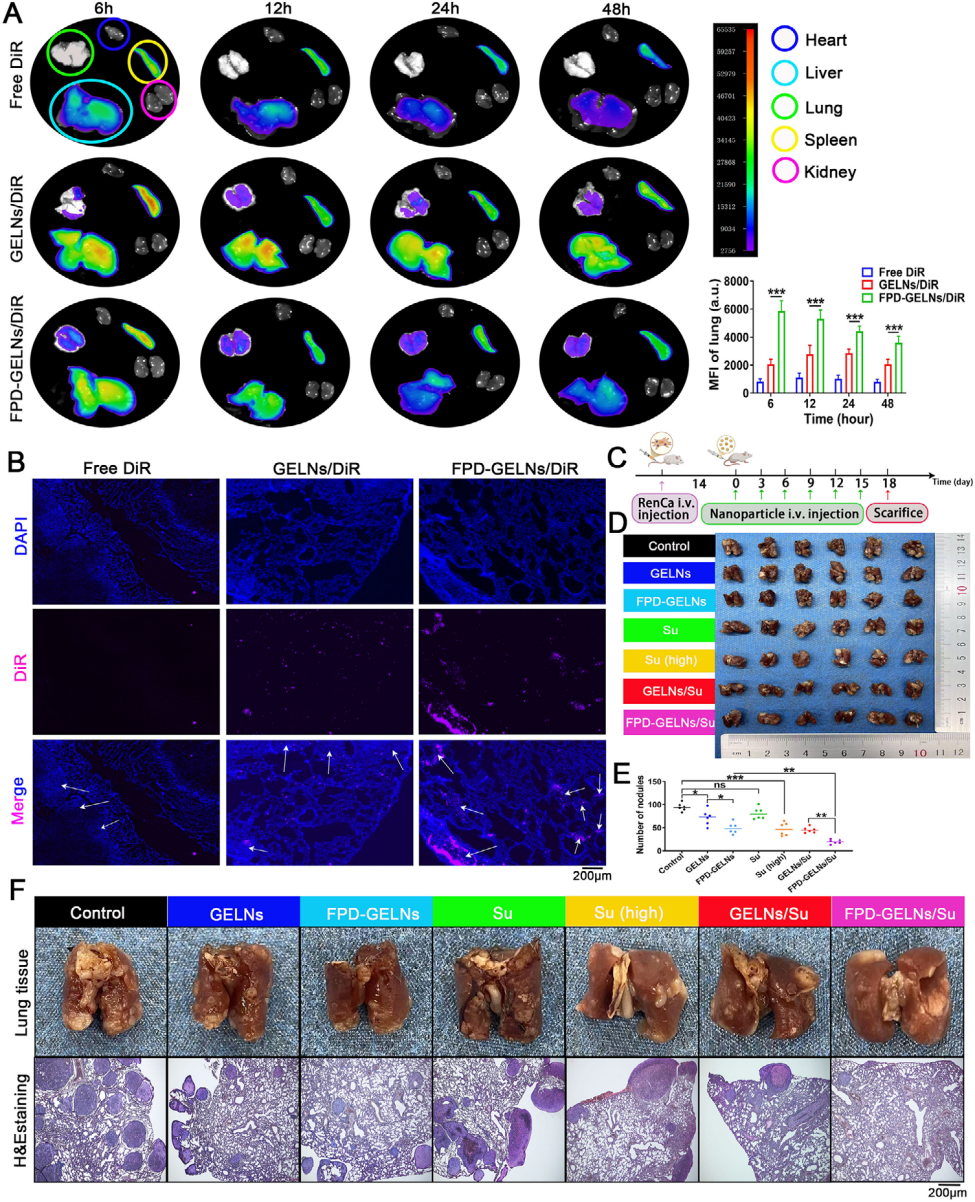
Targeting efficacy of FPD on renal cancer lung metastases and therapeutic effects of nanoformulations. A) Fluorescence images of major organs (heart, liver, lung, spleen, and kidney) in renal cancer lung metastasis mice following a single intervention with free DiR, GELNs/DiR, and FPD‐GELNs/DiR at various time points, along with quantitative analysis of fluorescence intensity in the lungs (n = 3 mice each group). B) Fluorescence images of DiR in lung tissue sections from renal cancer lung metastasis model mice after a single intervention with free DiR, GELNs/DiR, and FPD‐GELNs/DiR (n = 3 independent experiments). C) Schematic representation of the intervention time points for nanoformulations in the renal cancer lung metastasis model mice. D) Images of lung tissues from different intervention groups at the end of treatment, and E) statistical graph of the number of renal cancer lung metastatic nodules (n = 6 mice each group). F) Representative images of lung tissues and H&E stained lung tissue sections from different intervention groups (n = 6 mice each group). Data are presented as mean ± SD. ^*^ *p* < 0.05, ^**^ *p* < 0.01, ^***^ *p* < 0.001. ns, not significant (one‐way ANOVA with Tukey's multiple comparison test). Scale bar = 200 µm.

Given that the lung is the most common site for distant metastasis in RCC, we evaluated the targeting characteristics of FPD‐GELNs for renal cancer lung metastatic nodules. Following a single injection of GELNs/DiR, a sustained accumulation was observed in the lung tissue over 48 h. In contrast, FPD‐GELNs/DiR exhibited significantly greater accumulation in the lung tissue at 6, 12, 24, and 48 h postinjection than GELNs/DiR at the corresponding time points (**Figure** **14**A). After 48 h, fluorescence microscopy revealed that the DiR fluorescence intensity in the lung tissue was greater in the FPD‐GELNs/DiR group than in the GELNs/DiR group. Specifically, while GELNs/DiR showed moderate enrichment in both lung nodules and normal lung tissue, the DiR fluorescence signal in FPD‐GELNs/DiR was predominantly localized to lung nodules, with significantly stronger fluorescence intensity than that in the GELNs/DiR group (Figure 14B). These findings demonstrate the specific targeting capability of FPD‐GELNs for renal cancer lung metastasis.

Based upon the above results, we assessed the therapeutic efficacy of nanoformulations in treating the lung metastasis of renal cancer. The nanoformulations were administered intravenously every 3 days for a total of 6 doses (Figure 14C). While GELNs significantly reduced the number of lung nodules, their antimetastatic efficacy was markedly inferior to that of FPD‐GELNs and GELNs/Su. Consistent with our expectations, FPD‐GELNs/Su achieved the most robust therapeutic outcome (Figure 14D–F). Interestingly, although the Su group exhibited some potential for inhibiting lung metastasis, this effect did not reach statistical significance. To achieve a notable therapeutic effect with Su alone, an increased dosage of Su would be required (Figure 14D–F).

## 3 Discussion

Since the initial discovery of mammalian cell‐derived exosomes (MDEs) by Trams et al. in 1981,^[^ ^44^ ^]^ extensive research has been devoted to understanding their properties and applications. PELNs, which share similar characteristics with MDEs but offer several distinct advantages, have garnered significant attention from the scientific community.^[^ ^45^ ^]^ First, compared with the relatively low yield of MDEs, PELNs can be efficiently produced through large‐scale manufacturing processes from a variety of beneficial medicinal plants.^[^ ^46^ ^]^ Second, the naturally evolved components of PELNs in plant cells have reduced cytotoxicity and immunogenicity, thereby enhancing biocompatibility and safety.^[^ ^15^ ^]^ Additionally, the biological activity and pharmacological functions of PELNs closely resemble those of the original plants and often surpass the efficacy of single active ingredients extracted from plants.^[^ ^47^ ^]^ Owing to the specific disease response elicited by different pharmacological molecules, researchers can selectively extract PELNs from various medicinal plants based on their therapeutic suitability.

The FPD‐GELNs/Su composite formulation we developed exerts inhibitory effects on the progression and metastasis of RCC through synergistic multi‐mechanistic actions. Notably, this therapeutic strategy directly addresses two major challenges in current RCC treatment: drug resistance and the immunosuppressive TME.^[^ ^48^, ^49^ ^]^ Initial investigations using network pharmacology combined with experimental validation revealed that the core anticancer mechanism of GELNs is mediated via inhibition of the PI3K‐Akt signaling pathway. Accumulating evidence suggests that mutations or dysregulation of multiple genes within this pathway contribute to tumor progression and play pivotal roles in regulating cellular metabolism, proliferation, and survival. Aberrant activation or mutation of this pathway can drive sustained cell proliferation, resistance to apoptosis, and metabolic reprogramming, thereby establishing it as a key oncogenic hub in RCC.^[^ ^50^, ^51^ ^]^ Our findings demonstrate that GELNs enriched with 6‐Shogaol, 8‐Shogaol, and 6‐Paradol—bioactive constituents with potential inhibitory activity against the PI3K‐Akt signaling pathway, which inhibits the phosphorylation of PI3K and Akt in a dose‐dependent manner, leading to cell cycle arrest, enhanced apoptosis, and suppression of metastasis in RCC cells. These results provide novel insights into the potential of natural nanocarriers for targeting critical therapeutic pathways in RCC. Furthermore, to achieve a comprehensive understanding of the anti‐RCC mechanisms of GELNs, future studies should investigate the functional contributions of other predicted targets and pathways, as well as their interactions with the PI3K‐Akt signaling axis.

A critical factor contributing to the significant anticancer efficacy of PELNs is their tumor‐targeting ability. Despite this potential, achieving effective tumor targeting remains a major challenge in disease treatment.^[^ ^52^, ^53^ ^]^ Compared with conventional liposomes, PELNs exhibit several‐fold greater tumor tissue‐homing ability because of their small size, negative zeta potential that facilitates prolonged circulation, structural similarity to the plasma membrane, and remarkable physicochemical stability across varying pH levels and temperatures.^[^ ^14^ ^]^ However, in this study, the GELNs exhibited a suboptimal homing ability within the TME of RenCa. In contrast, the FPD‐conjugated GELNs significantly reduced systemic clearance, enhanced the EPR effect, and improved tumor‐targeting efficiency, thereby enhancing therapeutic efficacy. Additionally, compared with the systemic diffusion of targeted drugs, the selective delivery of these drugs via FPD‐conjugated GELN‐based nanocarriers minimizes unnecessary adverse reactions.

ABC transporter proteins play a critical role in facilitating drug efflux, leading to reduced intracellular drug concentrations and diminished therapeutic efficacy.^[^ ^34^ ^]^ P‐gp (ABCB1), multidrug resistance protein 1 (MRP1, ABCC1), and breast cancer resistance protein (BCRP, ABCG2) are the primary ABC transporters responsible for MDR in cancer cells.^[^ ^35^ ^]^ Although several agents, such as verapamil, valspodar, dofequidar, Ko143, and MK571, have been developed as ABC transporter modulators, their clinical success has been limited due to their low efficacy, adverse side effects, non‐specificity, and uncertain drug interactions.^[^ ^54^ ^]^ Previous studies have shown that various TKIs, including Su, serve as substrates for P‐gp‐mediated transport.^[^ ^36^, ^37^ ^]^ In this study, multiple pharmacologically active components within GELNs synergistically suppressed the mRNA and protein expression of ABCB1, thereby reducing Su efflux, enhancing its intracellular accumulation, and increasing its therapeutic sensitivity. This multi‐component synergistic inhibition strategy may offer significant advantages over conventional single‐agent P‐gp inhibitors, which are often associated with toxicity and limited effectiveness. The incorporation of FPD into GELNs further amplified the Su sensitization effect induced by the downregulation of ABCB1/P‐gp expression mediated by GELNs. Specifically, FOLR‐targeted delivery not only enhanced the intracellular uptake of GELNs/Su in tumor cells but also more effectively attenuated the P‐gp‐mediated drug resistance barrier, thereby markedly improving the cytotoxic activity of Su and its inhibitory effect on lung metastasis. These findings suggest a novel combinatorial strategy for overcoming drug resistance in the targeted therapy of RCC.

In addition to their intrinsic anti‐tumor effects and the potential to enhance TKI sensitivity, GELNs also modulate the TME and host immune responses. M2 polarization of TAMs represents a key hallmark of the immunosuppressive TME and contributes significantly to therapeutic resistance in RCC.^[^ ^55^ ^]^ Therefore, reprogramming TAM polarization from the M0 to M1 phenotype or from M2 to M1 phenotype may represent a promising strategy for cancer therapy.^[^ ^44^ ^]^ Previous studies have demonstrated that ginseng‐derived ELNs can alleviate the suppressive effects of M2 macrophages on naïve CD8^+^ T cell proliferation and promote M1 polarization via the TLR4‐MyD88‐dependent signaling pathway. These ELNs have been shown to act as immunomodulators in melanoma tissues, suggesting their potential application in cancer immunotherapy.^[^ ^56^ ^]^ Our study confirmed that the immune response induced by GELNs primarily involves the polarization of TAMs through evasion of negative regulatory signals, followed by the mobilization of T cell‐mediated immunity. This includes an increased M1/M2 ratio, altered expression levels of M1‐ and M2‐specific markers, enhanced CD8^+^ T cell infiltration, and potential modulation of MDSC function. Notably, GELN‐derived metabolites targeting the PI3K‐Akt signaling pathway, together with the mes‐miR477 and gma‐miR164 families implicated in the regulation of efferocytosis signaling, may play a critical role in mediating these effects. FPD modification significantly amplified this effect by specifically targeting TAMs with high FOLR2 expression. The dual activation of both innate immunity (TAMs) and adaptive immunity (T cells), combined with direct tumor cell cytotoxicity mediated by PI3K‐Akt inhibition and reversal of drug resistance through P‐gp downregulation, constitutes the core mechanism underlying the potent anti‐tumor and anti‐metastatic efficacy of FPD‐GELNs/Su. Histologically, these effects are characterized by reduced cellular proliferation (as indicated by Ki67 staining), decreased angiogenesis (assessed by CD34 expression), and enhanced apoptosis (demonstrated by TUNEL assay). Collectively, these findings indicate that GELN‐based nanomedicines effectively activate the anti‐tumor potential of TAMs and promote subsequent T cell immune responses.

Compared with other reported PELNs, such as Artemisia annua‐derived ELNs that activate the cGAS‐STING pathway^[^ ^27^ ^]^ and ginseng‐derived ELNs that promote M1 macrophage polarization.^[^ ^28^ ^]^ This study offers several distinct advantages. First, it represents the first integration of network pharmacology analysis into GELN research, elucidating the anti‐tumor mechanisms from a “multi‐component, multi‐target, multi‐pathway” perspective. Second, in contrast to previous studies on tea flower‐derived, tea leaf‐derived or ginseng‐derived ELNs,^[^ ^28^, ^57^, ^58^ ^]^ this work moves beyond the conventional use of single‐source PELNs by strategically combining GELNs with both active (FA) and passive (PEG) targeting strategies, along with the encapsulation of the TKI (Su). This innovative approach establishes a novel paradigm of “network pharmacology‐guided targeted nanotherapy”, providing a structured framework for the systematic investigation and efficient exploitation of the complex pharmacological profiles of PELNs. Notably, GELNs not only exert intrinsic anti‐cancer activity but also synergize with the encapsulated therapeutic agent, thereby enhancing drug sensitivity. Furthermore, the pharmacological efficacy of PELNs relies on the successful transport of their bioactive constituents‐compounds with high bioavailability and permeability‐across the intestinal epithelium into systemic circulation, a process subject to significant physiological barriers.^[^ ^47^ ^]^ The oral delivery route cannot guarantee structural integrity or efficient systemic utilization of PELNs. Notably, the majority of pharmacologically active compounds in GELNs fail to meet established oral drug‐likeness criteria‐specifically, oral bioavailability ≥ 30% and drug‐likeness ≥ 0.18‐suggesting that these components are likely to undergo extensive degradation in the gastrointestinal tract and exhibit limited systemic bioavailability. Therefore, the intravenous administration strategy employed in this study was designed to maximize the delivery of potential bioactive constituents, ensuring broader exposure and enhanced therapeutic potential. Certainly, to emphasize the significance of the oral route in this biological process, further engineering of the nanoformulations can be pursued. For example, GELNs could be encapsulated within pH‐responsive carriers designed to enable site‐specific release in the gastrointestinal tract in response to local pH variations.^[^ ^59^ ^]^ Alternatively, the active compounds currently embedded within the phospholipid bilayer of GELNs could be isolated and re‐encapsulated using liposomal systems with enhanced intestinal permeability‐such as the outer membranes of extracellular vesicles (EVs) derived from mammalian milk.^[^ ^60^ ^]^ These biological membranes contain lipids, proteins, and other bioactive components that may confer protective effects against harsh gastrointestinal conditions, thereby improving the stability of the encapsulated agents and increasing their likelihood of traversing the intestinal barrier for systemic absorption. These strategies present a promising avenue for future development of oral GELN‐based delivery systems.

Although the screening criterion “carcinogenicity < 0.3” was introduced to comprehensively evaluate the biological effects of individual pharmacological molecules, the remaining components of GELNs, such as benzene rings, aldehydes, and alkaloids, and others, pose potential biological hazards, including skin irritation, eye damage, respiratory toxicity, hepatorenal toxicity, and even carcinogenicity.^[^ ^61^, ^62^, ^63^, ^64^ ^]^ Therefore, it is crucial to conduct a comprehensive evaluation of the pharmacological and toxicological profiles of GELN components to facilitate their clinical application. On the other hand, precise dosage control of GELNs is critically important. Previous studies have demonstrated that administration of 3 mg kg^−1^ tea leaf‐derived ELNs led to elevated AST levels within the therapeutic window in a murine breast cancer model.^[^ ^57^ ^]^ Given the ≈10% DL capacity of GELNs in this study, treatment with 20 mg kg^−1^ GELNs or GELNs/Su (containing 2 mg kg^−1^ Su) also resulted in increased ALT levels, indicating potential hepatotoxicity. Notably, FPD modification significantly improved the in vivo biodistribution of GELNs, mitigated liver function impairment, and enhanced tumor accumulation of Su by 5.36‐fold. Furthermore, the Su dose used in this study (2 mg kg^−1^) is lower than doses reported in many previous studies, which can be attributed to several key considerations: (i) the synergistic therapeutic strategy employed, wherein GELNs themselves exert intrinsic antitumor effects and enhance Su sensitivity; (ii) the improved bioavailability and tumor‐targeting efficiency conferred by the nanocarrier system, enabling effective drug concentrations at the tumor site even at low systemic doses; (iii) the avoidance of higher Su doses, which would necessitate increased GELN administration and thereby impose a greater metabolic burden on the animals; (iv) the intentional use of a lower dose to minimize systemic toxicity of Su, thereby accentuating the safety and efficacy advantages of the nanoparticle‐based delivery platform; and (v) The primary route of administration employed in this study was intravenous injection, which provides higher drug bioavailability compared to oral or intraperitoneal delivery.

Future efforts should focus on comprehensive pharmacological and toxicological profiling of GELN components, along with optimization of key nanocarrier characteristics—such as low drug loading capacity—to facilitate clinical translation. Key research priorities include: (i) employing advanced separation techniques (e.g., size‐exclusion chromatography) or bioengineering strategies to enrich therapeutic constituents while eliminating potentially harmful components;^[^ ^53^, ^65^ ^]^ (ii) conducting rigorous, Good Laboratory Practice (GLP)‐compliant toxicological assessments over extended durations and across multiple species (e.g., rats, dogs) to evaluate chronic and reproductive toxicity; and (iii) establishing stringent quality control standards for GELN composition to ensure batch‐to‐batch consistency and safety.

## 4 Conclusion

In summary, the anti‐cancer efficacy of FPD‐GELNs/Su is mediated through a multifaceted mechanism: GELNs inhibit renal cancer progression by suppressing the phosphorylation of the PI3K‐Akt signaling pathway; FPD potentiates Su sensitivity by reducing ABCB1/P‐gp expression targeted by GELNs; and FPD amplifies the immune effect of GELNs in remodeling the TME by changing the M1/M2 ratio and mobilizing T cells for the immune response. The FPD‐GELNs/Su leverages multiple tumor‐responsive mechanisms to inhibit tumor progression, which holds significant potential for clinical translation and may revolutionize the treatment paradigm for RCC.

## 5 Experimental Section

### Isolation and Purification of GELNs

Fresh ginger was procured from the local farmers' market, peeled, and washed thoroughly. It was then mixed with phosphate‐buffered saline (PBS) at an appropriate ratio and homogenized in a high‐speed blender for 5 min. In accordance with a previously published protocol,^[^ ^66^ ^]^ differential centrifugation combined with sucrose gradient ultracentrifugation was employed to collect the 8/30% and 30/45% density bands as GELNs. Specifically, the 8/30% layer was designated as GELNs1, whereas the 30/45% layer was designated as GELNs2. GELNs1 and GELNs2 were subsequently combined to generate the total GELNs population. The protein concentrations of GELNs1, GELNs2, and the total GELNs were quantified via a BCA protein assay kit (Beyotime, Shanghai, China).

### Identification and Characterization of GELNs

The morphology of GELNs1 and GELNs2 was examined via TEM (Hitachi Limited, H‐7500, Tokyo, Japan). NTA was conducted with a Zetaview (ParticleMetrix, PMX120‐Z, Munich, Germany) to assess their particle size and distribution. The zeta potential was measured via a Zetasizer (Malvern Instruments, Nano ZS90, Malvern, UK).

### Metabolomics, Proteomics, and miRNA Sequencing of GELNs

CELNs1, GELNs2, and GELNs were stored at −80 °C. Metabolomics analyses were conducted on GELNs1 and GELNs2, while proteomics and miRNA sequencing analyses were performed on GELNs by Majorbio Bio‐Pharm Technology Co., Ltd (Shanghai, China). Subsequent disease‐associated analyses of the identified metabolites, proteins, and miRNAs were carried out using the Majorbio online analysis platform (cloud.majorbio.com).

### Prediction of Targets for GELN Active Ingredients and RCC‐Related Targets

Owing to the intravenous administration used in the animal experiments of this study, traditional screening criteria such as oral bioavailability ≥ 30% and drug‐likeness ≥ 0.18 were not applicable for identifying the bioactive components of GELNs. Therefore, all the plant‐derived metabolites of the GELNs were imported into the ADMETlab 3.0 database (http://admetlab3.scbdd.com/server/screening), with the screening criterion set at “carcinogenicity < 0.3”. The potential targets of these bioactive components were predicted via the TCMSP (http://old.tcmsp‐e.com/tcmsp.php) and SwissTargetPrediction (http://www.swisstargetprediction.ch/) databases, with species‐specific predictions limited to Homo sapiens. Additionally, the SwissTargetPrediction database was also utilized to predict the targets of the bioactive components for Mus musculus.

Additionally, RCC‐related targets were retrieved from the following databases: DrugBank 6.0 (https://go.drugbank.com/), GeneCards (https://www.genecards.org/; the top 2000 targets were selected on the basis of the relevance score), OMIM (https://www.OMIM.org/), and TTD (https://db.idrblab.net/ttd/). The search keyword used was “Renal cell carcinoma”. All gene names were standardized via the UniProt database (https://www.uniprot.org/uploadlists/). Furthermore, mouse RCC‐related targets were obtained from the BioGRID ORCS database (https://orcs.thebiogrid.org/). A Venn diagram was used to visualize the intersection between the targets of GELN active ingredients and RCC‐related targets, defining the overlap as the potential molecular targets through which GELNs may regulate RCC progression.

### Construction of the Drug Regulatory Network for the Bioactive Components of GELNs

The intersecting targets between each bioactive pharmacological component of GELNs and RCC were identified and compiled into a network file representing the regulatory network of GELNs on RCC. The drug‐target interaction networks for both human and murine RCC were subsequently visualized via Cytoscape software 3.10.2 (https://www.cytoscape.org/).

### Identification and Screening of Core Hub Genes (CHGs) and Comprehensive Analysis of Pathway Enrichment

First, GO and KEGG enrichment analyses of the intersecting targets were performed via R software 4.4.1. The intersecting targets were subsequently imported into the STRING database (https://cn.string‐db.org/) to construct a PPI network. The species search was limited to Homo sapiens or Mus musculus, with a medium confidence score threshold of > 0.9, and isolated nodes were removed. Using Cytoscape software, the top 10 genes with the highest degree centrality were identified as CHGs. To gain a comprehensive understanding of how CHGs regulate RCC progression, they were analyzed via the ClueGO plugin in Cytoscape, with the following settings: network specificity set to medium, *p*‐value filtering condition set to < 0.05, cluster set to 3, and kappa score limited to 0.1. The regulatory network between CHGs and specific pathways was visualized via the CluePedia and yFiles layout algorithm plugins. Finally, a drug regulatory subnetwork targeting the identified CHGs was constructed.

### Screening of Characteristic Genes Associated with M2‐to‐M1 Polarization

The GEO database (https://www.ncbi.nlm.nih.gov/geo/) was utilized to retrieve five microarray datasets (GSE123180, GSE159762, GSE181223, GSE242723, and GSE247106) profiling mRNA expression in mouse M1 and M2 macrophages. Differentially expressed genes (DEGs) were identified using the “limma” package in R software with the threshold of *p* < 0.05 and |logFC| > 1. The “RobustRankAggreg” (RRA) package was applied for integrative analysis across multiple microarray datasets. To address potential batch effects, the “SVA” package was employed to normalize and integrate the five datasets, followed by DEG detection under the same criteria (*p* < 0.05, |logFC| > 1). Finally, DEGs identified by both RRA and batch‐corrected integration were combined to determine the characteristic gene signature associated with M2‐to‐M1 macrophage polarization.

### Regulation of Macrophage Polarization by miRNAs and Metabolites Derived from GELNs

The psRobot database (https://www.omicslab.genetics.ac.cn/psRobot/index.php) was employed to predict mouse target genes of miRNAs identified in GELNs. Subsequently, an intersection analysis was performed among these predicted target genes, the target genes associated with GELNs‐derived metabolites, and the characteristic genes involved in M2‐to‐M1 macrophage polarization. This approach aimed to identify key regulatory genes mediating macrophage polarization through the combined effects of GELNs’ miRNAs and metabolites. Functional enrichment of these regulatory genes was analyzed using the KEGG pathway database to identify significantly enriched signaling pathways.

### Molecular Docking

To investigate the potential binding efficacy of active components in the drug regulatory subnetwork with CHGs, molecular docking technology was employed. Specifically, the 2D structures of all the active components were retrieved from the PubChem database (https://pubchem.ncbi.nlm.nih.gov/) and subsequently optimized into 3D structures via Chem3D software (https://www.3dchem.com/). The crystal structures of the CHGs were obtained from the RCSB Protein Data Bank (PDB, https://www.rcsb.org/), and water molecules as well as other ligands were removed via PyMOL software version 3.0 (https://www.pymol.org/). Molecular docking simulations were conducted using AutoDock Tools 1.5.6 (http://autodock.scripps.edu/) and AutoDock Vina 1.1.2 (https://vina.scripps.edu/), which encompass multiple docking modes and binding energy calculations, from which the optimal results were selected. Additionally, key ligand‐receptor interactions were analyzed via the Protein‐ligand Interaction Profiler (PLIP) database (http://plip‐tool.biotec.tu‐dresden.de/plip‐web/plip/index/) and visualized via PyMOL.

### MD Simulation

MD simulations were utilized to evaluate the stability and dynamic interactions between the protein and ligand following molecular docking. Using the GROMACS 2024 software suite, a 100 ns all‐atom MD simulation was performed on the docked complex. Initially, the receptor and ligand data were extracted, and missing atoms were added using SPDBV 4.10 software. The ligand was parameterized with the GAFF force field, whereas the protein receptor was modeled using the CHARMM36 force field in conjunction with the TIP3P water model. Subsequently, the protein and small‐molecule ligand files were combined to construct the simulation system, which was solvated with water molecules. Sodium and chloride ions were then introduced to neutralize the system's charge. Following energy minimization, the V‐rescale temperature coupling method was employed to maintain the simulation at 300 K, while the Berendsen barostat was used to control the pressure at 1 bar. Under these conditions, NVT and NPT equilibration phases were conducted for 2 ns each, followed by a 100 ns production run, with trajectory frames saved every 2 fs. Finally, the gmx rms, gmx rmsf, gmx gyrate, gmx sasa, and gmx hbond modules in GROMACS were used to compute the RMSD, RMSF, Rg, SASA, and hydrogen bond interactions of the protein. The resulting dynamic data were visualized using qtgrace software version 2.6. Additionally, the free energy landscape was analyzed based on the RMSD and Rg trajectory profiles.

### Determination of Key Active Constituents in GELNs

A volume of 200 µL of GELNs and ginger juice was processed through extraction, low‐temperature ultrasonication, nitrogen evaporation, phase extraction, centrifugation, and reconstitution to prepare samples suitable for instrumental injection. The concentrations of 6‐Shogaol, 8‐Shogaol, 6‐Paradol, and Ferulic acid were determined using an Agilent 1290 Infinity II Reversed‐phase high‐performance liquid chromatograph(UHPLC)system (CA, USA) equipped with a Kinetex C18 column (100 mm × 2.1 mm, 2.6 µm, CA, USA). Calibration curves were constructed using standard solutions of the target analytes—6‐Shogaol, 8‐Shogaol, 6‐Paradol, and Ferulic acid—at concentrations ranging from 1 to 1000 ng mL^−1^ (MedChemExpress, NJ, USA). The mobile phase consisted of solvent A (2% acetonitrile in water containing 0.1% formic acid) and solvent B (acetonitrile containing 0.1% formic acid). The flow rate was set at 0.4 mL min^−1^, the column temperature was maintained at 40 °C, and the injection volume was 3 µL.

6‐Shogaol, 8‐Shogaol, and 6‐Paradol were analyzed in positive ionization mode with a total run time of 16 min; the gradient program is detailed in Table S17 (Supporting Information). Ferulic acid was analyzed in negative ionization mode with a total run time of 13 min; the corresponding gradient profile is provided in Table S18 (Supporting Information).

### Cell Culture

The mouse‐derived renal adenocarcinoma RenCa cell line, the human‐derived clear cell renal cell carcinoma 786‐O and OS‐RC‐2 cell lines, and the human renal cortical proximal tubular epithelial HK‐2 cell line were cultured in a complete medium consisting of 89% RPMI‐1640, 10% fetal bovine serum (FBS), and 1% penicillin/streptomycin (P/S). The cells were maintained at 37 °C in a humidified atmosphere containing 5% CO_2_. The cells were passaged using 0.25% trypsin when they reached 80–90% confluence.

### 3‐(4, 5‐Dimethyl‐2‐thizolyl)‐2, 5 Diphenyltetrazolium Bromide (MTT) Toxicity Assay

The RenCa, 786‐O, OS‐RC‐2, and HK‐2 cell lines were seeded at a density of 3000 cells per well in a 96‐well plate and incubated overnight at 37 °C in a humidified atmosphere containing 5% CO_2_. The medium was then replaced with fresh medium containing GELNs (Concentrations ranging from 1 to 64 µg mL^−1^), and the cells were incubated for 24, 48, and 72 h. Subsequently, MTT reagent (10 µL; Beyotime, Shanghai, China) was added to each well, and the plate was incubated at 37 °C for 4 h. Afterward, formazan dissolution solution (100 µL; Beyotime) was added to dissolve the dark purple formazan crystals. Once the crystals were completely dissolved, the absorbance was measured at 570 nm via a microplate reader (Thermo Fisher, MA, USA). Cell viability was calculated via the following formula:^[^ ^18^ ^]^

(1) $$\mathrm{Cell}\;\mathrm{viability}\left(\%\right)=\left(OD_{\mathrm{treated}}-OD_{\mathrm{blank}}\right)/\left(OD_{\mathrm{control}}-OD_{\mathrm{blank}}\right)$$

### Colony Formation Assay

RenCa and 786‐O cells were seeded in 6‐well plates at a density of 1000 cells per well and incubated overnight. The cells were subsequently treated with varying concentrations of GELNs every two days for a total incubation period of 2 weeks. After the incubation, the colonies were fixed with 4% paraformaldehyde (Biosharp, Beijing, China) for 30 min at room temperature, stained with 0.1% crystal violet solution (Beyotime) for 30 min, and then washed with PBS. Colonies were photographed, and the number of colonies was quantified via Image J software (Wayne Rasband, MD, USA).

### 5‐Ethynyl‐2′‐deoxyuridine (EdU) Assay

The EdU assay was conducted via the BeyoClick EdU‐488 cell proliferation assay kit (Beyotime) to evaluate the impact of GELNs on cell proliferation. The experimental protocol was as follows: RenCa and 786‐O cells were treated with GELNs (8 µg mL^−1^) for 24 h, followed by incubation with EdU solution for 2 h. Subsequently, the cells were fixed with 4% paraformaldehyde for 30 min at room temperature and stained with DAPI (BOSTER, Wuhan, China) to label the nuclei. Finally, images were captured via a fluorescence microscope (Leica, DMI8, Wetzlar, Germany), and the number of EdU‐positive cells was quantified via Image J software.

### Cell Apoptosis Assay

To evaluate the ability of GELNs to induce apoptosis in RCC cells, RenCa and 786‐O cells were seeded at a density of 5 × 10^5^ cells per well in 6‐well plates and incubated overnight. The next day, the cells were treated with GELNs (8 µg mL^−1^) for 24 h. Following treatment, the cells were trypsinized with 0.25% trypsin‐EDTA to prepare single‐cell suspensions. The resulting cell suspensions were washed twice with cold PBS and then stained with Annexin V‐FITC and propidium iodide (PI) according to the manufacturer's protocol. Apoptosis was analyzed by FCM via a Cytoflex flow cytometer (Beckman Coulter, CA, USA).

### Cell Cycle Detection

In accordance with the previously described methods for determining apoptosis, single‐cell suspensions of RenCa and 786‐O cells were prepared following GELN treatment. The cells were subsequently fixed overnight at 4 °C using prechilled 70% ethanol. PI staining was then applied to the cells, followed by cell cycle analysis via FCM.

### Wound Healing Assay

On the reverse side of each well in a 12‐well plate, three horizontal lines were drawn as reference marks. RenCa and 786‐O cells were then seeded into the wells and cultured until they reached 90% confluence. Subsequently, three parallel scratches were made perpendicular to the reference lines via the tip of a 200 µL pipette, perpendicular to the reference lines. The cells were treated with a GELN solution (8 µg mL^−1^) for 24 h. Images of the scratch areas were captured at 0 h (baseline) and 24 h (posttreatment) via an inverted microscope (Nikon, Tokyo, Japan). Relative migration rates were analyzed via Image J software.

### Transwell Assay

For the Transwell invasion assay, RenCa and 786‐O cells (5 × 10^4^ cells per well in 200 µL of serum‐free RPMI‐1640 medium containing 8 µg mL^−1^ GELNs) were seeded into the upper chamber (pore size: 8 µm; Biofil, Guangzhou, China) precoated with Matrigel (Beyotime). To promote cell invasion, RPMI‐1640 medium (600 µL) containing 10% FBS was added to the lower chamber as a chemoattractant. After a 24‐h incubation period, noninvading cells on the upper surface of the membrane were gently removed via a cotton swab. The invading cells on the lower surface were then fixed with 4% paraformaldehyde for 30 min and stained with 0.1% crystal violet solution for 30 min. After three washes with PBS, images of the invading cells were captured via an upright microscope (Olympus, Tokyo, Japan), and the number of invading cells in each field of view was quantified via Image J software.

### Western Blot Analysis

Phenylmethylsulfonyl fluoride (PMSF, Servicebio, Wuhan, China) and RIPA lysis buffer (Servicebio) were mixed at a 1:100 ratio to lyse the cell samples. Protein concentrations were quantified via a BCA assay. The lysates were then subjected to sodium dodecyl sulfate‐polyacrylamide gel electrophoresis (SDS‐PAGE) and transferred onto polyvinylidene fluoride (PVDF, Servicebio) membranes. The membranes were blocked at room temperature for 1 h in 5% skim milk diluted in TBST. Primary antibodies against PI3K (1:1000; Servicebio, GB112375), p‐PI3K (1:1000; Affinity, AF3242, OH, USA), AKT (1:1000; Servicebio, GB15689), and p‐AKT (1:1000; Servicebio, GB150002), P‐glycoprotein (P‐gp; 1:1000; Servicebio, GB115178), β‐actin (1:5000; Servicebio, GB15003), and GAPDH (1:10000; Servicebio, GB15004) were incubated with the membranes overnight at 4 °C according to the manufacturer's instructions. The membranes were then incubated with an HRP‐conjugated secondary antibody (1:5000; Servicebio, GB23303) at room temperature for 30 min. The protein blots were subsequently imaged via a chemiluminescence instrument (Servicebio, SCG‐W3000). The intensity of the relevant bands was quantified via AIWBwell software (Servicebio).

### Preparation of Nanoformulations

To load Su (MedChemExpress) into the GELNs, a DMSO solution of Su was mixed with the GELN solution at various ratios (m/m). The mixture was stirred at a speed of 200 rpm min^−1^ for 12 h at room temperature in the dark to facilitate the evaporation of organic solvents.^[^ ^67^ ^]^ Subsequently, unencapsulated Su was removed by ultracentrifugation at 150 000 × g for 60 min at 4 °C. The resulting Su‐loaded GELNs (GELNs/Su) were resuspended in PBS. The EE and DL of Su were determined via a UV spectrophotometer (Shimadzu, UV2600, Kyoto, Japan), and the optimal GELN/Su formulation was determined. EE and DL were calculated according to the following formulas:^[^ ^21^ ^]^

(2) $$\mathrm{EE}\;\left(\%\right)=\left(\mathrm{Weight}\;\mathrm{of}\;\mathrm{loading}\;\mathrm{drug}/\mathrm{Weight}\;\mathrm{of}\;\mathrm{input}\;\mathrm{drug}\right)\times 100\%$$

(3) $$\begin{array}{cc} & \mathrm{DL}\;\left(\%\right)\\ & \;=\left(\mathrm{Wieght}\;\mathrm{of}\;\mathrm{loading}\;\mathrm{drug}/\left(\mathrm{Weight}\;\mathrm{of}\;\mathrm{loading}\;\mathrm{drug}+\mathrm{Weight}\;\mathrm{of}\;\mathrm{GELNs}\right)\right)\\ & \;\times 100\% \end{array}$$

FPD was purchased from Ruixi Biotechnology Co., Ltd. (Xi'an, China). The structure of FPD was characterized via ^1^H‐NMR (Bruker, AVANCE III HD 600 MHz, Karlsruhe, Germany) and FTIR spectroscopy (Thermo Fisher, Nicolet iS50, MA, USA). The GELNs or the optimized GELNs/Su were subsequently mixed with FPD at a 1:1 mass ratio (m/m) and stirred continuously at a speed of 200 rpm min^−1^ for 2 h in the dark at 37 °C via a magnetic stirrer. The resulting mixture was transferred to a 10 kDa ultrafiltration tube (Millipore, MA, USA) and centrifuged at a speed of 5000 rpm min^−1^ for 10 min to remove unbound FPD. The final solution was collected to obtain pure FPD‐GELNs or FPD‐GELNs/Su.

### Determination of FPD Conjugation Efficiency

The optimal absorption wavelength of the FPD standard solution was determined using a UV spectrophotometer, and a standard concentration curve for FPD was established by coupling UV detection with HPLC (Shimadzu, LC‐2030). The 10 µL sample of the free FPD liquid after ultrafiltration was injected and separated on an Agilent C18 chromatographic column (250 mm × 4.6 mm, 5 µm). The mobile phase consisted of acetonitrile (phase A) and ammonium acetate (10 mм) containing 1% acetic acid (phase B). Isocratic elution was performed at a ratio of 55:45 (A:B) with a flow rate of 1 mL min^−1^. The column temperature was maintained at 35 °C. Conjugation efficiency was calculated according to the following formulas:^[^ ^68^ ^]^

(4) $$\begin{array}{cc} & \mathrm{Conjugation}\;\mathrm{efficiency}\;\left(\%\right)\\ & \;=(\left(\mathrm{Total}\;\mathrm{FPD}-\mathrm{free}\;\mathrm{FPD})/\mathrm{Total}\;\mathrm{FPD}\right)\times 100\% \end{array}$$

### Characterization of Nanoformulations

The morphology of all the nanoformulations was characterized via TEM. Dynamic light scattering (DLS; ParticleMetrix, PMX120‐Z) was employed to determine the particle size, PDI, and zeta potential of all the nanoformulations, including the characteristic changes in GELNs/Su and FPD‐GELNs/Su over a period of 3 months. The incorporation of FPD was confirmed by FTIR spectra within the wavenumber range of 400–4000 cm^−1^.

### The Release Kinetics of Su

To investigate the in vitro release kinetics of Su from GELNs/Su and FPD‐GELNs/Su, 50 mL of PBS with three different pH values (pH 5.6, pH 6.6, and pH 7.4) was used as the release medium. Specifically, GELNs/Su or FPD‐GELNs/Su (total Su: 500 µg) in PBS solution (2 mL) was sealed in a dialysis bag with a molecular weight cut‐off of 8000–14000 Da (Sorlabio, Beijing, China). The dialysis bag was then placed in a beaker containing the corresponding release medium (50 mL), which was sealed at the top. The beaker was continuously shaken at a speed of 200 rpm min^−1^ in a dark environment at 37 °C for 48 h. At predetermined time intervals, 1 mL of dialysate was collected for UV spectrophotometric analysis to measure the concentration of Su, and an equal volume of fresh PBS was added to maintain the total volume.

### Bioinformatics Analysis and RT‐qPCR Validation of FOLR1 and FOLR2

Renal cell carcinoma cohorts, including ccRCC (TCGA‐KIRC), pRCC (TCGA‐KIRP), and chRCC (TCGA‐KICH) cohorts, were downloaded from the The Cancer Genome Atlas (TCGA) database (https://portal.gdc.cancer.gov/). Using the CIBERSORT algorithm implemented in R software, the differences and correlations were evaluated in the infiltration levels of 22 immune cell types between the high and low expression groups of FOLR1 and FOLR2.

Following established protocols, bone marrow‐derived macrophages (BMDMs) were isolated from 8 week‐old female BALB/c mice bearing RenCa tumors.^[^ ^27^ ^]^ The cells were cultured in RPMI‐1640 medium supplemented with 10% FBS and 20 ng mL^−1^ recombinant mouse macrophage colony‐stimulating factor (M‐CSF; PeproTech, 315‐02‐2, NJ, USA). The culture medium was replaced every 3 days with fresh medium containing M‐CSF (20 ng mL^−1^). On day 7 of culture, the cells were differentiated into M1‐type macrophages by treatment with 100 ng mL^−1^ lipopolysaccharide (LPS; Sigma–Aldrich, L2880, MO, USA) for 48 h, or into M2‐type macrophages by treatment with 20 ng mL^−1^ recombinant mouse interleukin‐4 (IL‐4; PeproTech, 214‐14‐5) for 48 h.

Total RNA was extracted from various cell lines and RenCa tumor tissues via TRIzol reagent (Takara, Shiga, Japan). cDNA synthesis was performed via the ABScript Neo Master Mix for qPCR with gDNA Remover (ABclonal, Wuhan, China) according to the manufacturer's protocol. RT‐qPCR analysis of FOLR1 and FOLR2 was conducted via the 2X Universal SYBR Green Fast qPCR Mix following the manufacturer's instructions. The relative mRNA expression levels were quantified via a LightCycler480 real‐time PCR system (Roche, Basel, Switzerland), with GAPDH serving as the internal normalization control. All primers were synthesized by Tsingke Biotechnology Co., Ltd. (Beijing, China), and their sequences are provided (Table S19, Supporting Information).

### In Vivo Biodistribution Analysis of GELNs and FPD‐GELNs

To track the in vivo biodistribution of the GELNs and FPD‐GELNs, DiR (Maokangbio, Shanghai, China) was used for labeling. A total of 200 µL of RenCa cells, suspended at a concentration of 5 × 10^6^ cells mL^−1^, were subcutaneously injected (s.c.) into the right axilla of 6‐week‐old female BALB/c mice. Once the tumors reached a predetermined size, each mouse received a single intravenous injection (i.v.) of GELNs/DiR or FPD‐GELNs/DiR (20 mg kg^−1^ dose^−1^). The tumor‐targeting ability of FPD was evaluated at specified time points (6, 12, 24, and 48 h) via an in vivo imaging system (IVIS; Berthold, LB983, Stuttgart, Germany). Additionally, frozen sections (10 µm) were prepared, and the DiR fluorescence signal was subsequently observed via a multichannel fluorescence scanner (Kfbio, KF‐FL‐020, Ningbo, China).

### In Vitro Evaluation of FOLR1+ Tumor Cell Targeting and Cytotoxicity of Nanoformulations

To evaluate the tumor cell‐targeting ability of the GELNs and FPD‐GELNs, DiO (Maokangbio, Shanghai, China) was used for labeling. GELNs/DiO and FPD‐GELNs/DiO (8 µg mL^−1^) were prepared and coincubated with RenCa, 786‐O, or HK‐2 cells. The cells were harvested at predetermined time points (1, 2, 4, 8, 12, and 24 h), and their uptake ratios were assessed via FCM. Additionally, at the 24 h, the DiO fluorescence signal was visualized via fluorescence microscopy.

The in vitro cytotoxicity of various nanoformulations on RCC cells was evaluated via the MTT assay. Specifically, RenCa and 786‐O cells were seeded at a density of 3000 cells per well in 96‐well plates and cultured overnight. The cells were then coincubated with nanoformulations containing different concentrations of Su (0.25–16 µм) for 24 h. Following this incubation period, cell viability was assessed via the MTT assay as previously described. Additionally, combination drug efficacy curves were generated via Compusyn software (https://www.combosyn.com).^[^ ^69^ ^]^

To further evaluate the cellular uptake of Su, various nanoformulations were coincubated with RenCa and 786‐O cells at a concentration of 1 µм Su for 24 h. The intracellular accumulation of Su was observed and imaged. The average fluorescence intensity (MFI) of Su in each treatment group was quantified via Image J software. Additionally, total RNA and total protein were extracted from the tumor cells of all the groups. RT‐qPCR and Western blot analyses were performed to assess the mRNA expression of the ABCB1 gene and the protein level of P‐gp, respectively, following previously described protocols.

### Ex Vivo Targeting and Polarization of FOLR2+ Macrophages by Nanoformulations

To evaluate the targeting efficiency of FPD, both BMDMs in the M0 state and M2 macrophages induced by polarization were utilized. M0 and M2 macrophages were coincubated with GELNs/DiO and FPD‐GELNs/DiO. The cells were harvested at predetermined time points (1, 2, 4, 8, 12, and 24 h), and their uptake ratios were assessed via FCM. Additionally, at 24 h, the DiO fluorescence signal was visualized via fluorescence microscopy.

To induce phenotypic alterations in M0 and M2, GELNs or FPD‐GELNs (8 µg mL^−1^) were added. After a 24‐h incubation period, the cells were harvested via a cell scraper and stained with the following antibodies: anti‐mouse F4/80 (BioLegend, 123109, CA, USA), anti‐mouse CD86 (BioLegend, 105011), and anti‐mouse CD206 (BioLegend, 141703). For intracellular staining of CD206, the cells were fixed and permeabilized via a fixation/permeabilization kit (BD Biosciences, 562574, NJ, USA). Finally, the phenotypic changes of macrophages were analyzed via FCM

Concurrently, RT‐qPCR analysis was performed to evaluate the relative mRNA expression levels of the macrophage markers CD86, CD206, iNOS, Arg‐1, IL‐6, IL‐12, TNF‐α, and TGF‐β, following the previously described procedures.

### Subcutaneous Tumor Model of Mouse RCC

Six‐week‐old female BALB/c mice were subcutaneously inoculated with 200 µL of RenCa tumor cell suspension at a concentration of 5 × 10^6^ cells mL^−1^. Once the tumor volume reached ≈50 mm^3^, the mice were randomly assigned to 6 groups: Control, Su, GELNs, GELNs/Su, FPD‐GELNs, and FPD‐GELNs/Su. The Su dose was maintained at 2 mg kg^−1^ dose^−1^. The mice received nanoformulations (i.v.) every 3 days for a total of 7 doses over a 21‐day treatment period. During this period, body weight and tumor volume were monitored regularly, and tumor growth curves were plotted. At the end of the experiment, the mice were euthanized, and the tumors were excised and fixed in 4% paraformaldehyde for subsequent paraffin or frozen sectioning. Paraffin sections were subjected to immunohistochemical (IHC) staining for Ki67 and CD34, and IF staining for CD8 and IFN‐γ. Frozen sections were subjected to TUNEL staining.

To obtain a single‐cell suspension from tumor tissues, the tumors were minced and incubated in RPMI‐1640 medium containing hyaluronidase (1 mg mL^−1^; Biofroxx, Germany), type I collagenase (1 mg mL^−1^; Biofroxx), and DNase I (100 µg mL^−1^; Biofroxx) at 37 °C with continuous stirring for 30 min. Undigested tumor fragments were removed by filtration through a 200‐mesh sieve, and red blood cells were lysed via red blood cell lysis buffer (Biosharp) for 5 min on ice. The resulting single‐cell suspensions were collected and stained with the following antibodies: anti‐mouse F4/80, anti‐mouse CD86, anti‐mouse CD206, anti‐mouse CD4 (BioLegend, 100407), and anti‐mouse CD8 (BioLegend, 100711). Finally, the samples were analyzed by FCM.

### The Pharmacokinetics of Su

Pharmacokinetic studies of Su were conducted in 6‐week‐old RenCa tumor‐bearing mice following intravenous administration of Su or FPD‐GELNs/Su at a dose of 2 mg kg^−1^. Blood and tumor samples were collected at predetermined time points (n = 3 per time point). Plasma and tumor homogenates were processed using a protein precipitation method, and the resulting supernatants were evaporated under vacuum at 45 °C. The residues were reconstituted with mobile phase and analyzed by an Agilent 1290 Infinity II UHPLC system equipped with a Kinetex C18 column (50 mm × 2.1 mm, 2.6 µm). The concentrations of Su in plasma and tumor tissues were quantified. Calibration curves were constructed using Su spiked into blank plasma or tumor homogenates to ensure matrix‐matched validation. The mobile phase consisted of solvent A (0.1% formic acid in water) and solvent B (acetonitrile), and gradient elution was performed as detailed in Table S20 (Supporting Information). The flow rate was maintained at 0.4 mL min^−1^, the column temperature was held at 40 °C, and the injection volume was set at 2 µL. All procedures were carried out under minimal light exposure to minimize potential photodegradation. Peak area integration for Su quantification was performed using SCIEX OS software (SCIEX, MA, USA).

### Mouse Model of RCC Lung Metastasis

To evaluate the efficacy of various nanoformulations in inhibiting the lung metastasis of RCC in mice, a mouse model of RCC lung metastasis was established. Specifically, BALB/c mice were intravenously injected with 10^5^ RenCa cells per mouse. Two weeks postinjection, each mouse received a single intravenous dose of GELNs/DiR or FPD‐GELNs/DiR (20 mg kg^−1^). The ability of FPD to target lung metastatic nodules was assessed via an IVIS at multiple time points. Frozen sections were prepared to observe the DiR fluorescence signal.

Additionally, a cohort of RCC lung metastasis models was established in mice. Two weeks after model establishment, each treatment group received intravenous administration of the nanoformulation every 3 days, with a consistent Su dose of 2 mg kg^−1^ dose^−1^. Following 6 administrations, the animals were euthanized, and their lung tissues were harvested for analysis. The number of pulmonary metastatic nodules was meticulously recorded, photographic documentation was obtained, and H&E staining was performed to evaluate the extent of the metastatic lesions.

### Statistical Analysis

Statistical analysis was conducted using GraphPad Prism 10.3 (CA, USA). Data were expressed as mean ± SD. The sample size (n) for each experiment was specified in the corresponding figure legend. Comparisons between two groups were performed using an unpaired Student's *t* test, while multiple‐group comparisons were carried out using one‐way or two‐way ANOVA followed by Tukey's multiple comparison test. Statistical significance was denoted as ^*^ *p* < 0.05, ^**^ *p* < 0.01, and ^***^ *p* < 0.001.

### Animal Ethics Approval

The animal experiments adhered to the guidelines of Reporting of In Vivo Experiments (ARRIVE). Animal studies were approved by the Animal Care and Use Committee of Chongqing Medical University (approval number: IACUC‐CQMU‐2023‐12008).

## Conflict of Interest

The authors declare no conflict of interest.

## Author Contributions

X.H.Y., H.D.X., L.S.X., and Y.L. contributed equally to this work. X.H.Y., H.D.X., L.S.X., and Y.L. designed and conducted the experiments, drafted the manuscript, and prepared the figures. X.H.Y. supervised the execution of the research activities. L.J.W. provided statistical guidance. B.Y.Y. reviewed the manuscript and contributed to its final revision. Z.G.Z. oversaw and coordinated the strategic planning and implementation of research activities. T.W. facilitated the coordination of research activities and offered expert methodological guidance and financial support. J.L. established the research objectives and framework for the project, and also provide essential financial support. All authors have thoroughly reviewed the manuscript.

## Supporting information



Supporting Information

## Data Availability

The data that support the findings of this study are available from the corresponding author upon reasonable request.
