# Supplementary material for: Folic Acid‐Modified Ginger‐Derived Exosome‐Like Nanoparticles Co‐Delivering Sunitinib Suppress Renal Cell Carcinoma via PI3K‐Akt Pathway Inhibition, P‐gp Downregulation, and Macrophage Reprogramming
Source: Adv Sci (Weinh). 2025 Nov 17;13(6):e12563. doi: 10.1002/advs.202512563 (PMC12866827; doi:10.1002/advs.202512563)
Supplement: Supplementary file 1 — Supporting Information [file ADVS-13-e12563-s001.docx]

****Folic Acid-modified Ginger-derived Exosome-like Nanoparticles Co-delivering Sunitinib Suppress Renal Cell Carcinoma via PI3K-Akt Pathway Inhibition, P-gp Downregulation, and Macrophage Reprogramming****

*Haoyu Xu, Daixing Hu, Shixue Liu, Lei Yang, Junwu Li, Yuanyuan Bai, Guozhi Zhao*, Wei Tang* and Li Jiang**

H. Xu, S. Liu, L. Yang, J. Li, Y. Bai, G. Zhao, W. Tang, L. Jiang

Department of Urology

The First Affiliated Hospital of Chongqing Medical University

Chongqing 400016, China

1. mail: [2h5906@hospital.cqmu.edu.cn](mailto:201522@hospital.cqmu.edu.cn); [201522@hospital.cqmu.edu.cn](mailto:201522@hospital.cqmu.edu.cn); [204215@hospital.cqmu.edu.cn](mailto:201522@hospital.cqmu.edu.cn)

H. Xu

Department of Urology

The Affiliated Yongchuan Hospital of Chongqing Medical University

Chongqing 402160, China

D. Hu

Department of Breast and Thyroid Surgery

The Second Affiliated Hospital of Chongqing Medical University

Chongqing 400010, China

S. Liu

Department of Urology

The Shapingba Hospital, Chongqing University (People’s Hospital of Shapingba District)

Chongqing 400030, China


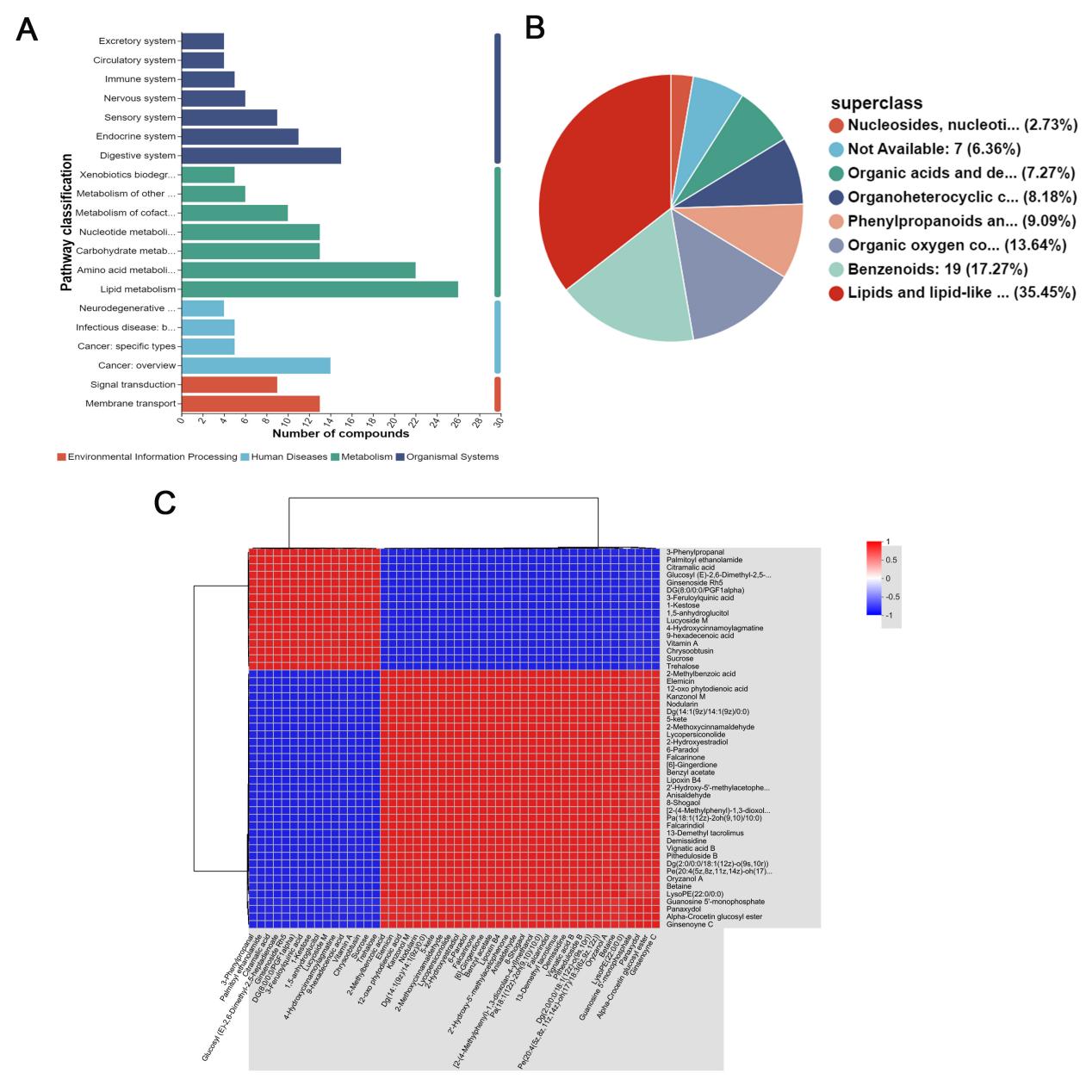


**Figure S1.** Metabolomics of GELNs. **A)** KEGG pathway enrichment analysis of GELN metabolites. **B)** HMDB compound classification of differential metabolites between GELNs2 and GELNs1. **C)** Correlation analysis of differential metabolites between GELNs2 and GELNs1.


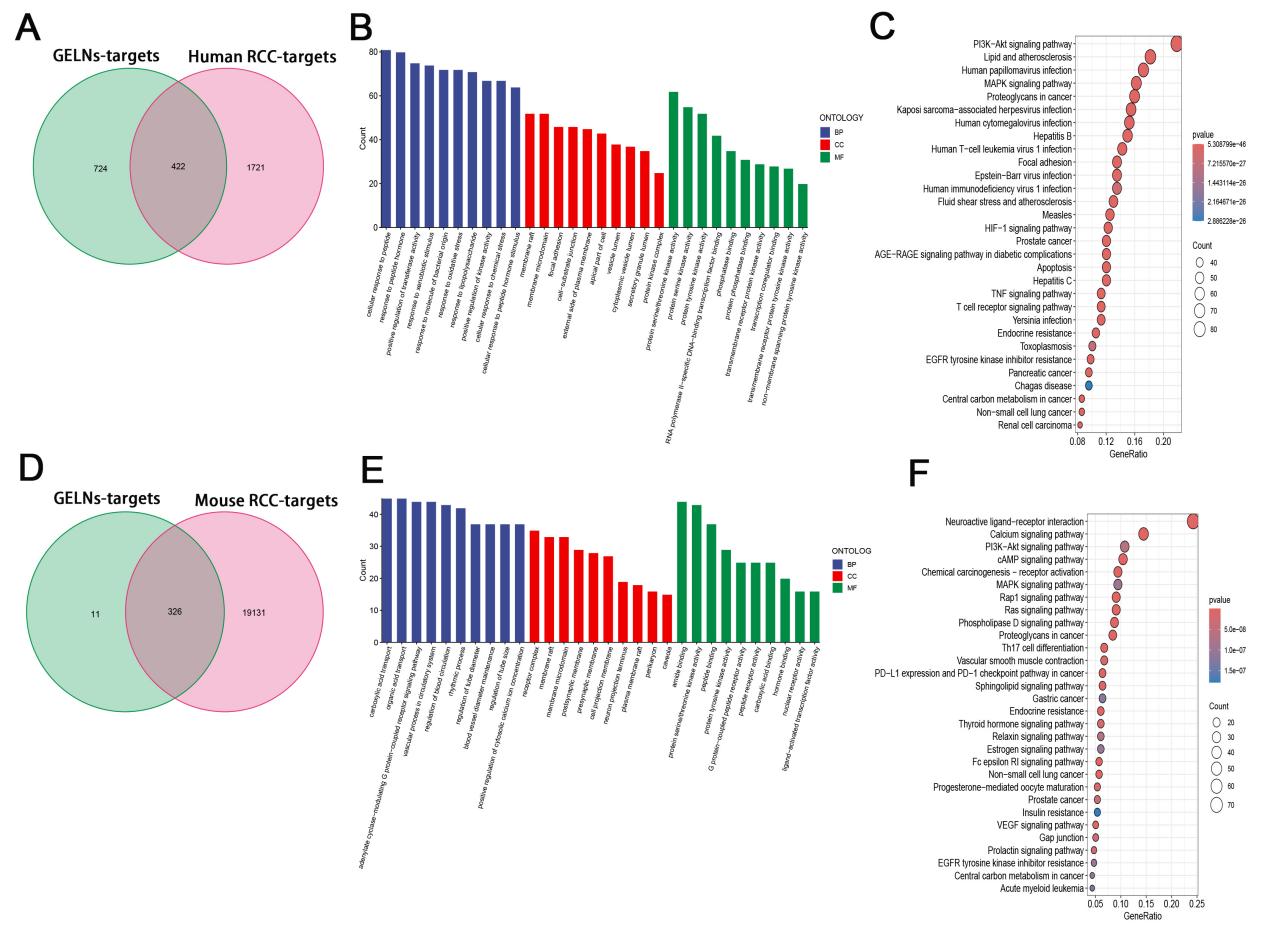


**Figure S2.** Investigation into the initial mechanisms of GELNs in regulating RCC. **A)** Venn diagram illustrating the intersection of GELN action targets and human RCC-related targets. **B)** GO annotation and **C)** KEGG pathway enrichment analysis of the 422 GELN-human RCC targets. **D)** Venn diagram illustrating the intersection of GELN action targets and mouse RCC-related targets. **E)** GO annotation and **F)** KEGG pathway enrichment analysis of the 326 GELN-mouse RCC targets.


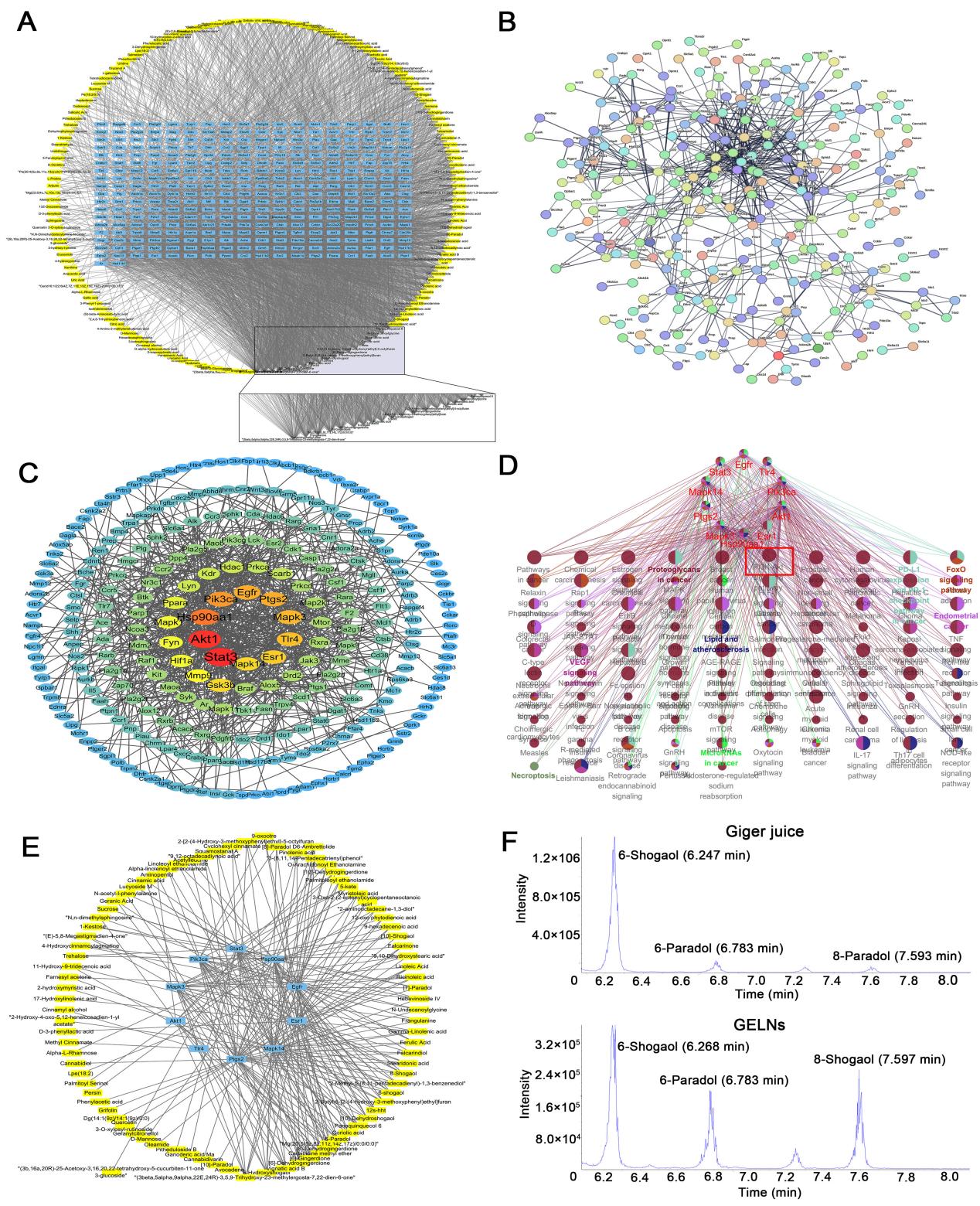


**Figure S3.** Exploration of the mechanism by which GELNs regulate mouse RCC. **A)** Drug regulatory network of GELNs acting on mouse RCC. **B)** PPI network of GELN-related targets in mouse RCC, constructed via the STRING database. **C)** Gene-gene relationship network, sorted by degree centrality, constructed via Cytoscape. **D)** Gene-pathway connection map, sorted by degree centrality, generated via the ClueGO and CluePedia plugins in Cytoscape. **E)** Drug regulatory network of compounds targeting mouse CHGs. **F)** Quantitative analysis of 6-Shogaol, 8-Shogaol, and 6-Paradol in ginger juice or GELNs was performed using HPLC (n = 3 independent experiments).


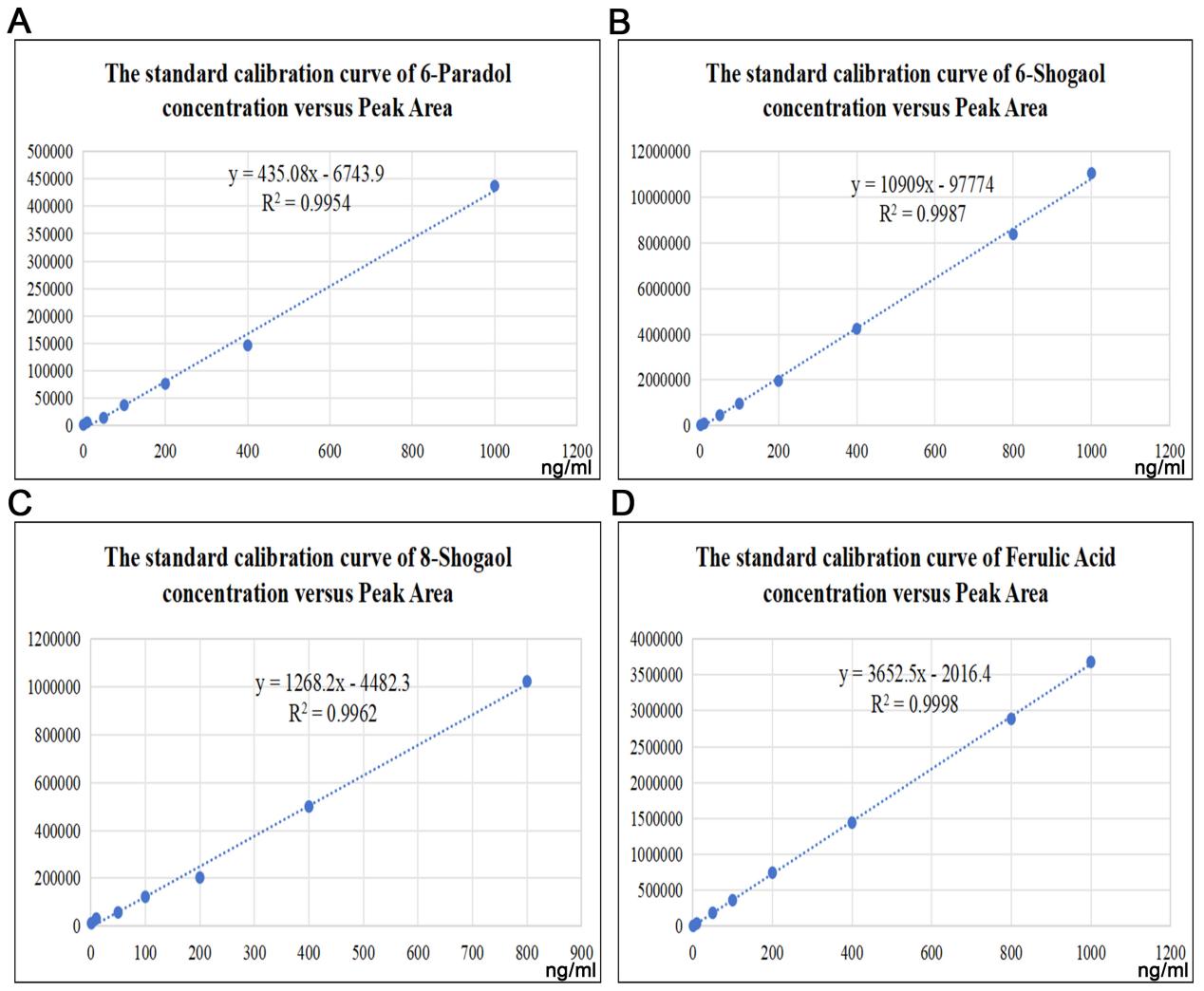


**Figure S4.** Standard calibration curves of **A)** 6-Paradol, **B)** 6-Shogaol, **C)** 8-Shogaol, and **D)** Ferulic Acid, plotting concentration versus peak area.


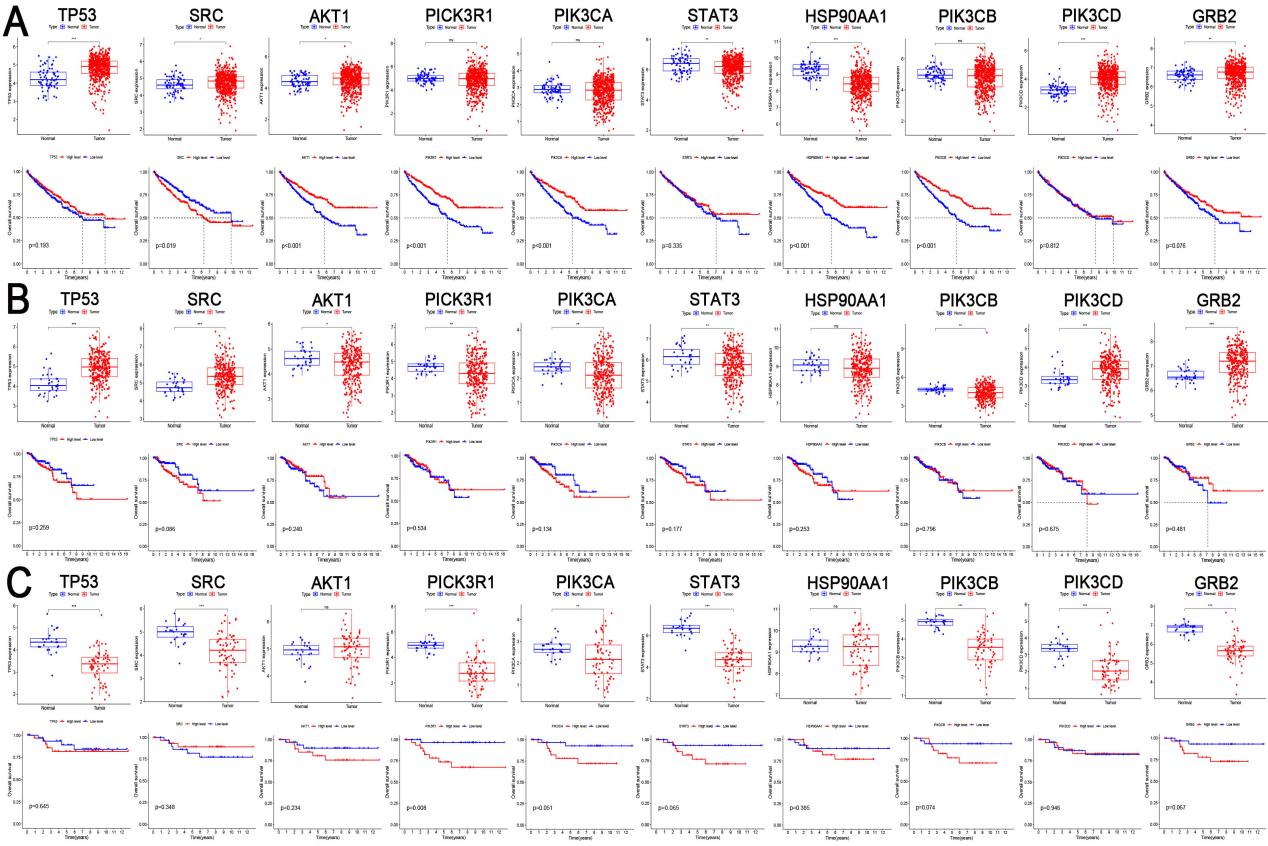


**Figure S5.** Differential expression and survival analysis of CHGs in tumor versus adjacent nonTumor tissues across **A)** TCGA-KIRC, **B)** TCGA-KIRP, and **C)** TCGA-KICH cohorts. **p* < 0.05, ***p* < 0.01, ****p* < 0.001. ns, not significancant.


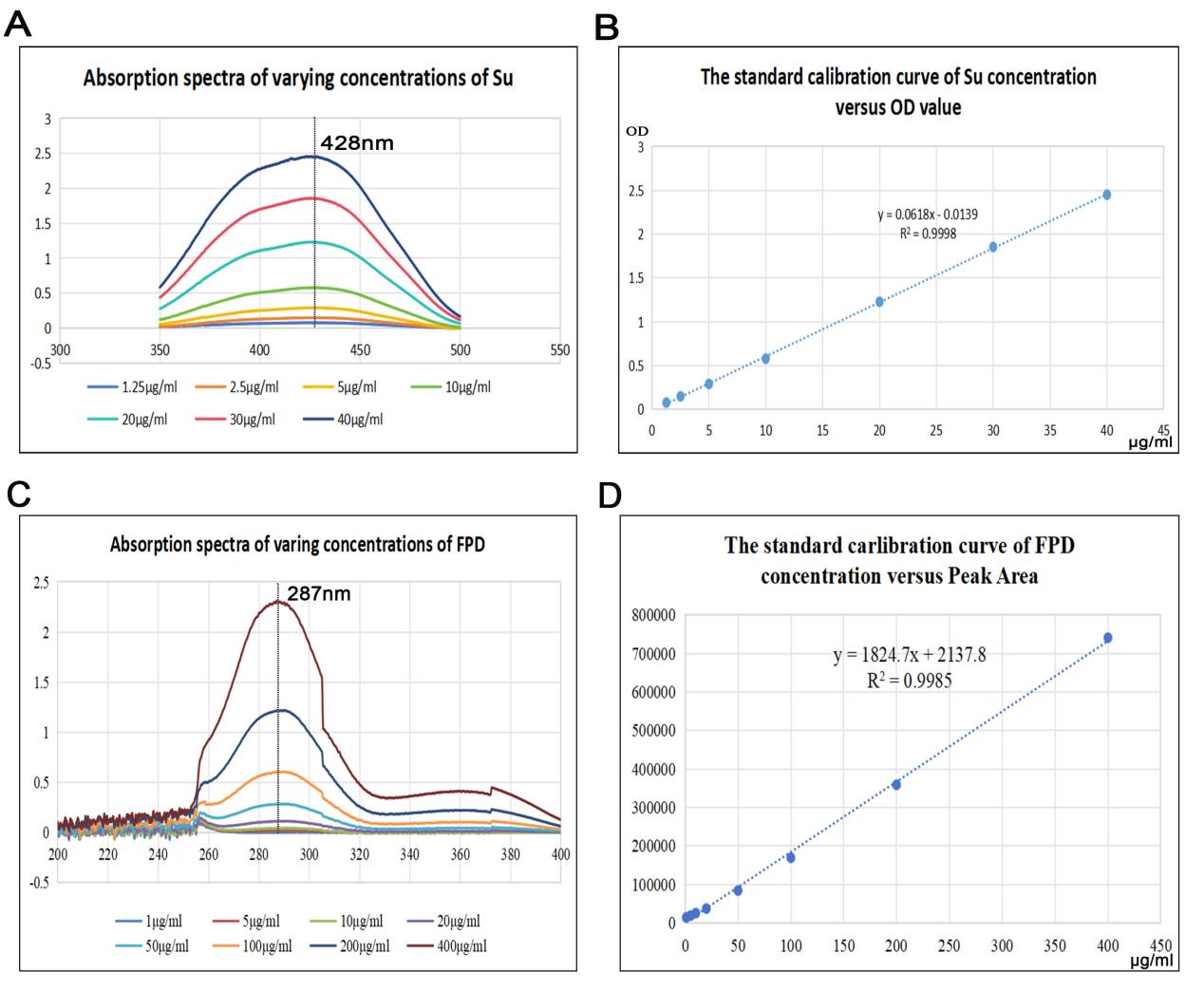


**Figure S6.** Standard calibration curves for Su concentration versus absorbance and FPD concentration versus peak area. **A)** UV absorption spectra of Su at various concentrations within the wavelength range of 350-500 nm. **B)** Standard calibration curve of the Su concentration-absorbance at 428 nm. **C)** UV absorption spectra of FPD at various concentrations within the wavelength range of 200-400 nm. **D)** Standard calibration curve of the FPD concentration-peak area at 287 nm.


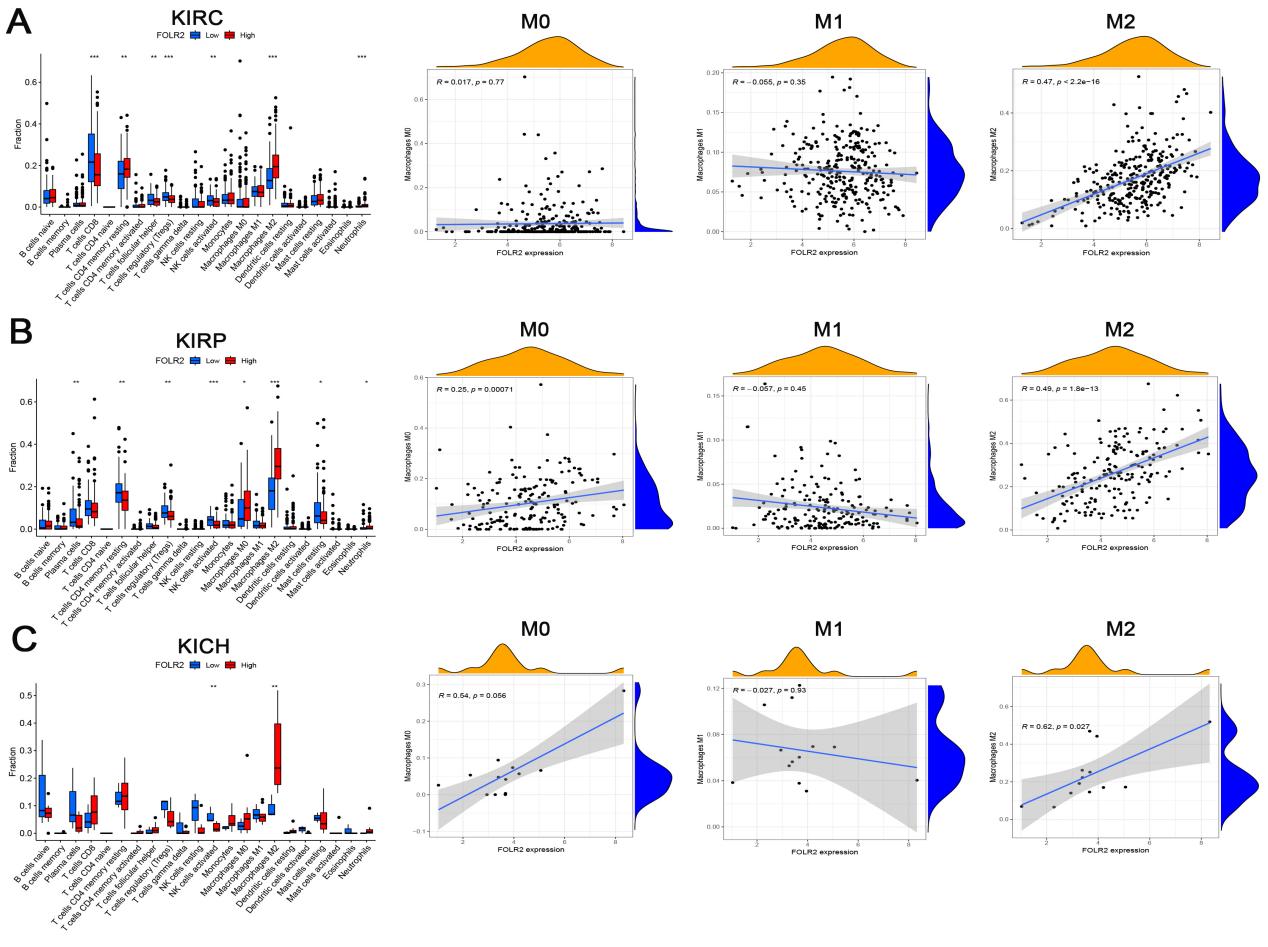


**Figure S7.** Differential infiltration of 22 immune cell subsets between the high and low FOLR2 expression groups in the **A)** TCGA-KIRC, **B)** TCGA-KIRP, and **C)** TCGA-KICH cohort, and correlation analysis between FOLR2 and macrophages. **p* < 0.05, ***p* < 0.01 and ****p* < 0.001.


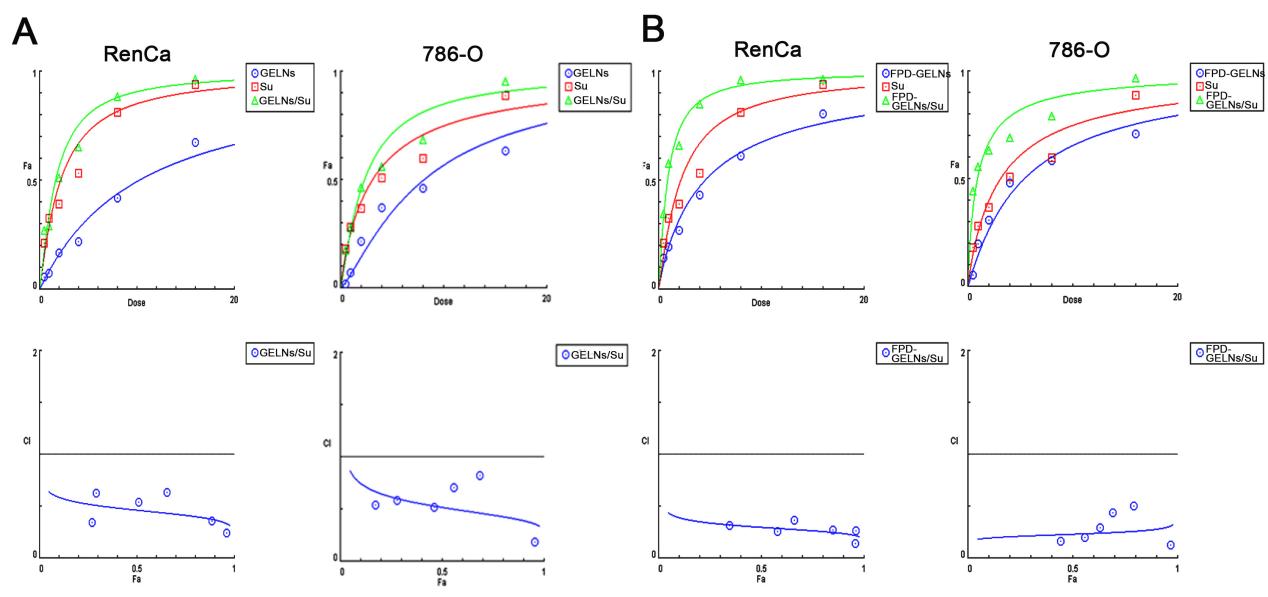


**Figure S8.** Combination effect curves of **A)** GELNs and Su on GELNs/Su, alongside with **B)** FPD-GELNs and Su on FPD-GELNs/Su at Su concentrations ranging from 0.5 to 16 μM.


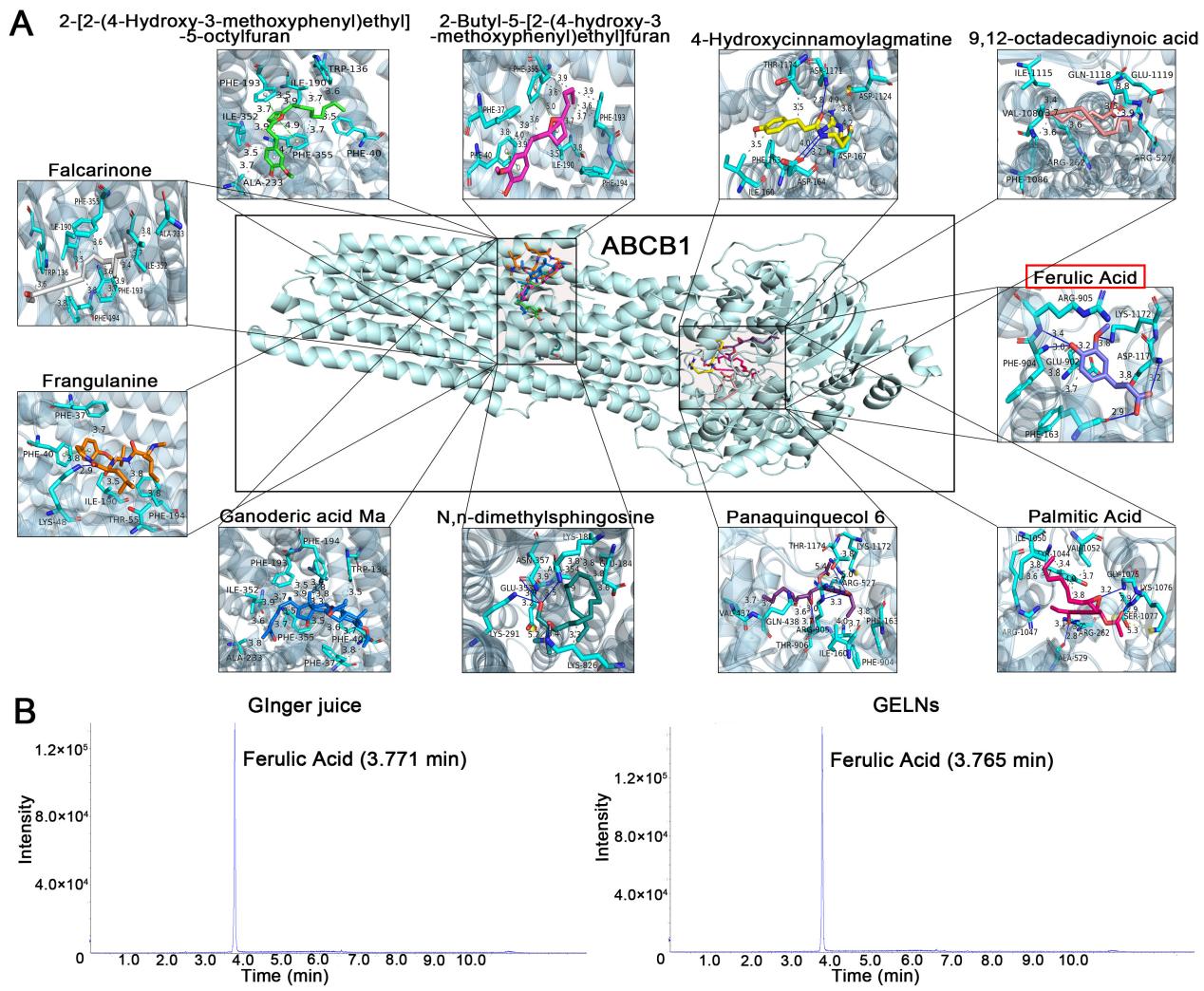


**Figure S9.** **A)** Molecular docking of 11 compounds from GELNs with the target protein ABCB1. The blue lines represent hydrogen bonds, the gray dashed lines represent hydrophobic interactions, the light green dashed lines represent π-π stacking (parallel), the dark green dashed lines represent π-π stacking (perpendicular), and the yellow dashed lines represent salt bridges. **B)** Quantitative analysis of ferulic acid in ginger juice or GELNs was performed using HPLC (n = 3 indepengdent experiments).


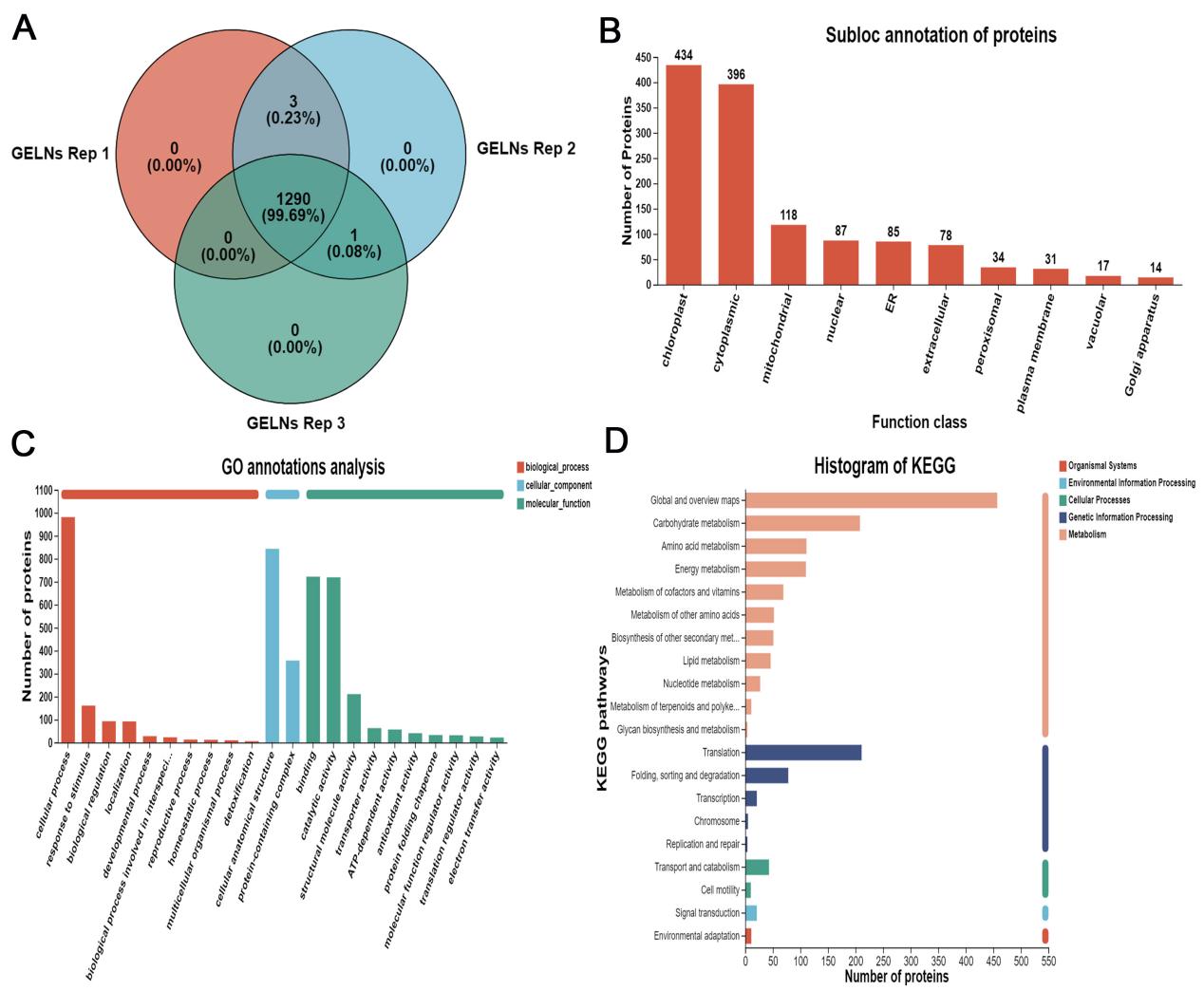


**Figure S10.** Proteomic profiling of GELNs. **A)** Venn diagram depicting overlapping proteins identified across three independent GELNs samples (n = 3 independent experiments). **B)** Subcellular localization distribution of GELNs-derived proteins. **C)** GO functional annotation and **D)** KEGG pathway enrichment analysis of GELNs proteins.


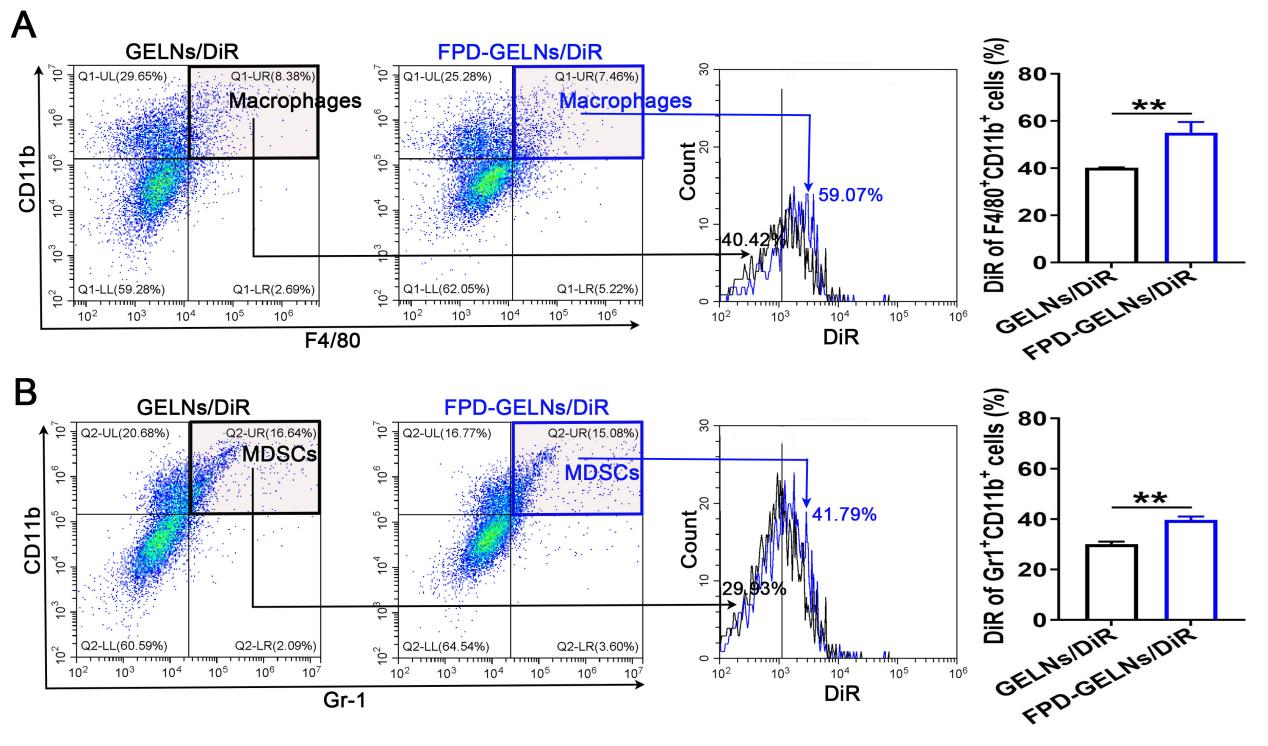


**Figure S11.** FCM analysis of DiR fluorescence proportions in **A)** TAMs (F4/80^+^CD11b^+^) and **B)** MDSCs (Gr1^+^CD11b^+^) from RenCa tumor single-cell suspensions after a single tail vein injection of GELNs/DiR or FPD-GELNs/DiR (n = 3 independent experiments). Data are presented as mean ± SD. ***p* < 0.01 (unpaired Student’s t test).


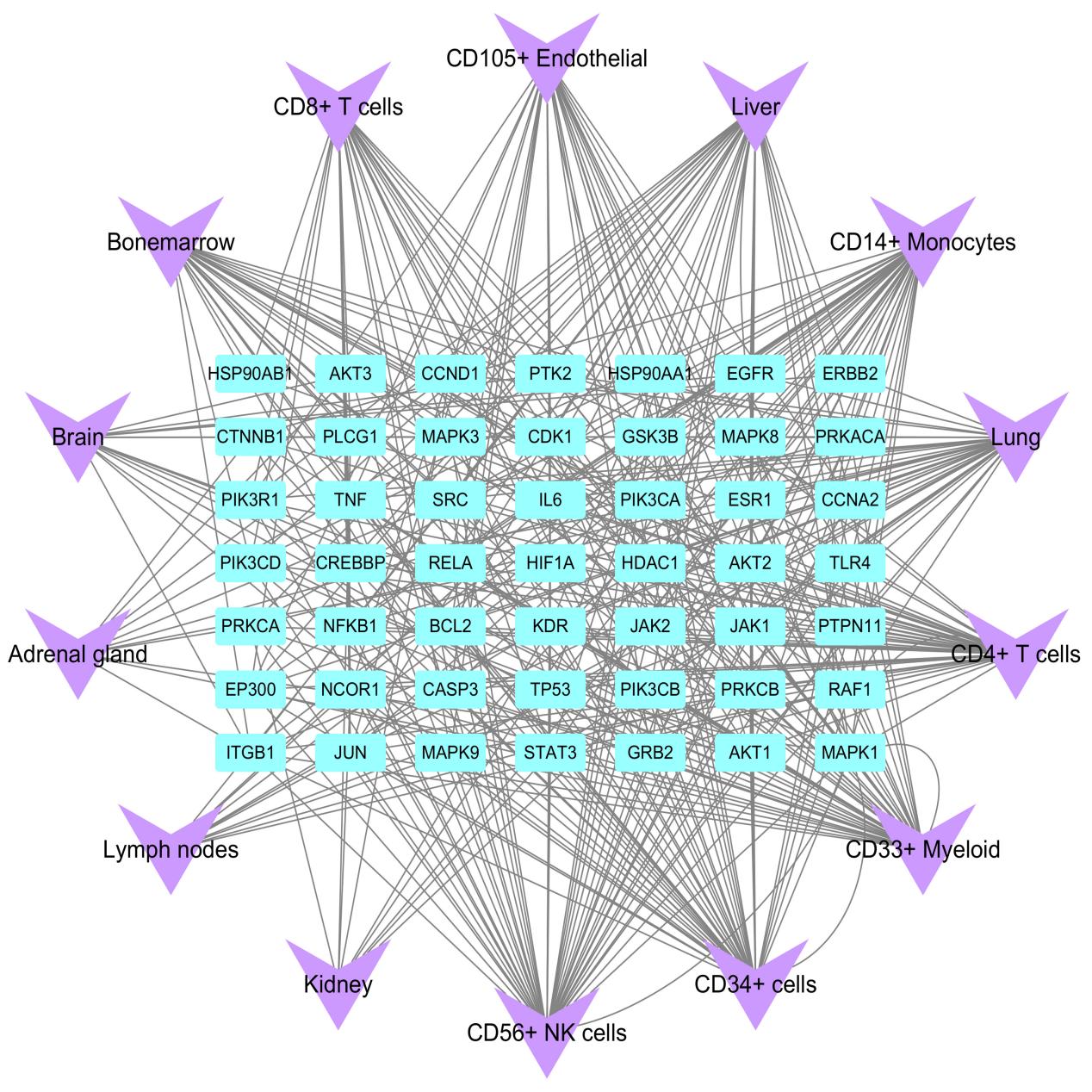


**Figure S12.** Tissue-target network of the top 50 targets ranked by degree. The purple arrows represent tissues or immune cells, the light blue quadrilaterals represent target genes, and the black lines indicate connections between tissues/immune cells and target genes.

**Table S1.** Metabolite profiles of GELNs

| **Metabolite name** | **Mass** | **Formula** | ***m/z*** |
| --- | --- | --- | --- |
| 4-hydroxyproline | 131.0582432 | C5H9NO3 | 96.04459939 |
| 4-Amino-2-methylenebutanoic acid | 115.0633285 | C5H9NO2 | 98.06021987 |
| 2-Furanmethanol | 98.03677944 | C5H6O2 | 99.04421515 |
| Cis,cis-muconic acid | 142.0266087 | C6H6O4 | 143.0335744 |
| 5-Hydroxymethyl-2-Furaldehyde | 126.0316941 | C6H6O3 | 127.0387733 |
| 2-Pyrrolidinone | 85.05276385 | C4H7NO | 86.0603587 |
| (E)-2-Penten-1-ol | 86.07316494 | C5H10O | 104.107063 |
| Triethanolamine | 149.1051934 | C6H15NO3 | 150.1121135 |
| Beta-D-Glucosamine | 179.0793725 | C6H13NO5 | 180.0861701 |
| Quercetin 3-O-xylosyl-rutinoside | 742.1956437 | C32H38O20 | 765.178333 |
| Adenine | 135.0544952 | C5H5N5 | 136.0615013 |
| Guanine | 151.0494098 | C5H5N5O | 152.0563269 |
| Cytarabine | 243.0855205 | C9H13N3O5 | 244.0920878 |
| L-Leucine | 131.0946287 | C6H13NO2 | 132.1017101 |
| 4-Guanidinobutanoic Acid | 145.0851266 | C5H11N3O2 | 146.0920668 |
| L-Proline | 115.0633285 | C5H9NO2 | 98.06024352 |
| Phloroglucinol | 126.0316941 | C6H6O3 | 127.0387932 |
| Netilmicin | 475.3005987 | C21H41N5O7 | 476.305601 |
| D-Ornithine | 132.0898776 | C5H12N2O2 | 265.1890408 |
| Pectenotoxin 1 | 874.4714716 | C47H70O15 | 929.4887442 |
| 10-hydroxydec-2-enoic acid | 186.1255944 | C10H18O3 | 187.1325325 |
| 5-Hydroxy-p-mentha-6,8-dien-2-one | 166.0993797 | C10H14O2 | 184.13288 |
| O-Xylene | 106.0782503 | C8H10 | 107.0856455 |
| 3-Phenylpropanal | 134.0731649 | C9H10O | 135.0802422 |
| 1,5-anhydroglucitol | 164.068473494;612.205420459 | C6H12O5 | 165.0754597 |
| Ginsenoside Rh5 | 652.4550336 | C37H64O9 | 670.4849588 |
| (E)-10-Hydroxy-8-decenoic acid | 186.1255944 | C10H18O3 | 187.1324782 |
| Menthol | 156.1514153 | C10H20O | 161.1321519 |
| O-Arachidonoyl Ethanolamine | 347.2824294 | C22H37NO2 | 352.258593 |
| (S)-10,16-Dihydroxyhexadecanoic acid | 288.2300595 | C16H32O4 | 306.2630699 |
| Curcumol | 236.17763 | C15H24O2 | 237.1842758 |
| Jasmone | 164.1201151 | C11H16O | 165.1270812 |
| Curdione | 236.17763 | C15H24O2 | 219.173825 |
| Porson | 386.1729386 | C22H26O6 | 404.2058629 |
| Dehydrocostus lactone | 230.1306798 | C15H18O2 | 248.1638603 |
| (E)-5,8-Megastigmadien-4-one | 192.1514153 | C13H20O | 193.1583222 |
| Triphenylphosphine oxide | - | C18H15OP | 279.092557 |
| Panaxydol | 260.17763 | C17H24O2 | 261.1842111 |
| 8-Deoxy-11-hydroxy-13-chlorogrosheimin | 298.0971868 | C15H19ClO4 | 263.0809045 |
| Indene | 116.0626003 | C9H8 | 117.0698714 |
| 4-Methylbenzoic acid | 136.0524295 | C8H8O2 | 137.0594708 |
| Ginsenoyne A | 258.1619799 | C17H22O2 | 259.1686169 |
| (3beta,5beta,8beta,22E,24xi)-Ergosta-6,22-diene-3,5,8-triol | 430.3446953 | C28H46O3 | 430.3452403 |
| 2-phenylacetamide | 135.0684139 | C8H9NO | 118.0650536 |
| P-Tolualdehyde | 120.057514878;360.172544633 | C8H8O | 121.0646735 |
| (+/-)-Furaneol | 128.0473441 | C6H8O3 | 129.0544189 |
| Dihydroceramide | 329.2929941 | C19H39NO3 | 362.3253742 |
| Hexadecasphingosine | 271.2511293 | C16H33NO2 | 272.2575974 |
| Vitamin A | 286.2296656 | C20H30O | 328.2623274 |
| 9-hexadecenoic acid | 254.2245802 | C16H30O2 | 272.257626 |
| Hydrocinnamic acid | 150.0680796 | C9H10O2 | 151.0750144 |
| Trans-Isoasarone | 208.1099444 | C12H16O3 | 191.1061863 |
| Heptadecanoic acid | 270.2558803 | C17H34O2 | 288.2888488 |
| Precocene I | 190.0993797 | C12H14O2 | 191.1062279 |
| 1,2-Dihydronaphthalene-1,2-diol | 162.0680796 | C10H10O2 | 163.0750063 |
| D6-Ambrettolide | 252.2089301 | C16H28O2 | 270.2420067 |
| 4-Propylphenol | 136.088815 | C9H12O | 119.0854791 |
| L-rhamnofuranose | 164.0684735 | C6H12O5 | 129.0544387 |
| Cinnamaldehyde | 132.0575149 | C9H8O | 133.0646773 |
| Pectachol | 442.2355388 | C26H34O6 | 460.2680815 |
| 3-ketosphingosine | 297.2667794 | C18H35NO2 | 298.2732321 |
| 2-aminooctadecane-1,3-diol | 301.2980795 | C18H39NO2 | 302.3044831 |
| Sphingosine | 299.2824294 | C18H37NO2 | 300.2888772 |
| Margaroylglycine | 327.2773441 | C19H37NO3 | 328.2836848 |
| 1-naphthol | 144.0575149 | C10H8O | 145.0645463 |
| 12-oxo phytodienoic acid | - | C18H28O3 | 293.2102643 |
| 3-Dehydrosphinganine | 299.2824294 | C18H37NO2 | 332.3149539 |
| Muzanzagenin | 442.2719243 | C27H38O5 | 506.2889635 |
| Palmitoyl ethanolamide | 299.2824294 | C18H37NO2 | 300.2888942 |
| S-methoprene | - | C19H34O3 | 311.257298 |
| LysoPC(17:0/0:0) | 509.3481395 | C25H52NO7P | 492.3460571 |
| 7-Hydroxy-6-methyl-2H-1-benzopyran-2-one | 176.0473441 | C10H8O3 | 177.0542908 |
| N,n-dimethylsphingosine | 327.3137296 | C20H41NO2 | 328.3201247 |
| Arachidoyl ethanolamide | 355.3450297 | C22H45NO2 | 356.3513116 |
| [10]-Paradol | 334.250795 | C21H34O3 | 398.2679009 |
| LysoPE(0:0/22:0) | 537.3794397 | C27H56NO7P | 520.3774739 |
| Cinnamic acid | 148.0524295;148.0524295;148.0524295;148.0524295 | C9H8O2 | 131.0490154 |
| 2-Methoxycinnamaldehyde | 162.0680796 | C10H10O2 | 163.0750107 |
| 6-Paradol | 278.1881947 | C17H26O3 | 261.1843634 |
| Rishitin | 222.1619799 | C14H22O2 | 277.1793723 |
| Gamma-Linolenic acid | 278.2245802 | C18H30O2 | 243.2101015 |
| 22-Hydroxydocosanoic acid | 356.3290453 | C22H44O3 | 374.3618174 |
| 3-Oxo-2-(2-entenyl)cyclopentaneoctanoic acid | 294.2194948 | C18H30O3 | 349.2363909 |
| Methyl 4-Methoxycinnamate | - | C11H12O3 | 193.0855844 |
| LysoPE(0:0/16:0) | 453.2855393 | C21H44NO7P | 454.2916693 |
| Cinnamyl alcohol | 134.073164942;134.073164942;134.073164942;134.073164942 | C9H10O | 117.0698604 |
| 2-naphthol | 144.0575149 | C10H8O | 145.0645363 |
| Tridecanoic acid | 214.1932801 | C13H26O2 | 197.1895884 |
| Cannabidiol | 314.2245802 | C21H30O2 | 315.2310855 |
| [10]-Shogaol | 332.2351449 | C21H32O3 | 333.2415465 |
| Elemicin | 208.1099444 | C12H16O3 | 191.1062301 |
| 4-amino-4-deoxychorismate | 225.0637225 | C10H11NO5 | 190.0494609 |
| Octyl hydrogen phthalate | 278.1518092 | C16H22O4 | 279.1583763 |
| Palmitoleoyl ethanolamide | 297.2667794 | C18H35NO2 | 298.2732112 |
| Levomenol | 222.1983655 | C15H26O | 205.1946481 |
| Decylubiquinone | 322.2144094 | C19H30O4 | 345.2052021 |
| Neocnidilide | 194.13067982;194.13067982;194.13067982 | C12H18O2 | 195.1376074 |
| 3,7-Dimethyl-2E,6-octadienyl acetate | - | C12H20O2 | 197.1532307 |
| Ketoisophorone | 152.0837296 | C9H12O2 | 153.090706 |
| 4-hydroxynonenal | 156.1150298 | C9H16O2 | 121.1011114 |
| Sebacic Acid | 202.1205091 | C10H18O4 | 203.1273462 |
| 4-Vinylcyclohexene | 108.0939004 | C8H12 | 109.1012467 |
| Linoleoyl ethanolamide | 323.2824294 | C20H37NO2 | 324.2888648 |
| Arbutin | 272.0896029 | C12H16O7 | 273.0961863 |
| Carvone | 150.10446507;150.104465071 | C10H14O | 151.1114365 |
| Anacardic acid | 344.2351449 | C22H32O3 | 377.2678453 |
| 6-Hydroxyshogaol | 292.1674593 | C17H24O4 | 293.173826 |
| Ceanothenic acid | 454.3083098 | C29H42O4 | 472.3409946 |
| P-Mentha-1,3,5,8-tetraene | 132.0939004 | C10H12 | 133.1010132 |
| 2-[2-(4-Hydroxy-3-methoxyphenyl)ethyl]-5-octylfuran | 330.2194948 | C21H30O3 | 331.225853 |
| Limonene oxide | 152.1201151 | C10H16O | 170.1535709 |
| 12-aminododecanoic acid | - | C12H25NO2 | 198.184825 |
| Diisopropyl phthalate | - | C14H18O4 | 233.1165843 |
| 4-Hydroxy-2,6,6-trimethyl-3-oxo-1,4-cyclohexadiene-1-carboxaldehyde | 180.0786443 | C10H12O3 | 145.0645287 |
| (3R,3aR,7aS)-3-Butylhexahydro-1(3H)-isobenzofuranone | 196.1463299 | C12H20O2 | 161.1321443 |
| 6-shogaol | - | C17H24O3 | 277.1792331 |
| Urobilinogen | 590.3104351 | C33H42N4O6 | 654.3303824 |
| Alantolactone | 232.1463299 | C15H20O2 | 233.1530124 |
| Corticosterone | 346.2144094 | C21H30O4 | 347.2207837 |
| Camphor | 152.120115134;152.120115134 | C10H16O | 170.1535579 |
| 11-Aminoundecanoic Acid | - | C11H23NO2 | 184.1692281 |
| Dihydro-alpha-ionone | 194.1670653 | C13H22O | 212.2003289 |
| Oleamide | 281.2718647 | C18H35NO | 282.2783681 |
| Nodularin | 824.4432402 | C41H60N8O10 | 842.4731392 |
| 2,4,5,7alpha-Tetrahydro-1,4,4,7a-tetramethyl-1H-inden-2-ol | 192.1514153 | C13H20O | 193.1583383 |
| Alpha-Ionone | 192.1514153 | C13H20O | 175.1477884 |
| 2,5-Heptadien-1-ol | 112.088815 | C7H12O | 95.08578273 |
| Ethylbenzene | 106.0782503 | C8H10 | 107.0856233 |
| Phenyl acetate | 136.0524295 | C8H8O2 | 137.0595048 |
| Citral | 152.1201151 | C10H16O | 153.1270639 |
| Panaxytriol | 278.1881947 | C17H26O3 | 279.1948237 |
| Gingerol | 294.1831093 | C17H26O4 | 295.1895894 |
| 7alpha-Hydroxy-3-oxo-4-cholestenoate | 430.3083098 | C27H42O4 | 431.3146156 |
| 4-Vinylguaiacol | 150.0680796 | C9H10O2 | 151.0750674 |
| (Z)-3-Oxo-2-(2-pentenyl)-1-cyclopenteneacetic acid | 208.1099444 | C12H16O3 | 191.1062678 |
| 9-oxootre | - | C18H28O3 | 293.2102345 |
| Cyanidin 3-(2G-glucosylrutinoside) | 757.2191188 | C33H41O20+ | 758.2198476 |
| Dibutyl phthalate | 278.1518092 | C16H22O4 | 279.1584912 |
| Frangulanine | 500.3362559 | C28H44N4O4 | 518.37492 |
| Fa(18:3+1o) | - | C18H30O3 | 295.2260305 |
| Dg(14:1(9z)/15:0/0:0) | 524.444075 | C32H60O5 | 563.4084823 |
| Cer(d16:1/22:6(4Z,7Z,11E,13Z,15E,19Z)-2OH(10S,17)) | 613.4706241 | C38H63NO5 | 596.4664122 |
| 13Z-Docosenamide | - | C22H43NO | 338.3407981 |
| N-acetyl-l-phenylalanine | 207.0895433 | C11H13NO3 | 240.1221934 |
| 3-hydroxy-l-proline | 131.0582432 | C5H9NO3 | 96.04458098 |
| Larixinic acid | 126.0316941 | C6H6O3 | 127.0387525 |
| Pyrrolidine | 71.07349929 | C4H9N | 72.08129658 |
| 3-Phenyl-1-propanol | 136.088815 | C9H12O | 178.1222077 |
| Choline | 104.1075391 | C5H14NO+ | 104.107139 |
| (3beta,5alpha,9alpha,22E,24R)-3,5,9-Trihydroxy-23-methylergosta-7,22-dien-6-one | 458.33961 | C29H46O4 | 481.3303868 |
| Bis(2-ethylhexyl) phthalate | 390.2770097 | C24H38O4 | 391.2833658 |
| Phthalic Acid | 166.0266087 | C8H6O4 | 167.0335748 |
| Salicylic Acid | 138.0316941 | C7H6O3 | 121.0283452 |
| Persin | 380.2926598 | C23H40O4 | 403.2834141 |
| (2alpha,3alpha,5alpha,22R,23R)-2,3,22,23-Tetrahydroxy-25-methylergost-24(28)en-6-one | 476.3501746 | C29H48O5 | 459.3459313 |
| [8]-Paradol | 306.2194948 | C19H30O3 | 307.226008 |
| Geranylcitronellol | 292.2766158 | C20H36O | 310.3095741 |
| Nootkatone | 218.1670653 | C15H22O | 219.1738106 |
| P-Mentha-1,3,8-triene | 134.1095504 | C10H14 | 135.1166346 |
| Pe(15:0/16:1(9z)) | 675.4839052 | C36H70NO8P | 708.5112419 |
| 2-Methylpropanamine | 73.08914936 | C4H11N | 74.09695878 |
| 13-Demethyl tacrolimus | 789.4663266 | C43H67NO12 | 844.4889342 |
| Dg(14:1(9z)/14:1(9z)/0:0) | 508.4127749 | C31H56O5 | 572.4296471 |
| Erucamide | - | C22H43NO | 338.3407882 |
| 2-Polyprenyl-6-methoxyphenol | 260.17763 | C17H24O2 | 261.1842468 |
| Methyl Cinnamate | 162.068079564;162.068079564 | C10H10O2 | 163.0749966 |
| Euscaphic acid | 488.3501746 | C30H48O5 | 493.3299977 |
| LysoPE(22:0/0:0) | 537.3794397 | C27H56NO7P | 570.4141808 |
| 2,2,6,7-Tetramethylbicyclo[4.3.0]nona-1(9),4-diene-7,8-diol | 208.1463299 | C13H20O2 | 209.1531285 |
| Cis-Quinceoxepane | 180.1514153 | C12H20O | 198.1848195 |
| Damascenone | 190.1357652 | C13H18O | 191.1426144 |
| Dodemorph | - | C18H35NO | 563.5498448 |
| Alpha-linolenoyl ethanolamide | 321.2667794 | C20H35NO2 | 339.2996068 |
| Estrane | 246.234751 | C18H30 | 247.2413728 |
| Hexadecanamide | 255.2562147 | C16H33NO | 256.2628194 |
| Aminopentol | 405.3454236 | C22H47NO5 | 438.3779045 |
| 3,4-Dihydrocadalene | 200.1565006 | C15H20 | 201.1633478 |
| Zerumbone | 218.1670653 | C15H22O | 219.1738202 |
| Pc(2:0/18:2(10e,12z)+=o(9)) | 575.3223192 | C28H50NO9P | 576.3307738 |
| Dg(2:0/0:0/18:1(12z)-o(9s,10r)) | 412.282489 | C23H40O6 | 377.2677195 |
| 6-gingerol | 294.18310932;294.183109317 | C17H26O4 | 259.1686035 |
| Alpha-curcumene | 202.172150704;202.172150708 | C15H22 | 203.1790124 |
| Pe(20:4(5z,8z,11z,14z)-oh(17)/18:3(6z,9z,12z)) | 777.4944699 | C43H72NO9P | 816.4569107 |
| Dihydrokavain | 232.1099444 | C14H16O3 | 215.1061937 |
| Farnesyl acetone | - | C18H30O | 263.236293 |
| Isophorone | 138.1044651 | C9H14O | 156.1380062 |
| Lycopersiconolide | 362.2457096 | C22H34O4 | 363.2521731 |
| Panaxynol | 244.1827154 | C17H24O | 277.2155734 |
| 2-Butyl-5-[2-(4-hydroxy-3-methoxyphenyl)ethyl]furan | 274.1568946 | C17H22O3 | 275.1635278 |
| (S)-(-)-Perillyl alcohol | 152.1201151 | C10H16O | 135.116626 |
| Peimine | 431.3399443 | C27H45NO3 | 454.3304029 |
| 1-Butylamine | 73.08914936 | C4H11N | 74.09697067 |
| Coenzyme q2 | 318.1831093 | C19H26O4 | 319.189611 |
| Sclareolide | 250.1932801 | C16H26O2 | 233.1894207 |
| Carvacrol | 150.104465071;454.344695338;150.104465071 | C10H14O | 151.111471 |
| Chavicol | 134.0731649 | C9H10O | 135.0802737 |
| Indole | 117.0578492 | C8H7N | 118.0650954 |
| 2',7-Dihydroxy-4'-methoxy-8-prenylflavan | 340.1674593 | C21H24O4 | 341.1738574 |
| Aldosterone | 360.193674 | C21H28O5 | 361.2002232 |
| [10]-Dehydroshogaol | 330.2194948 | C21H30O3 | 348.2522095 |
| Xestoaminol C | 229.2405646 | C14H31NO | 252.2315545 |
| Linalool | 154.1357652 | C10H18O | 155.1426942 |
| Linoleic Acid | 280.2402303 | C18H32O2 | 245.2257673 |
| Pe(18:1/0:0) | - | C23H46NO7P | 480.3073916 |
| Monoisobutyl phthalate | 222.0892089 | C12H14O4 | 205.0854775 |
| 2,5-dihydroxybenzaldehyde | 138.0316941 | C7H6O3 | 121.0283383 |
| Lipoxin B4 | 352.2249741 | C20H32O5 | 335.2207759 |
| 2'-Hydroxy-5'-methylacetophenone | 150.0680796 | C9H10O2 | 151.0750791 |
| Methyl benzoate | 136.0524295 | C8H8O2 | 137.0595304 |
| 9,12-octadecadiynoic acid | 276.2089301 | C18H28O2 | 277.2155656 |
| Isolinderenolide | 334.250795 | C21H34O3 | 317.2465606 |
| LysoPE(0:0/24:0) | 565.4107398 | C29H60NO7P | 548.4088372 |
| Neomycin | 614.3122856 | C23H46N6O13 | 579.2938648 |
| Herniarin | 176.0473441 | C10H8O3 | 177.0542927 |
| (14alpha,17beta,20S,22R)-14,20-Epoxy-17-hydroxy-1-oxowitha-3,5,24-trienolide | 452.2562743 | C28H36O5 | 507.2729195 |
| Pinolenic acid | 278.2245802 | C18H30O2 | 279.231254 |
| 2-Hydroxyestradiol | 288.1725446 | C18H24O3 | 321.2051989 |
| Anisaldehyde | 136.0524295 | C8H8O2 | 137.0595423 |
| Lpe(18:2) | 477.2855393 | C23H44NO7P | 478.2917438 |
| Myristoleic acid | 226.1932801 | C14H26O2 | 231.1736749 |
| Benzoic aldehyde | 106.0418648 | C7H6O | 107.0492659 |
| 5-kete | 318.2194948 | C20H30O3 | 319.2261398 |
| Exo,exo-1,8-Epoxy-p-menthane-2,6-diol | 186.1255944 | C10H18O3 | 191.1062374 |
| Mg(21:0/0:0/0:0) | 400.35526 | C24H48O4 | 418.3879945 |
| LysoPE(16:1(9Z)/0:0) | 451.2698892 | C21H42NO7P | 452.276042 |
| Arachidic acid | 312.3028305 | C20H40O2 | 330.335767 |
| [6]-Gingerdione | 292.1674593 | C17H24O4 | 275.1635961 |
| Palmitoyl Serinol | 329.2929941 | C19H39NO3 | 330.299501 |
| Stearidonic acid | 276.2089301 | C18H28O2 | 277.2155385 |
| Forskolin | 410.2304534 | C22H34O7 | 375.2132964 |
| Falcarinone | 242.1670653 | C17H22O | 275.1998923 |
| Isoelemicin | 208.1099444 | C12H16O3 | 191.1062324 |
| Benzyl acetate | 150.0680796 | C9H10O2 | 151.0750481 |
| Solasodine | 413.3293796 | C27H43NO2 | 413.3264241 |
| 8-Shogaol | 304.2038448 | C19H28O3 | 305.2102936 |
| Cannabidivarin | 286.1932801 | C19H26O2 | 287.1997736 |
| Sinapyl alcohol | 210.0892089 | C11H14O4 | 193.0855688 |
| [2-(4-Methylphenyl)-1,3-dioxolan-4-yl]methanol | 194.0942943 | C11H14O3 | 177.0906242 |
| 2-Methylbenzoic acid | 136.0524295 | C8H8O2 | 137.059493 |
| Indan-1-ol | 134.0731649 | C9H10O | 117.0698847 |
| Salmeterol | 415.2722587 | C25H37NO4 | 398.2679597 |
| Dehydrophytosphingosine | 315.2773441 | C18H37NO3 | 280.2627246 |
| Austalide L | 428.2198888 | C25H32O6 | 446.2525234 |
| Melleolide B | 432.2148034 | C24H32O7 | 415.2104533 |
| 2-Phenylpropanal | 134.0731649 | C9H10O | 135.0801959 |
| M-Xylene | 106.0782503 | C8H10 | 107.0855901 |
| Pitheduloside B | 882.4976863 | C46H74O16 | 882.4985995 |
| Tobramycin | 467.2591278 | C18H37N5O9 | 432.2496297 |
| Paxilline | 435.2409585 | C27H33NO4 | 418.2338464 |
| Alpha-Allocryptopine | 369.1576229 | C21H23NO5 | 387.19185 |
| Ricinoleic acid | 298.250795 | C18H34O3 | 316.2837723 |
| Cortisone | 360.193674 | C21H28O5 | 361.1975244 |
| Oryzalide B | 320.1987594 | C19H28O4 | 321.2051741 |
| Falcarindiol | 260.17763 | C17H24O2 | 261.1842503 |
| 4-Hydroxy-3-methoxybenzenemethanol | 154.0629942 | C8H10O3 | 137.0594849 |
| Avocadene | 286.250795 | C17H34O3 | 304.2837768 |
| [7]-Paradol | 292.2038448 | C18H28O3 | 275.199755 |
| 4-Methylumbelliferone | 176.0473441 | C10H8O3 | 177.0542318 |
| Mg(20:5(5z,8z,11z,14z,17z)/0:0/0:0) | 376.2613596 | C23H36O4 | 376.2584239 |
| Alpha-Crocetin glucosyl ester | 490.2202827 | C26H34O9 | 508.2528132 |
| 9,10-Dihydroxystearic acid | 316.2613596 | C18H36O4 | 334.2941631 |
| Clausarinol | 414.2042387 | C24H30O6 | 432.2368563 |
| Melleolide | 400.1885886 | C23H28O6 | 418.2211787 |
| Mycorradicin | 248.104859 | C14H16O4 | 249.1113635 |
| Phenylacetic acid | 136.0524295 | C8H8O2 | 119.0491178 |
| Styrene | 104.0626003 | C8H8 | 105.0700205 |
| Cyclohexanecarboxylic acid | 128.0837296 | C7H12O2 | 93.07011632 |
| N,N-Dimethyldodecylamine-N-oxide | - | C14H31NO | 230.2471833 |
| Ethyl myristate | 256.2402303 | C16H32O2 | 274.2732197 |
| Hydroxy-alpha-sanshool | 263.188529 | C16H25NO2 | 281.2214154 |
| Ginsenoyne C | 276.1725446 | C17H24O3 | 277.1790869 |
| 11-Hydroxy-9-tridecenoic acid | 228.1725446 | C13H24O3 | 229.1792131 |
| 5,7alpha-Dihydro-1,4,4,7a-tetramethyl-4H-indene | 174.1408506 | C13H18 | 175.1477781 |
| MG(a-15:0/0:0/0:0)[rac] | 316.2613596 | C18H36O4 | 334.2942827 |
| Cynaropicrin | 346.1416384 | C19H22O6 | 329.1375404 |
| N-Undecanoylglycine | 243.1834437 | C13H25NO3 | 244.1901233 |
| Alpha-Cyperone | - | C15H22O | 219.1738146 |
| 1-Methoxyindole-3-Carbaldehyde | 175.0633285 | C10H9NO2 | 176.070268 |
| Myristicin | 192.0786443 | C11H12O3 | 193.0855851 |
| Geranic Acid | 168.1150298 | C10H16O2 | 186.1485035 |
| Adipic acid | 146.0579088 | C6H10O4 | 129.0544762 |
| DG(8:0/0:0/PGF1alpha) | 556.3975188 | C31H56O8 | 557.4010718 |
| Withaperuvin C | 486.2617536 | C28H38O7 | 528.2969577 |
| KAPA | 187.1208434 | C9H17NO3 | 188.1277209 |
| Tranexamic acid | 157.1102787 | C8H15NO2 | 158.1172523 |
| (3b,16a,20R)-25-Acetoxy-3,16,20,22-tetrahydroxy-5-cucurbiten-11-one 3-glucoside | 710.4241274 | C38H62O12 | 733.4187304 |
| 4-ethynylaniline | - | C8H7N | 118.065082 |
| Pantothenic Acid | 219.110672659;219.110672659 | C9H17NO5 | 220.1173551 |
| Hydroquinone | 110.0367794 | C6H6O2 | 111.0440785 |
| Butanal | 72.05751488 | C4H8O | 73.06532793 |
| 1,2-Benzoquinone | 108.0211294 | C6H4O2 | 109.028462 |
| Homoserine | 119.0582432 | C4H9NO3 | 84.0447727 |
| 5-hydroxymethyl-2-furancarboxylic acid | 142.0266087 | C6H6O4 | 143.0336184 |
| L-Isoleucine | 131.0946287 | C6H13NO2 | 132.1016668 |
| 3-hydroxypyridine | - | C5H5NO | 96.0446207 |
| Gaba | 103.0633285 | C4H9NO2 | 86.06038261 |
| Adenosine Cyclophosphate | - | C10H12N5O6P | 330.0588638 |
| Adenosine | 267.096753929;267.096753929 | C10H13N5O4 | 268.1032287 |
| Urocanic Acid | 138.042927446;138.042927441 | C6H6N2O2 | 139.0498832 |
| Uracil | 112.0272774 | C4H4N2O2 | 113.034548 |
| Cytosine | 111.0432618 | C4H5N3O | 112.0505309 |
| 1-Kestose | 504.169035 | C18H32O16 | 527.1568981 |
| Sorbose | 180.063388116;360.126776213 | C6H12O6 | 163.0596768 |
| Levoglucosan | 162.0528234 | C6H10O5 | 145.0492232 |
| Furfural | 96.02112937 | C5H4O2 | 97.02862245 |
| 3-Feruloylquinic acid | 368.1107322 | C17H20O9 | 368.1120207 |
| Betaine | 118.0868036 | C5H11NO2 | 118.0861451 |
| 2,3-Butanediol | 90.06807956 | C4H10O2 | 73.06531091 |
| L(+)-Arginine | - | C6H14N4O2 | 175.1185077 |
| (S)-beta-Aminoisobutyric acid | 103.0633285 | C4H9NO2 | 104.0707013 |
| Pyrogallol | 126.0316941 | C6H6O3 | 109.0284365 |
| Triacetic acid | 144.0422587 | C6H8O4 | 145.0491788 |
| Mjdbissp00000001 | - | C9H10ClNO2 | 200.0467886 |
| Mjdbissp00000002 | - | C23H42D3NO4 | 403.3599985 |
| Mjdbissp00000009 | - | C9H6D5NO2 | 171.1171987 |
| Lucyoside M | 836.4558215 | C44H68O15 | 801.444372 |
| Trehalose | 342.1162115 | C12H22O11 | 341.1085387 |
| Sucrose | 342.1162115 | C12H22O11 | 377.0852895 |
| Citramalic acid | 148.0371734 | C5H8O5 | 129.0179093 |
| D-alpha-hydroxyglutaric acid | - | C5H8O5 | 147.0285715 |
| Xanthine | 152.0334254 | C5H4N4O2 | 151.0248282 |
| Uric Acid | 168.02834 | C5H4N4O3 | 167.0198165 |
| Gallic acid | 170.0215233 | C7H6O5 | 169.0130112 |
| Citric acid | 192.0270026 | C6H8O7 | 191.0187311 |
| Pseudouridine | 244.0695361 | C9H12N2O6 | 279.0385308 |
| 3',5'-Cyclic GMP | 345.0474343 | C10H12N5O7P | 344.0397715 |
| Guanosine | 283.0916686 | C10H13N5O5 | 282.0839791 |
| Ensulizole | - | C13H10N2O3S | 273.0338238 |
| Dg(pgd1/2:0/0:0) | 470.2879683 | C25H42O8 | 515.2833241 |
| 8-Amino-7-oxononanoic acid | 187.1208434 | C9H17NO3 | 186.1125612 |
| Xanthoxyline | 196.0735589 | C10H12O4 | 195.0653928 |
| D-3-phenyllactic acid | 166.0629942 | C9H10O3 | 165.0545312 |
| Ferulic Acid | 194.0579088 | C10H10O4 | 193.049715 |
| (-)-erythro-Anethole glycol 1-glucoside | 344.1471177 | C16H24O8 | 343.1396053 |
| Azelaic acid | 188.104859 | C9H16O4 | 187.0965909 |
| Alpha-L-Rhamnose | - | C6H12O5 | 163.059993 |
| Glucosyl (E)-2,6-Dimethyl-2,5-heptadienoate | 316.1522031 | C15H24O7 | 297.1341004 |
| 4-Nitrophenol | 139.026943 | C6H5NO3 | 138.0183531 |
| Furanogermenone | 232.1463299 | C15H20O2 | 291.1600521 |
| 1-Dehydro-[6]-gingerdione | 290.1518092 | C17H22O4 | 289.1443792 |
| 9(S)-HpODE | 312.230059512;312.23005951;312.230059512 | C18H32O4 | 311.2226191 |
| Cholic acid | 408.2875744 | C24H40O5 | 443.259765 |
| LysoPE(0:0/16:1(9Z)) | 451.2698892 | C21H42NO7P | 450.2624995 |
| Pe(16:1/0:0) | - | C21H42NO7P | 450.2624544 |
| (1(10)E,4E,6a,9b)-9-(2-Methylbutanoyloxy)-1(10),4,11(13)-germacratrien-12,6-olide | 332.1987594 | C20H28O4 | 367.166028 |
| 1-palmitoyl lysophosphatidic acid | - | C19H39O7P | 409.2358517 |
| Coriolic acid | - | C18H32O3 | 295.2274279 |
| Galanolactone | 318.2194948 | C20H30O3 | 363.2174258 |
| Cyclohexyl cinnamate | 230.1306798 | C15H18O2 | 505.2594621 |
| Ethyl benzoate | 150.0680796 | C9H10O2 | 149.0594628 |
| [6]-Dehydrogingerdione | 290.1518092 | C17H22O4 | 289.1442909 |
| Dehydrozingerone | 192.0786443 | C11H12O3 | 191.0703607 |
| 2-hydroxymyristic acid | 244.2038448 | C14H28O3 | 243.1959732 |
| Lpe(16:0) | 453.2855393 | C21H44NO7P | 452.2780524 |
| Norethindrone acetate | 340.2038448 | C22H28O3 | 339.1995531 |
| Squamostanal A | 294.2194948 | C18H30O3 | 315.1961169 |
| Andrographolide | 350.2093241 | C20H30O5 | 331.1912206 |
| 9-cis-Retinoic acid | 300.2089301 | C20H28O2 | 345.2067122 |
| Estradiol valerate | 356.2351449 | C23H32O3 | 401.2327512 |
| Mjdbissn00000004 | - | C5H12O5 | 151.0637081 |
| Demissidine | 464.3501746 | C28H48O5 | 499.3224208 |
| Cadabicine methyl ether | 449.2314565 | C26H31N3O4 | 448.2238483 |
| Dg(lte4/0:0/8:0) | 639.380489 | C34H57NO8S | 684.3750916 |
| Tetrahydrocannabinol | 314.2245802 | C21H30O2 | 373.2380239 |
| O-Geranylvanillin | 288.1725446 | C18H24O3 | 575.3375496 |
| 17-Hydroxylinolenic acid | 294.2194948 | C18H30O3 | 315.1960951 |
| Mg(22:5(4z,7z,10z,13z,16z)/0:0/0:0) | 404.2926598 | C25H40O4 | 425.2696438 |
| P-Tolyl acetate | 150.0680796 | C9H10O2 | 149.0594665 |
| [10]-Dehydrogingerdione | 346.2144094 | C21H30O4 | 345.2067125 |
| 2,4,6-Trihydroxybenzoic acid | 170.0215233 | C7H6O5 | 169.0130191 |
| Kanzonol M | 398.1729386 | C23H26O6 | 379.1578696 |
| Heptadecanal | 254.2609657 | C17H34O | 313.2745818 |
| Hebevinoside IV | 604.4339043 | C36H60O7 | 625.4108404 |
| Alpha-Irone | 206.1670653 | C14H22O | 457.3321219 |
| (r)-2-hydroxystearic acid | - | C18H36O3 | 299.2587625 |
| Maslinic acid | 472.35526 | C30H48O4 | 531.3688863 |
| Oryzanol A | 602.4335103 | C40H58O4 | 661.4470933 |
| Ethotoin | 204.0898776 | C11H12N2O2 | 239.0591388 |
| Glyceric Acid | 106.0266087 | C3H6O4 | 165.0396013 |
| Stearaldehyde | 268.2766158 | C18H36O | 313.274637 |
| 12s-hht | 280.2038448 | C17H28O3 | 605.4059534 |
| Nonadecanoic acid | 298.2871805 | C19H38O2 | 343.2849527 |
| Grifolin | 328.2402303 | C22H32O2 | 373.2380702 |
| 2-Hydroxy-4-oxo-5,12-heneicosadien-1-yl acetate | 380.2926598 | C23H40O4 | 401.269305 |
| Cerebronic acid | 384.3603454 | C24H48O3 | 383.3527374 |
| 16-hydroxyhexadecanoic acid | 272.2351449 | C16H32O3 | 271.2276133 |
| Auraptene | 298.1568946 | C19H22O3 | 297.1525798 |
| Ganoderic acid Ma | 572.371304 | C34H52O7 | 593.3482823 |
| Vignatic acid B | 519.2944507 | C27H41N3O7 | 554.2674795 |
| 2-Methyl-5-(8,11-pentadecadienyl)-1,3-benzenediol | 330.2558803 | C22H34O2 | 375.2537072 |
| 3-(8,11,14-Pentadecatrienyl)phenol | 298.2296656 | C21H30O | 343.2273056 |
| [8]-Dehydrogingerdione | 318.1831093 | C19H26O4 | 317.1753953 |
| 2-methoxy-4-vinylphenol | 150.0680796 | C9H10O2 | 149.0594503 |
| Estriol | 288.1725446 | C18H24O3 | 287.1649943 |
| Lpe(18:1) | 479.3011893 | C23H46NO7P | 478.2935679 |
| 1-Acetoxy-2-hydroxy-16-heptadecen-4-one | 326.2457096 | C19H34O4 | 347.2223717 |
| Pe(17:1/0:0) | - | C22H44NO7P | 464.2778463 |
| Carnosic acid | 332.1987594 | C20H28O4 | 331.1911735 |
| Pe(18:2/0:0) | - | C23H44NO7P | 476.2780014 |
| Palmitic Acid | 256.2402303 | C16H32O2 | 255.232556 |
| (S)-Laudanosine | 357.1940084 | C21H27NO4 | 392.1615219 |
| Panaquinquecol 6 | 318.1831093 | C19H26O4 | 317.1754933 |
| Idebenone | 338.2093241 | C19H30O5 | 319.1911067 |
| 6-hydroxy-2-aminopurine | 151.0494098 | C5H5N5O | 150.0408186 |
| Elenolide | 224.0684735 | C11H12O5 | 447.1341401 |
| 10-Hydroxy-8-nor-2-fenchanone glucoside | 316.1522031 | C15H24O7 | 297.1341254 |
| 5-Phenyl-1,3-oxazinane-2,4-dione | 191.0582432 | C10H9NO3 | 226.026932 |
| 4-Hydroxycinnamoylagmatine | 276.1586259 | C14H20N4O2 | 297.1341135 |
| Suberic acid | 174.0892089 | C8H14O4 | 173.0808186 |
| Acetylleucine | 173.1051934 | C8H15NO3 | 172.096809 |
| 2-isopropylmalic acid | 176.0684735 | C7H12O5 | 175.0601054 |
| Guanosine 5'-monophosphate | 363.057999 | C10H14N5O8P | 344.0397354 |
| N-Acetylmuramate | 293.1110666 | C11H19NO8 | 274.0929642 |
| Uridine | 244.0695361 | C9H12N2O6 | 243.0616272 |
| Chrysoobtusin | 358.1052529 | C19H18O7 | 379.0820785 |
| L-galactose | 180.0633881 | C6H12O6 | 215.0318656 |
| Gluconolactone | 178.0477381 | C6H10O6 | 177.0392901 |
| Mjdbissn00000003 | - | C24H36D4O5 | 411.3052695 |
| Mjdbissn00000001 | - | C9H10ClNO2 | 198.031792 |
| Mjdbissn00000009 | - | C9H6D5NO2 | 169.1018948 |
| Pa(18:1(12z)-2oh(9,10)/10:0) | 622.3845851 | C31H59O10P | 657.3563796 |
| D-Mannose | 180.0633881 | C6H12O6 | 179.0549632 |

**Table S2.** The top ten components in the GELN-human RCC target network

| **Compound name** | **Molecular formula** | **2D structure** | **Degree** |
| --- | --- | --- | --- |
| 6-shogaol | C_17_H_24_O_3_ | 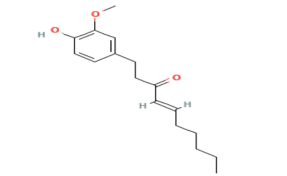 | 63 |
| [6]-Dehydrogingerdione | C_17_H_22_O_4_ | 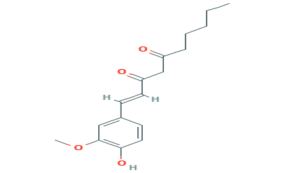 | 61 |
| [6]-Gingerdione | C_17_H_24_O_4_ | 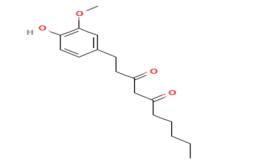 | 60 |
| 6-Paradol | C_17_H_26_O_3_ | 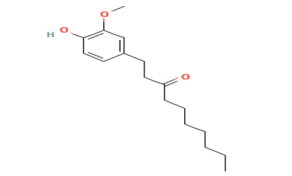 | 60 |
| 12s-hht | C_17_H_28_O_3_ | 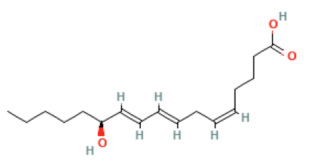 | 59 |
| Mg(20:5(5z,8z,11z,14z,17z/0:0/0:0) | C_23_H_36_O_4_ | 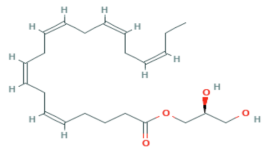 | 58 |
| 6-Hydroxyshogaol | C_17_H_24_O_4_ | 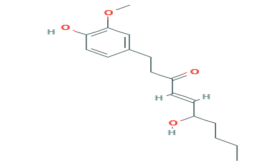 | 57 |
| [8]-Dehydrogingerdione | C_19_H_26_O_4_ | 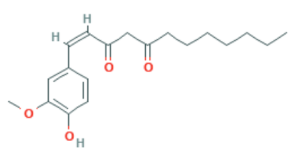 | 57 |
| Cadabicine methyl ether | C_26_H_31_N_3_O_4_ | 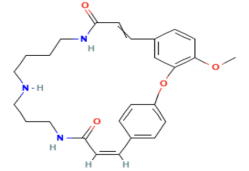 | 54 |
| Panaquinquecol 6 | C_19_H_26_O_4_ | 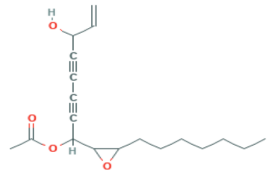 | 54 |

**Table S3.** The top ten components in the GELN-mouse RCC target network

| **Compound name** | **Molecular formula** | **2D structure** | **Degree** |
| --- | --- | --- | --- |
| (3beta,5alpha,9alpha,22E,24R)-3,5,9-Trihydroxy-23-methylergosta-7,22-dien-6-one | C_29_H_46_O_4_ | 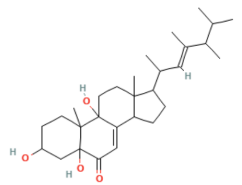 | 99 |
| Cadabicine methyl ether | C_26_H_31_N_3_O_4_ | 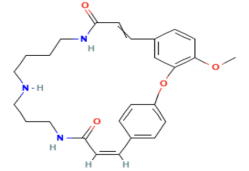 | 98 |
| Frangulanine | C_28_H_44_N_4_O_4_ | 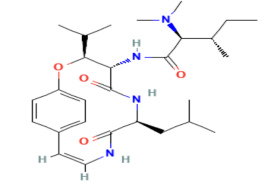 | 93 |
| Mg(20:5(5z,8z,11z,14z,17z/0:0/0:0) | C_23_H_36_O_4_ | 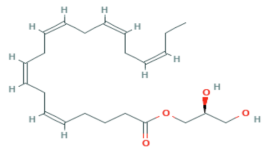 | 91 |
| [6]-Gingerdione | C_17_H_24_O_4_ | 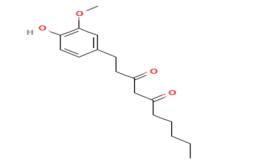 | 91 |
| [8]-Dehydrogingerdione | C_19_H_26_O_4_ | 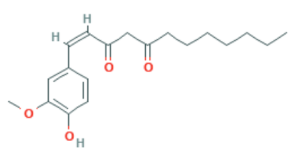 | 86 |
| 6-shogaol | C_17_H_24_O_3_ | 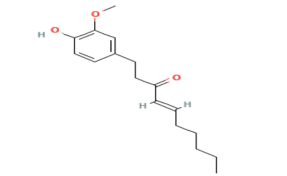 | 82 |
| 6-Paradol | C_17_H_26_O_3_ | 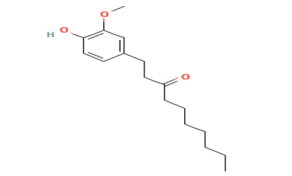 | 76 |
| Ricinoleic acid | C_18_H_34_O_3_ | 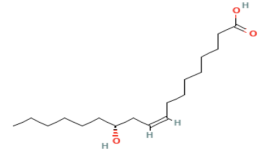 | 75 |
| 12s-hht | C_17_H_28_O_3_ | 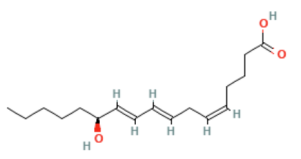 | 74 |

**Table S4.** Information on the top ten nodes in human gene-gene interaction network

| **Target name** | **Degree** | **ASPL** | **BC** | **CC** |
| --- | --- | --- | --- | --- |
| TP53 | 75 | 2.19 | 0.15 | 0.46 |
| SRC | 59 | 2.36 | 0.07 | 0.42 |
| AKT1 | 53 | 2.27 | 0.05 | 0.44 |
| PIK3R1 | 52 | 2.55 | 0.02 | 0.39 |
| PIK3CA | 51 | 2.43 | 0.02 | 0.41 |
| STAT3 | 50 | 2.3 | 0.06 | 0.44 |
| HSP90AA1 | 50 | 2.26 | 0.07 | 0.44 |
| PIK3CB | 46 | 2.64 | 0.01 | 0.38 |
| PIK3CD | 45 | 2.65 | 0.01 | 0.38 |
| GRB2 | 42 | 2.7 | 0.02 | 0.37 |

**Table S5.** Information on the top ten nodes in mouse gene-gene interaction network

| **Target name** | **Degree** | **ASPL** | **BC** | **CC** |
| --- | --- | --- | --- | --- |
| Stat3 | 40 | 2.58 | 0.13 | 0.39 |
| Akt1 | 39 | 2.53 | 0.14 | 0.4 |
| Hsp90aa1 | 31 | 2.87 | 0.07 | 0.35 |
| Pik3ca | 27 | 2.93 | 0.03 | 0.34 |
| Egfr | 25 | 2.93 | 0.05 | 0.34 |
| Ptgs2 | 25 | 2.9 | 0.01 | 0.34 |
| Mapk3 | 24 | 2.83 | 0.05 | 0.35 |
| Tlr4 | 23 | 2.88 | 0.08 | 0.35 |
| Esr1 | 22 | 2.85 | 0.04 | 0.35 |
| Mapk14 | 21 | 2.96 | 0.03 | 0.34 |

**Table S6.** Coordinates of the target gene's active pocket and associated grid dimensions

| **Target gene** | **PDB ID** | **Active pocket coordinates** | **Grid dimensions** |
| --- | --- | --- | --- |
| AKT1 | 1UNQ | X=23.033, Y=15.078, Z=8.102 | X=44, Y=40, Z=48 |
| PIK3CA | 6VO7 | X=11.835, Y=22.39, Z=-10.476 | X=56, Y=54, Z=48 |
| PIK3R1 | 2IUG | X=10.191, Y=33.868, Z=-23.144 | X=42, Y=42, Z=52 |
| SRC | 7NG7 | X=-10.294, Y=-1.68, Z=-12.823 | X=40, Y=40, Z=48 |
| TP53 | 3D06 | X=3.31, Y=16.195, Z=-13.301 | X=38, Y=50, Z=50 |
| ABCB1 | 6C0V | X=163.81, Y=167.261, Z=153.151 | X=58, Y=86, Z=126 |

**Table S7.** Binding energies of the top five CHGs and ABCB1 with their respective compounds

| **Receptor name**  **Binding energy (kcal mol^-1^)**  **Compound name** | **AKT1** | **PIK3CA** | **PIK3R1** | **SRC** | **TP53** | **ABCB1** |
| --- | --- | --- | --- | --- | --- | --- |
| [6]-Dehydrogingerdione | -5.1 |  | -5.0 |  |  |  |
| [8]-Dehydrogingerdione | -4.9 |  |  |  |  |  |
| [10]-Dehydrogingerdione | -5.0 |  |  |  |  |  |
| [10]-Dehydroshogaol | -4.6 |  |  |  |  |  |
| 2-aminooctadecane-1 | -4.0 |  |  |  |  |  |
| Aminopentol | -4.9 | -4.4 |  |  |  |  |
| Cadabicine methyl ether | -7.8 |  |  | -9.3 | -9 |  |
| N,n-dimethylsphingosine | -4.0 |  |  |  |  | -4.4 |
| O-Arachidonoyl Ethanolamine | -4.0 |  |  |  |  |  |
| Palmitoyl Serinol | -4.5 |  |  |  |  |  |
| Vignatic acid B | -6.8 | -6.2 |  | -8.3 |  |  |
| (3β,5α,9α,22E,24R)-3,5,9-Trihydroxy |  | -6.7 |  |  |  |  |
| [6]-Gingerdione |  | -5.2 |  | -7.6 |  |  |
| [8]-Paradol |  | -5.0 |  | -7.8 |  |  |
| [10]-Shogaol |  | -4.4 |  | -7.1 |  |  |
| 2-Methyl-5-(8,11-pentadecadieny) |  | -5.0 |  | -7.3 |  |  |
| 4-Hydroxycinnamoylagmatine |  | -5.5 |  |  |  | -7.3 |
| 5-kete |  | -4.5 |  |  |  |  |
| 6-Hydroxyshogaol |  | -4.8 | -4.9 |  |  |  |
| 6-Paradol |  | -5.0 |  | -7.2 |  |  |
| 6-shogaol |  | -5.1 |  | -7.4 |  |  |
| 8-Shogaol |  | -5.1 |  | -8.0 |  |  |
| 9,10-Dihydroxystearic acid |  | -4.8 |  |  |  |  |
| 9-oxootre |  | -4.6 |  |  |  |  |
| 17-Hydroxylinolenic acid |  | -4.5 | -4.3 |  |  |  |
| Hebevinoside IV |  | -6.9 |  |  |  |  |
| Mg(205(5z,8z,11z,14z,17z)0000) |  | -4.7 |  |  |  |  |
| Panaquinquecol 6 |  | -4.5 |  | -6.9 |  |  |
| [7]-Paradol |  |  |  | -7.3 |  |  |
| [10]-Paradol |  |  |  | -6.8 |  |  |
| 2-Butyl-5-[2-(4-hydroxy-3-methoxyphenyl) |  |  |  | -8.1 |  | -7.0 |
| 3-(8,11,14-Pentadecatrienyl)phenol |  |  |  | -7.1 |  |  |
| Coriolic acid |  |  |  | -5.6 | -4.8 |  |
| 12s-hht |  |  |  |  | -4.5 |  |
| Gallic acid |  |  |  |  | -6.0 |  |
| 2-[2-(4-Hydroxy-3-methoxyphenyl)ethyl]-5-octylfuran |  |  |  |  |  | -7.2 |
| 9,12-octadecadiynoic acid |  |  |  |  |  | -4.6 |
| Falcarinone |  |  |  |  |  | -5.1 |
| Ferulic Acid |  |  |  |  |  | -6.5 |
| Frangulanine |  |  |  |  |  | -6.6 |
| Ganoderic acid Ma |  |  |  |  |  | -8.8 |
| Palmitic Acid |  |  |  |  |  | -6.5 |
| Panaquinquecol 6 |  |  |  |  |  | -6.0 |

**Table S8.** IC50 values for the effect of GELNs on cell viability (μg ml^-1^)

|  | **24h** | **48h** |
| --- | --- | --- |
| RenCa | 26.29 | 13.32 |
| 786-O | 23.7 | 12.42 |
| OS-RC-2 | 37.47 | 18.72 |
| HK2 | 95.25 | 60.89 |

**Table S9.** EE (%) and DL (%) of Su and GELNs under different mass ratios

| **Su mass (mg)** | **GELNs mss (mg)** | **EE (%)** | **DL (%)** |
| --- | --- | --- | --- |
| 0.1 | 10 | 70.5 ± 0.6 | 0.93 ± 0.01 |
| 0.5 | 10 | 67.41 ± 0.25 | 4.39 ± 0.02 |
| 1 | 10 | 66.98 ± 0.30 | 8.02 ± 0.04 |
| 1.5 | 10 | 61.69 ± 0.17 | 10.92 ± 0.03 |
| 2.0 | 10 | 47.23 ± 0.28 | 11.01 ± 0.06 |
| 2.5 | 10 | 36.98 ± 0.24 | 11.09 ± 0.06 |

**Table S10.** Characterization data for nanoformulations

| **Nanoparticles** | **Size (nm)** | **PDI** | **Zeta potential (mV)** | **EE%** | **DL%** |
| --- | --- | --- | --- | --- | --- |
| GELNs | 122.5 ± 23.45 | 0.299 | -20.4 ± 5.84 |  |  |
| GELNs/Su | 123.9 ± 20.43 | 0.282 | -21.2 ± 5.15 | 61.69 ± 0.17 | 10.92 ± 0.03 |
| FPD-GELNs | 129.2 ± 22.60 | 0.232 | -25.2 ± 5.43 |  |  |
| FPD-GELNs/Su | 131.7 ± 27.91 | 0.28 | -25.6 ± 4.63 | 49.93 ± 0.19 | 10.29 ± 0.02 |

**Table S11.** Characterization changes in nanoformulations over a three-month period

| **Nanoparticles** | **Time (month)** | **Size (nm)** | **PDI** | **Zeta potential (mV)** | **DL%** |
| --- | --- | --- | --- | --- | --- |
|  | 0 | 123.9 ± 20.43 | 0.282 | -21.2 ± 5.15 | 10.92 ± 0.03 |
| GELNs/Su | 1.5 | 120.3 ± 17.71 | 0.370 | -21.2 ± 7.46 | 10.81 ± 0.03 |
|  | 3 | 128.8 ± 22.97 | 0.334 | -21.8 ± 9.34 | 10.75 ± 0.03 |
|  | 0 | 131.7 ± 27.91 | 0.28 | -25.6 ± 4.63 | 10.29 ± 0.02 |
| FPD-GELNs/Su | 1.5 | 133.7 ± 25.34 | 0.283 | -27.0 ± 7.89 | 10.32 ± 0.07 |
|  | 3 | 133.4 ± 27.22 | 0.367 | -26.0 ± 6.73 | 10.20 ± 0.05 |

**Table S12.** CI value and its corresponding drug synergy evaluation scale

| **CI** | **Description** |
| --- | --- |
| < 0.1 | Very strong synergism |
| 0.1-0.3 | Strong synergism |
| 0.3-0.7 | Synergism |
| 0.7-0.85 | Moderate Synergism |
| 0.85-0.9 | Slight Synergism |
| 0.9-1.1 | Nearly addictive |
| 1.1-1.2 | Slight antagonism |
| 1.2-1.45 | Moderate antagonism |
| 1.45-3.3 | Antagonism |
| 3.3-10 | Strong antagonism |
| > 10 | Very strong antagonism |

**Table S13.** The top ten components in the GELN - macrophage polarization target network

| **Compound name** | **Molecular formula** | **Degree** |
| --- | --- | --- |
| Cadabicine methyl ether | C_26_H_31_N_3_O_4_ | 22 |
| Frangulanine | C_28_H_44_N_4_O_4_ | 21 |
| 2-Butyl-5-[2-(4-hydroxy-3-methoxyphenyl)ethyl]furan | C₁₇H₂₂O₃ | 18 |
| [6]-Gingerdione | C₁₇H₂₄O₄ | 17 |
| (3beta,5alpha,9alpha,22E,24R)-3,5,9-Trihydroxy-23-methylergosta-7,22-dien-6-one | C₂₉H₄₄O₄ | 16 |
| [7]-Paradol | C₁₇H₂₆O₃ | 16 |
| 12s-hht | C₂₀H₃₂O₄ | 15 |
| [6]-Dehydrogingerdione | C₁₇H₂₂O₄ | 15 |
| 2-[2-(4-Hydroxy-3-methoxyphenyl)ethyl]-5-octylfuran | C₂₁H₃₀O₄ | 14 |
| [8]-Dehydrogingerdione | C₁₉H₂₆O₄ | 13 |

**Table S14.** The top ten miRNA in the GELN - macrophage polarization target network

| **miRNA** | **Degree** |
| --- | --- |
| mes-miR477a | 6 |
| mes-miR477b | 6 |
| mes-miR477c | 6 |
| mes-miR477e | 6 |
| nta-miR477a | 4 |
| osa-miR530-5p | 4 |
| gma-miR164b | 3 |
| gma-miR164c | 3 |
| gma-miR164d | 3 |
| mes-miR164d | 3 |

**Table S15.** Pharmacokinetic parameters of Su in plasma

| **Sample** | **C_max_**  **(ng ml^-1^)** | **T_1/2, β_**  **(h)** | **AUC_0-24_**  **(ng.h ml^-1^)** | ****AUC₀–∞****  **(ng.h ml^-1^)** |
| --- | --- | --- | --- | --- |
| Su | 121.7 | 4.86 | 114.4 | 114.8 |
| FPD-GELNs/Su | 190.0 | 5.97 | 533.1 | 546.9 |

**Table S16.** Pharmacokinetic parameters of Su in tumor

| **Sample** | **C_max_**  **(ng g^-1^)** | **T_1/2, β_**  **(h)** | **AUC_0-24_**  **(ng.h g^-1^)** | ****AUC₀–∞****  **(ng.h g^-1^)** |
| --- | --- | --- | --- | --- |
| Su | 563.1 | 6.3 | 3342 | 3350 |
| FPD-GELNs/Su | 1598.6 | 7.2 | 17916 | 17950 |

**Table S17.** Gradient optimization of the mobile phase for 6-Shogaol, 8-Shogaol, and 6-Paradol.

| **Time（min）** | **A%** | **B%** |
| --- | --- | --- |
| 0 | 98 | 2 |
| 2 | 98 | 2 |
| 4 | 60 | 40 |
| 5 | 50 | 50 |
| 10 | 5 | 95 |
| 13 | 5 | 95 |
| 13.1 | 98 | 2 |
| 16 | 98 | 2 |

**Table S18.** Gradient optimization of the mobile phase for Ferulic acid.

| **Time（min）** | **A%** | **B%** |
| --- | --- | --- |
| 0 | 98 | 2 |
| 0.5 | 98 | 2 |
| 3.5 | 75 | 25 |
| 5 | 65 | 35 |
| 7 | 5 | 95 |
| 10 | 5 | 95 |
| 10.1 | 98 | 2 |
| 13 | 98 | 2 |

**Table S19.** The primer sequences for the key genes used in RT-qPCR analysis in this study

| **Species** | **Gene** | **Orientation** | **Primer sequence (5’ to 3’)** |
| --- | --- | --- | --- |
| Mouse | FOLR1 | Forward | CTGCTGCTCTGTGTGAGGAA |
|  |  | Reverse | TCATGGCCTCGGCATAGAAC |
| Mouse | FOLR2 | Forward | CCAGCAAGTGGACCAGAGTT |
|  |  | Reverse | CAGTCCCAGCCTTTATGCCA |
| Mouse | CD86 | Forward | TCTGCCGTGCCCATTTACAA |
|  |  | Reverse | TGTGCCCAAATAGTGCTCGT |
| Mouse | CD206 | Forward | CTCTGTTCAGCTATTGGACGC |
|  |  | Reverse | CGGAATTTCTGGGATTCAGCTTC |
| Mouse | iNOS | Forward | GGAGCGAGTTGTGGATTGTC |
|  |  | Reverse | GTGAGGGCTTGGCTGAGTGAG |
| Mouse | Arg1 | Forward | GTACATTGGCTTGCGAGACG |
|  |  | Reverse | GCCAATCCCCAGCTTGTCTA |
| Mouse | IL-6 | Forward | GAGGATACCACTCCCAACAGACC |
|  |  | Reverse | AAGTGCATCATCGTTGTTCATACA |
| Mouse | IL-10 | Forward | CCTGGGTGAGAAGCTGAAGAC |
|  |  | Reverse | TGTAGACACCTTGGTCTTGGA |
| Mouse | TNF-α | Forward | ACTGAACTTCGGGGTGATCG |
|  |  | Reverse | CCACTTGGTGGTTTGTGAGT |
| Mouse | TGF-β | Forward | ACTGGAGTTGTACGGCAGTG |
|  |  | Reverse | GGGCTGATCCCGTTGATTTC |
| Mouse | ABCB1 | Forward | TTCTCTTTGTCCGCGGAGTC |
|  |  | Reverse | CCTTCTTACTCCATTCCCCCTTT |
| Mouse | GAPDH | Forward | AGGTTGTCTCCTGCGACTTCA |
|  |  | Reverse | TGGTCCAGGGTTTCTTACTCC |
| Human | FOLR1 | Forward | GGAGGTGGCGAGGTTCTATG |
|  |  | Reverse | CAGCATTAGGGCCAGGCTAA |
| Human | FOLR2 | Forward | GCTTCTGGTCTGTGTAGCCA |
|  |  | Reverse | GTCTTGTGGTGCTTGGCATC |
| Human | ABCB1 | Forward | AAGCAACCAGATAAAAGAGAGGTG |
|  |  | Reverse | GTTCCTGCCCAGCCAATCA |
| Human | GAPDH | Forward | GCACCGTCAAGGCTGAGAAC |
|  |  | Reverse | TGGTGAAGACGCCAGTGGA |

**Table S20.** Gradient optimization of the mobile phase for Su.

| **Time（min）** | **A%** | **B%** |
| --- | --- | --- |
| 0 | 90 | 10 |
| 0.5 | 90 | 10 |
| 2 | 5 | 95 |
| 4.5 | 5 | 95 |
| 4.51 | 90 | 10 |
| 6 | 90 | 10 |
